# Synthesis and biological evaluation of celastrol derivatives as potent antitumor agents with STAT3 inhibition

**DOI:** 10.1080/14756366.2021.2001805

**Published:** 2021-12-11

**Authors:** Shaohua Xu, Ruolan Fan, Lu Wang, Weishen He, Haixia Ge, Hailan Chen, Wen Xu, Jian Zhang, Wei Xu, Yaqian Feng, Zhimin Fan

**Affiliations:** a College of Pharmacy, Fujian University of Traditional Chinese Medicine, Fuzhou, P.R. China; b National Center of Colorectal Disease, Nanjing Hospital of Chinese Medicine Affiliated to Nanjing University of Chinese Medicine, Nanjing, P.R. China; c Biology Department, Boston College, Brighton, MA, USA; d School of Life Sciences, Huzhou University, Huzhou, P.R. China; e State Key Laboratory of Natural Medicines, China Pharmaceutical University, Nanjing, P.R. China; f School of Pharmaceutical Sciences, Key Laboratory of Bioorganic Phosphorus Chemistry and Chemical Biology (Ministry of Education), Tsinghua University, Beijing, P.R. China

**Keywords:** Celastrol, structural modification, anti-tumour, STAT3 inhibitor, colorectal cancer organoid

## Abstract

Using STAT3 inhibitors as a potential strategy in cancer therapy have attracted much attention. Recently, celastrol has been reported that it could directly bind to and suppress the activity of STAT3 in the cardiac dysfunction model. To explore more effective STAT3 inhibiting anti-tumour drug candidates, we synthesised a series of celastrol derivatives and biologically evaluated them with several human cancer cell lines. The western blotting analysis showed that compound **4 m**, the most active derivative, could suppress the STAT3’s phosphorylation as well as its downstream genes. SPR analysis, molecular docking and dynamics simulations’ results indicated that the **4m** could bind with STAT3 protein more tightly than celastrol. Then we found that the **4m** could block cell-cycle and induce apoptosis on HCT-116 cells. Furthermore, the anti-tumour effect of **4m** was verified on colorectal cancer organoid. This is the first research that discovered effective STAT3 inhibitors as potent anti-tumour agents from celastrol derivatives.

## Introduction

Celastrol (**CEL**, also named tripterine), is a natural friedelane pentacyclic triterpenoid isolated from *Tripterygium wilfordii*^1^. It exhibits multiple pharmacological potentials, including anti-cancer, anti-inflammatory, and anti-obesity effects^2^^,^^3^. In 2007, celastrol was voted as one of the five most promising natural products for turning traditional medicines into modern drugs in *Cell*^4^. Scientists discovered new mechanisms of celastrol’s tumour-suppressing effects in recent decades by identifying various new pathways and key targets^5^^,^^6^. For instance, celastrol was found to suppress growth and induce apoptosis of human hepatocellular carcinoma through the STAT3/JAK2 signalling pathway ^7^. Other than that, structural modifications of celastrol also made significant progress, and various celastrol derivatives have been synthesised with biological activity evaluation^8–10^.

STAT3 is a cytoplasmic transcription factor that modulates many genes’ transcription activity to regulate critical biological functions, including cell proliferation, differentiation, cell survival, angiogenesis, and immune response^11^. Abnormal STAT3 activation (such as phosphorylation) plays a crucial role in tumour cell proliferation^12^. Identifying specific and potent STAT3 inhibitors as a candidate substance in cancer therapy has attracted much attention^13–16^. Recent research showed that celastrol could inhibit Ang II-induced cardiac dysfunction via directly binding to STAT3 instead of the upstream mediator^17^. Thus, STAT3 is speculated to be one of the main targets of celastrol for suppressing tumours. Furthermore, it is theoretically feasible to screen novel small molecule inhibitors of STAT3 from celastrol derivatives.

Organoids are microscopic self-organising, three-dimensional (3 D) structures grown from stem cells *in vitro*^18^. These amazing 3D constructs represent a promising, physiologically similar model for human cancers, which laid a foundation for various experimental applications in cancer research^19^. Every organoid culture derived from each patient showed incredible resemblance to the original tumours, making it the most clinically relevant and representational model for novel medicine development research^20^. In comparison, traditional pre-clinical models such as cell lines and animal models do not truly represent the original human tumour, making clinical experiments have a greater chance of failure and poor therapeutic performance^21^. So patient-derived organoids are widely considered to serve as a better platform for anti-tumour candidate drug screening and efficacy evaluation in recent years.

In order to explore more effective anti-tumour drug candidates and establish a more consistent structure-activity relationship (SAR) study on the anticancer activity of the A/E-ring modifications on celastrol derivatives, we synthesised a series of celastrol derivatives and investigated their anti-proliferation activities in three human cancer cell lines (A549, HCT-116, HepG2) *in vitro*. We also predicted their pharmacokinetic parameters via *in silico* ADMET analysis. Then we performed western blotting experiments targeting the JAK2/STAT3 pathway, as well as SPR detection, molecular docking analysis and molecular dynamics simulations with STAT3 protein, to investigate the function of the most active compound **4m** as the STAT3 inhibitor and reveal the primary mechanism of its anti-cancer effects. Furthermore, we assessed apoptosis and cell-cycle arrest effect of **4m**. In the end, the activity of **4m** was verified in human colorectal cancer organoid culture.

## Experimental

### Chemistry

Celastrol was purchased from Nanjing jingzhu bio-technology Co., Ltd, with over 98% purity. All commercially available reagents and solvent were purchased from Energy Chemical (Shanghai, China) and Shanghai Aladdin Biochemical Technology Co., Ltd. and used without further purification unless otherwise stated. ^1^H and ^13^C spectra were recorded in CDCl_3_ or CD_3_OD on a Bruker AV600 spectrometer (Switzerland) with tetramethylsilane (TMS) as an internal standard. Chemical shifts are expressed in *δ* (ppm) units downfield from TMS. All coupling constants (*J* - values) were reported in Hertz (Hz). High-resolution mass spectra (HR-MS) were obtained using a Water Q-Tof micro mass spectrometer. Column chromatography was performed with silica gel 60 (200–300 mesh). All tested compounds have a purity ≥ 95%.

#### General procedures for synthesising compound 1a–1d

A mixture of celastrol (5 g, 11.1 mmol), NaHCO_3_ (2.8 g, 33.3 mmol) and 3-allyl bromide (4.76 ml, 55.5 mmol) in DMF (20 ml) was stirred at room temperature for 12 h. After confirming the progress of the reaction by a thin-layer chromatography (TLC), the mixture was concentrated under vacuum. Then the residue was dissolved in 250 ml water and 250 ml EA, the EA layer was washed with water (250 ml × 3) and dried over anhydrous MgSO_4_. Then purified by normal phase column chromatography (PE/EA = 10:1) to afford the target compound **1a** as a red solid (4 g, 80%). Compounds **1b** was prepared according to the procedures same as **1a** except for replacing DMF with acetone as solvent. Compounds **1c** and **1d** come through the same synthetic route as **1a**.

##### Allyl 3-hydroxy-9β,13α-dimethyl-2-oxo-24,25,26-trinoroleana-1(10),3,5,7-tetraen-29-oate (1a)

Red powder; yield 80%; HR-ESI-MS *m/z*: 491.3165 [M + H]^+^ (calcd for C_32_H_43_O_4_, 491.3156); ^1^H NMR (600 MHz, CDCl_3_) *δ* 7.03 (dd, *J* = 7.1, 1.4 Hz, 1H), 6.54 (d, *J* = 1.4 Hz, 1H), 6.35 (d, *J* = 7.2 Hz, 1H), 5.86 (ddt, *J* = 17.3, 10.5, 5.6 Hz, 1H), 5.32–5.27 (m, 1H), 5.21 (dq, *J* = 10.5, 1.3 Hz, 1H), 4.47–4.37 (m, 2H), 2.21 (s, 3H), 1.45 (s, 3H), 1.26 (s, 3H), 1.20 (s, 3H), 1.10 (s, 3H), 0.55 (s, 3H); ^13^C NMR (150 MHz, CDCl_3_) *δ* 178.41, 177.98, 170.50, 164.97, 146.15, 134.46, **132.14**, 127.54, 119.61, 118.31, **118.25,** 117.41, **65.19**, 45.24, 44.42, 43.16, 40.58, 39.60, 38.39, 36.50, 34.90, 33.59, 32.90, 31.74, 30.94, 30.69, 29.98, 29.73, 28.79, 21.77, 18.66, 10.41.

##### Methyl 3-hydroxy-9β,13α-dimethyl-2-oxo-24,25,26-trinoroleana-1(10),3,5,7-tetraen-29-oate (1 b, Pristimerin)

Red powder; yield 51%; HR-ESI-MS *m/z*: 465.3029 [M + H]^+^ (calcd for C_30_H_41_O_4_, 465.2999); ^1^H NMR (600 MHz, CDCl_3_) *δ* 7.03 (dd, *J* = 7.1, 1.4 Hz, 1H), 6.54 (d, *J* = 1.5 Hz, 1H), 6.35 (d, *J* = 7.2 Hz, 1H), 3.55 (s, 3H), 2.42 (d, *J* = 16.0 Hz, 1H), 2.21 (s, 4H), 2.16 (ddd, *J* = 13.7, 4.8, 2.1 Hz, 1H), 2.05 (td, *J* = 14.1, 4.1 Hz, 1H), 1.89–1.84 (m, 2H), 1.60–1.57 (m, 1H), 1.45 (s, 3H), 1.26 (s, 3H), 1.17 (s, 3H), 1.10 (s, 3H), 0.53 (s, 3H); ^13^C NMR (150 MHz, CDCl_3_) *δ* 178.89, 178.37, 170.54, 164.98, 146.15, 134.44, 127.54, 119.65, 118.32, 117.42, **51.71**, 45.23, 44.45, 43.15, 40.56, 39.58, 38.42, 36.52, 34.94, 33.71, 32.82, 31.74, 31.05, 30.68, 30.02, 29.80, 28.79, 21.75, 18.48, 10.41.

##### 2-Methylbenzyl 3-hydroxy-9β,13α-dimethyl-2-oxo-24,25,26-trinoroleana-1(10),3,5,7-tetraen-29-oate (1c)

Red powder; yield 73%; HR-ESI-MS *m/z*: 555.3485 [M + H]^+^ (calcd for C_37_H_47_O_4_, 555.3469); ^1^H NMR (600 MHz, CDCl_3_) *δ* 7.23 (d, *J* = 7.2 Hz, 2H), 7.18 (d, *J* = 6.2 Hz, 1H), 7.15 (d, *J* = 7.9 Hz, 1H), 7.03 (s, 1H), 6.52 (d, *J* = 1.5 Hz, 1H), 6.34 (d, *J* = 7.1 Hz, 1H), 5.03 (d, *J* = 12.5 Hz, 1H), 4.95 (d, *J* = 12.6 Hz, 1H), 2.45–2.38 (m, 1H), 2.33 (s, 3H), 2.29 (s, 1H), 2.22 (s, 4H), 1.98 (d, J = 7.3 Hz, 1H), 1.87 (td, *J* = 14.2, 6.2 Hz, 1H), 1.69 (dd, *J* = 16.0, 8.1 Hz, 1H), 1.59 (d, *J* = 9.4 Hz, 1H), 1.54 (s, 2H), 1.49 (ddd, *J* = 14.7, 5.3, 1.7 Hz, 1H), 1.41 (s, 3H), 1.23 (s, 3H), 1.21 (s, 3H), 1.09 (s, 3H), 0.49 (s, 3H); ^13^C NMR (150 MHz, CDCl_3_) *δ* 178.23, 178.09, 170.96, 165.04, 146.14, **137.18**, 134.72, **133.81**, **130.57**, **129.46**, **128.81**, 127.52, **126.23**, 119.62, 118.32, 117.65, **64.90**, 45.22, 44.40, 43.19, 40.69, 39.59, 38.40, 36.48, 34.85, 33.37, 32.95, 31.70, 30.91, 30.69, 30.08, 29.68, 28.75, 21.72, **19.13**, 18.63, 10.45.

##### 4-nitrobenzyl 3-hydroxy-9β,13α-dimethyl-2-oxo-24,25,26-trinoroleana-1(10),3,5,7-tetraen-29-oate (1d)

Red powder; yield 62%; HR-ESI-MS *m/z*: 586.3195 [M + H]^+^ (calcd for C_36_H_44_NO_6_, 586.3163); ^1^H NMR (600 MHz, CDCl_3_) *δ* 7.08 (dd, *J* = 8.5, 2.5 Hz, 1H), 7.03–6.95 (m, 2H), 6.50 (d, *J* = 1.5 Hz, 1H), 6.34 (d, *J* = 7.2 Hz, 1H), 5.16 (d, *J* = 13.5 Hz, 1H), 4.96 (d, *J* = 13.5 Hz, 1H), 2.46 (d, *J* = 16.0 Hz, 1H), 2.22 (s, 4H), 2.16–2.02 (m, 2H), 1.90 (td, *J* = 14.1, 6.2 Hz, 1H), 1.76 (ddd, *J* = 17.6, 9.9, 4.8 Hz, 3H), 1.71–1.64 (m, 2H), 1.61 (d, *J* = 7.9 Hz, 2H), 1.58–1.54 (m, 1H), 1.51 (ddd, *J* = 14.9, 5.6, 2.1 Hz, 1H), 1.37 (s, 12H), 1.11 (s, 3H), 0.54 (s, 3H); ^13^C NMR (150 MHz, CDCl_3_) *δ* 178.47, 177.94, 169.75, 164.74, **147.84**, 145.60, **143.25**, 134.09, **128.37** (C × 2), 127.61, **124.02** (C × 2), 119.02, 118.36, 117.27, **64.96**, 45.13, 44.37, 42.99, 40.69, 39.55, 38.42, 36.45, 34.83, 33.63, 32.88, 31.71, 30.70, 30.46, 30.28, 29.85, 28.75, 21.75, 18.84, 10.41.

#### General procedures for synthesising compound 2a–2g

Compound **1a** (200 mg, 0.41 mmol) and NaBH_4_ (31 mg, 0.82 mmol, 2 equiv) were dissolved in 8 ml DCM (extra 0.8 ml methanol for solubilization) and stirred at room temperature for 0.5 h. Then DMAP (100 mg, 0.82 mmol), EDCI (313 mg, 1.64 mmol) and cinnamic acid (364 mg, 2.46 mmol, 6 equiv) were added to the solution directly. The mixture was stirred at 70 °C for 12 h. After confirming the progress of the reaction by a TLC, the mixture dissolved in 20 ml water and 12 ml DCM, the DCM layer was washed with water (20 ml × 3) and dried over anhydrous MgSO4. Then purified by normal phase column chromatography (PE/EA =20:1–5:1) to afford compound **2a** as a white solid (100 mg, 50%). Compounds **2b–2g** was prepared according to the procedures same as **2a** with other ligands containing free carboxyl group.

##### Allyl 2,3-dicinnamoyloxy -24-nor-friedela-1,3,5(10),7-tetraen-29-oate (2a)

White powder; yield 50%; HR-ESI-MS *m/z*: 775.4075 [M + Na]^+^ (calcd for C_50_H_56_NaO_6_, 775.3969); ^1^H NMR (600 MHz, CDCl_3_) *δ* 7.84 (t, *J* = 16.1 Hz, 2H), 7.52–7.50 (m, 2H), 7.49–7.47 (m, 2H), 7.11 (s, 1H), 6.60 (dd, *J* = 33.6, 16.0 Hz, 2H), 5.88 (ddt, *J* = 17.2, 10.5, 5.6 Hz, 1H), 5.77 (dd, *J* = 6.3, 2.2 Hz, 1H), 5.21 (dq, *J* = 10.4, 1.3 Hz, 1H), 4.49 (ddt, *J* = 13.5, 5.5, 1.4 Hz, 1H), 4.42 (ddt, *J* = 13.6, 5.7, 1.5 Hz, 1H), 3.37 (dd, *J* = 21.0, 6.0 Hz, 1H), 3.12 (dd, *J* = 21.1, 2.1 Hz, 1H), 2.47 (d, *J* = 16.6 Hz, 1H), 40 (s, 3H), 1.24 (s, 3H), 1.20 (s, 3H), 1.09 (s, 3H), 0.64 (s, 3H); ^13^C NMR (150 MHz, CDCl_3_) *δ* 178.21, 165.08, 164.77, 149.27, 147.64, 147.15, 146.89, 140.96, 138.37, 134.25, 134.19, **132.43**, 131.65, 130.82, 130.73, 129.04 (C × 2), 129.02 (C × 2), 128.48 (C × 2), 128.43 (C × 2), 128.23, **117.98**, 117.15, 117.00, 116.87, 116.69, **65.16**, 44.49, 43.91, 40.61, 37.73, 37.40, 36.97, 34.91, 34.51 (C × 2), 33.08, 31.74, 30.88, 30.72, 30.36, 30.05, 29.04, 28.33, 22.91, 18.55, 12.81.

##### Allyl 2,3-di(4-fluoro)cinnamoyloxy-24-nor-friedela-1,3,5(10),7-tetraen-29-oate (2 b)

White powder; yield 74%; HR-ESI-MS *m/z*: 811.3898 [M + Na]^+^ (calcd for C_50_H_54_NaF_2_O_6_, 811.3781); ^1^H NMR (600 MHz, CDCl_3_) *δ* 7.79 (t, *J* = 15.6 Hz, 2H), 7.52–7.45 (m, 4H), 7.10 (s, 1H), 7.04 (q, *J* = 8.7 Hz, 4H), 6.51 (dd, *J* = 33.2, 16.0 Hz, 2H), 5.88 (ddt, *J* = 17.2, 10.5, 5.6 Hz, 1H), 5.77 (dd, *J* = 6.3, 2.2 Hz, 1H), 5.34–5.29 (m, 2H), 5.20 (dq, *J* = 10.4, 1.3 Hz, 1H), 4.47 (dt, *J* = 5.6, 1.5 Hz, 1H), 4.42 (dt, *J* = 5.6, 1.5 Hz, 1H), 3.36 (dd, *J* = 21.0, 6.0 Hz, 1H), 3.11 (dd, *J* = 21.0, 2.0 Hz, 1H), 2.46 (d, *J* = 15.3 Hz, 1H), 2.12 (s, 3H), 1.39 (s, 3H), 1.23 (s, 3H), 1.19 (s, 3H), 1.09 (s, 3H), 0.63 (s, 3H); ^13^C NMR (150 MHz, CDCl_3_) *δ* 178.10, 164.81, 164.50, 164.16 (d, J = 252.0 Hz), 164.11 (d, *J *=* *252.0 Hz), 149.15, 147.61, 145.65, 145.40, 140.77, 138.20, **132.32,** 131.62, 130.32 (d, *J *=* *8.6 Hz), 130.30 (d, *J *=* *8.6 Hz), 130.26 (d, *J *=* *9.4 Hz, C × 2), 130.20 (d, *J *=* *9.4 Hz, C × 2), 128.10, **117.86,** 117.01, 116.73, 116.61 (d, *J *=* *1.6 Hz), 116.30 (d, *J *=* *1.6 Hz), 116.14 (d, *J *=* *22.2 Hz, C × 2), 116.11 (d, *J *=* *21.9 Hz, C × 2), **65.02**, 44.35, 43.79, 40.50, 37.60, 37.28, 36.84, 34.79, 34.38 (C × 2), 32.97, 31.62, 30.77, 30.60, 30.22, 29.92, 28.92, 28.21, 22.79, 18.44, 12.68.

##### Allyl 2,3-di(4-chloro)cinnamoyloxy-24-nor-friedela-1,3,5(10),7-tetraen-29-oate (2c)

White powder; yield 56%; HR-ESI-MS *m/z*: 843.3161 [M + Na]^+^ (calcd for C_50_H_54_NaCl_2_O_6_, 843.3190)；^1^H NMR (600 MHz, CDCl_3_) *δ* 7.77 (t, *J* = 15.5 Hz, 2H), 7.42 (dd, *J* = 18.0, 8.5 Hz, 4H), 7.33 (d, *J* = 8.7 Hz, 4H), 7.09 (s, 1H), 6.56 (dd, *J* = 33.4, 16.0 Hz, 2H), 5.88 (ddt, *J* = 17.2, 10.5, 5.6 Hz, 1H), 5.76 (dd, *J* = 6.3, 2.1 Hz, 1H), 5.33–5.28 (m, 1H), 5.20 (dq, *J* = 10.5, 1.4 Hz, 1H), 4.48 (ddt, *J* = 13.5, 5.6, 1.5 Hz, 1H), 4.43–4.39 (m, 1H), 3.36 (dd, *J* = 20.9, 6.0 Hz, 1H), 3.11 (dd, *J* = 21.0, 2.0 Hz, 1H), 2.12 (s, 3H), 1.39 (s, 3H), 1.23 (s, 3H), 1.19 (s, 3H), 1.09 (s, 3H), 0.63 (s, 3H); ^13^C NMR (150 MHz, CDCl_3_) *δ* 178.22, 164.80, 164.49, 149.27, 147.78, 145.65, 145.41, 140.84, 138.26, 136.89, 136.80, 132.67, 132.61, **132.43**, 131.79, 129.59 (C × 2), 129.53 (C × 2), 129.37 (C × 2), 129.35 (C × 2), 128.22, **117.98**, 117.52, 117.21, 117.12, 116.83, **65.14**, 44.47, 43.91, 40.62, 37.72, 37.40, 36.96, 34.91, 34.49 (C × 2), 33.09, 31.74, 30.89, 30.72, 30.34, 30.03, 29.03, 28.32, 22.91, 18.56, 12.81.

##### Allyl 2,3-di(4-trifluoromethyl)cinnamoyloxy-24-nor-friedela-1,3,5(10),7-tetraen-29-oate (2d)

White powder; yield 58%; HR-ESI-MS *m/z*: 911.3828 [M + Na]^+^ (calcd for C_52_H_54_NaF_6_O_6_, 911.3717); ^1^H NMR (600 MHz, CDCl_3_) *δ* 7.83 (dd, *J* = 16.0, 14.3 Hz, 2H), 7.60 (s, 4H), 7.58 (d, *J* = 6.7 Hz, 3H), 7.11 (s, 1H), 6.66 (dd, *J* = 33.8, 16.0 Hz, 2H), 5.88 (ddt, *J* = 17.2, 10.5, 5.6 Hz, 1H), 5.77 (dd, *J* = 6.3, 2.2 Hz, 1H), 5.31 (dq, *J* = 17.3, 1.6 Hz, 1H), 5.20 (dq, *J* = 10.5, 1.4 Hz, 1H), 4.48 (ddt, *J* = 13.5, 5.6, 1.5 Hz, 1H), 4.42 (ddt, *J* = 13.5, 5.7, 1.5 Hz, 1H), 3.37 (dd, *J* = 21.0, 6.1 Hz, 1H), 3.12 (dd, *J* = 21.0, 1.9 Hz, 1H), 2.13 (s, 3H), 1.39 (s, 3H), 1.24 (s, 3H), 1.20 (s, 3H), 1.09 (s, 3H), 0.64 (s, 3H); ^13^C NMR (150 MHz, CDCl_3_) *δ* 178.24, 164.47, 164.16, 149.29, 147.96, 145.24, 144.99, 140.71, 138.15, 137.45, 137.39, **132.44,** 132.38 (d, *J *=* *32.8 Hz), 132.31 (d, *J *=* *33.0 Hz), 131.99, 128.55(C × 2), 128.48(C × 2), 128.24, 126.05 (d, *J *=* *3.7 Hz, C × 2), 126.03 (d, *J *=* *3.4 Hz, C × 2), 123.81 (d, *J *=* *272.3 Hz, C × 2), 119.50, 119.19, **117.98,** 117.10, 116.82, **65.15**, 44.47, 43.93, 40.63, 37.72, 37.43, 36.96, 34.91, 34.52, 34.49, 33.10, 31.74, 30.90, 30.72, 30.33, 30.03, 29.04, 28.33, 22.92, 18.57, 12.83.

##### Allyl 2,3-di(4-methyl)cinnamoyloxy-24-nor-friedela-1,3,5(10),7-tetraen-29-oate (2e)

White powder; yield 63%; HR-ESI-MS *m/z*: 803.4449 [M + Na]^+^ (calcd for C_52_H_60_NaO_6_, 803.4282); ^1^H NMR (600 MHz, CDCl_3_) *δ* 7.81 (t, *J* = 15.8 Hz, 2H), 7.39 (dd, *J* = 18.0, 8.2 Hz, 4H), 7.17–7.11 (m, 4H), 7.10 (s, 1H), 6.54 (dd, *J* = 32.9, 15.9 Hz, 2H), 5.88 (ddt, *J* = 17.3, 10.5, 5.6 Hz, 1H), 5.76 (dd, *J* = 6.3, 2.1 Hz, 1H), 5.30 (s, 1H), 5.20 (dq, *J* = 10.5, 1.4 Hz, 1H), 4.49 (ddt, *J* = 13.5, 5.5, 1.5 Hz, 1H), 4.41 (ddt, *J* = 13.5, 5.7, 1.4 Hz, 1H), 3.36 (dd, *J* = 20.9, 6.0 Hz, 1H), 3.11 (dd, *J* = 21.0, 2.0 Hz, 1H), 2.35 (d, *J* = 4.5 Hz, 6H), 2.12 (s, 3H), 1.39 (s, 3H), 1.23 (s, 3H), 1.19 (s, 3H), 1.09 (s, 3H), 0.63 (s, 3H); ^13^C NMR (150 MHz, CDCl_3_) *δ* 178.20, 165.30, 164.98, 149.26, 147.55, 147.13, 146.88, 141.29, 141.19, 141.04, 138.45, **132.44**, 131.57, 131.54, 131.51, 129.75(C × 2), 129.73(C × 2), 128.49(C × 2), 128.44(C × 2), 128.23, **117.99**, 117.16, 116.88, 115.89, 115.58, **65.16**, 44.49, 43.90, 40.61, 37.74, 37.39, 36.98, 34.91, 34.52, 34.49, 33.08, 31.74, 30.88, 30.72, 30.37, 30.06, 29.04, 28.33, 22.91, 21.64, 21.63, 18.55, 12.80.

##### Allyl 2,3-di(4-methoxy)cinnamoyloxy-24-nor-friedela-1,3,5(10),7-tetraen-29-oate (2f)

White powder; yield 65%; HR-ESI-MS *m/z*: 835.4328 [M + Na]^+^ (calcd for C_52_H_60_NaO_8_, 835.4180); ^1^H NMR (600 MHz, CDCl_3_) *δ* 7.79 (t, *J* = 15.6 Hz, 2H), 7.44 (dd, *J* = 18.1, 8.7 Hz, 4H), 7.09 (s, 1H), 6.85 (t, *J* = 8.9 Hz, 4H), 6.46 (dd, *J* = 32.4, 15.9 Hz, 2H), 5.88 (ddt, *J* = 17.2, 10.5, 5.6 Hz, 1H), 5.78–5.75 (m, 1H), 5.30 (s, 2H), 5.20 (dq, *J* = 10.5, 1.3 Hz, 1H), 4.49 (ddt, *J* = 13.5, 5.5, 1.5 Hz, 1H), 4.41 (ddt, *J* = 13.5, 5.7, 1.5 Hz, 1H), 3.82 (d, *J* = 4.0 Hz, 7H), 3.36 (dd, *J* = 21.0, 6.0 Hz, 1H), 3.11 (dd, *J* = 21.0, 2.0 Hz, 1H), 2.12 (s, 3H), 1.39 (s, 4H), 1.23 (s, 3H), 1.19 (s, 3H), 1.08 (s, 3H), 0.63 (s, 3H); ^13^C NMR (150 MHz, CDCl_3_) *δ* 178.20, 165.46, 165.15, 161.80, 161.73, 149.25, 147.48, 146.74, 146.50, 141.11, 138.53, **132.43**, 131.47, 130.21 (C × 2), 130.14 (C × 2), 128.23, 127.05, 127.00, **117.98**, 117.17, 116.89, 114.45 (C × 2), 114.43 (C × 2), 114.43, 114.13, **65.15**, 55.51 (C × 2), 44.49, 43.89, 40.61, 37.73, 37.38, 36.98, 34.91, 34.52, 34.49, 33.07, 31.73, 30.88, 30.71, 30.37, 30.06, 29.04, 28.33, 22.90, 18.55, 12.80.

##### Allyl 2,3-di(3–(2-thienyl))acroloyloxy-24-nor-friedela-1,3,5(10),7-tetraen-29-oate (2 g)

White powder; yield 68%; HR-ESI-MS *m/z*: 787.3215 [M + Na]^+^ (calcd for C_46_H_52_NaO_6_S_2_, 787.3098); ^1^H NMR (600 MHz, CDCl_3_) *δ* 7.93 (dd, *J* = 15.7, 14.9 Hz, 2H), 7.38 (ddt, *J* = 8.7, 5.1, 1.0 Hz, 2H), 7.24–7.22 (m, 1H), 7.08 (s, 1H), 7.03 (ddd, *J* = 8.5, 5.1, 3.6 Hz, 2H), 6.39 (dd, *J* = 29.2, 15.7 Hz, 2H), 5.88 (ddt, *J* = 17.2, 10.5, 5.6 Hz, 1H), 5.76 (dd, *J* = 6.3, 2.2 Hz, 1H), 5.33–5.28 (m, 1H), 5.20 (dq, *J* = 10.4, 1.3 Hz, 1H), 4.48 (ddt, *J* = 13.5, 5.5, 1.4 Hz, 1H), 4.41 (ddt, *J* = 13.5, 5.7, 1.4 Hz, 1H), 3.35 (dd, *J* = 21.0, 6.0 Hz, 1H), 3.10 (dd, *J* = 21.0, 2.0 Hz, 1H), 2.11 (s, 3H), 1.42 (s, 3H), 1.38 (d, *J* = 6.2 Hz, 5H), 1.23 (s, 3H), 1.19 (s, 3H), 1.08 (s, 3H), 0.63 (s, 3H); ^13^C NMR (150 MHz, CDCl_3_) *δ* 178.20, 164.95, 164.65, 149.25, 147.62, 140.97, 139.46, 139.44, 139.41, 139.20, 138.38, **132.43**, 131.79, 131.62 (C × 2), 129.35, 129.24, 128.29, 128.26, 128.22, **117.99**, 117.14, 116.85, 115.63, 115.30, **65.15**, 44.48, 43.90, 40.61, 37.73, 37.39, 36.97, 34.91, 34.51, 34.49, 33.08, 31.74, 30.88, 30.71, 30.36, 30.05, 29.04, 28.32, 22.90, 18.55, 12.79.

#### Synthesis of compound 2h

A mixture of **1a** (200 mg, 0.41 mmol), K_2_CO_3_ (170 mg, 1.23 mmol) and CH_3_I (128 μL, 2.05 mmol, 5 equiv) in DMF (5 ml) was stirred at room temperature for 12 h. After extraction and other regular post-treatment, the residue was purified by normal phase column chromatography (PE/EA= 10:1) to afford compound **2h** as a red solid (120 mg, 60%).

##### Allyl 3-methoxy-9β,13α-dimethyl-2-oxo-24,25,26-trinoroleana-1(10),3,5,7-tetraen-29-oate (2 h)

Red powder; yield 60%; HR-ESI-MS *m/z*: 505.3319 [M + H]^+^ (calcd for C_33_H_45_O_4_, 505.3312); ^1^H NMR (600 MHz, CDCl_3_) *δ* 6.98–6.93 (m, 1H), 6.43 (s, 1H), 6.29 (d, *J* = 7.0 Hz, 1H), 5.85 (d, *J* = 12.2 Hz, 1H), 5.30 (dq, *J* = 17.1, 1.5 Hz, 1H), 5.22 (dq, *J* = 10.5, 1.4 Hz, 1H), 4.42 (d, *J* = 27.4 Hz, 2H), 3.84 (s, 3H), 3.49 (s, 2H), 1.45 (s, 3H), 1.28 (s, 3H), 1.20 (s, 3H), 1.10 (s, 3H), 0.57 (s, 3H); ^13^C NMR (150 MHz, CDCl_3_) *δ* 177.96, 170.05, 162.48, 150.41, 147.21, **132.14**, 127.39, 124.60, 124.11,119.24, 118.26, **118.03**, **65.18**, 60.28, 45.19, 44.42, 42.43, 40.56, 39.37, 38.28, 36.51, 34.88, 33.60, 32.88, 31.73, 30.89, 30.69, 30.00, 29.72, 28.77, 22.07, 18.72, 10.93.

#### General procedures for synthesising compound 3a–3g

Compound **2a** (60 mg) was dissolved in anhydrous THF (3 ml). N-Methylaniline (71 μL, 0.656 mmol) and catalytic equivalent of Pd[P(C_6_H_5_)_3_]_4_ (15 mg, 0.0131 mmol) were added in ice bath and then stirred at room temperature for 1 h. The mixture was concentrated under vacuum. Then purified by normal phase column chromatography (PE/EA = 10:1) to afford the target compound **3a** as a white solid (45 mg, 75%). Compounds **3b*–*3g** was prepared from **2b*–*2g** according to the procedures same as **3a**.

##### 2,3-Dicinnamoyloxy-24-nor-friedela-1,3,5(10),7-tetraen-29-oic acid (3a)

White powder; yield 75%; HR-ESI-MS *m/z*: 735.3817 [M + Na]^+^ (calcd for C_47_H_52_NaO_6_, 735.3656); ^1^H NMR (600 MHz, CDCl_3_) *δ* 7.84 (dd, *J* = 19.3, 16.0 Hz, 2H), 7.50 (ddt, *J* = 18.7, 6.5, 1.7 Hz, 4H), 7.37–7.32 (m, 6H), 7.11 (s, 1H), 6.59 (dd, *J* = 38.7, 16.0 Hz, 2H), 5.77 (dd, *J* = 6.4, 2.1 Hz, 1H), 3.37 (dd, *J* = 20.9, 6.0 Hz, 1H), 3.11 (dd, *J* = 20.9, 2.0 Hz, 1H), 2.13 (s, 3H), 1.23 (s, 3H), 1.16 (s, 3H), 1.08 (s, 3H), 0.73 (s, 3H); ^13^C NMR (150 MHz, CDCl_3_) *δ* 183.36, 165.02, 164.66, 149.55, 147.65, 147.06, 146.84, 140.97, 138.38, 134.29, 134.23, 131.66, 130.79, 130.71, 129.04 (C × 2), 129.01 (C × 2), 128.47 (C × 2), 128.43 (C × 2), 128.16, 117.22, 117.07, 116.76, 116.76, 44.38, 43.91, 40.35, 37.70, 37.40, 36.91, 34.76, 34.55, 34.30, 32.90, 31.72, 30.72, 30.68, 30.26, 29.73, 29.03, 28.32, 23.09, 18.88, 12.83.

##### 2,3-Di(4-fluoro)cinnamoyloxy-24-nor-friedela-1,3,5(10),7-tetraen-29-oic acid (3b)

White powder; yield 88%; HR-ESI-MS *m/z*: 771.3552 [M + Na]^+^ (calcd for C_47_H_50_NaF_2_O_6_, 771.3468); ^1^H NMR (600 MHz, CDCl_3_) *δ* 7.78 (dd, *J* = 19.1, 16.0 Hz, 2H), 7.52–7.45 (m, 4H), 7.10 (s, 1H), 7.04 (q, *J* = 8.8 Hz, 4H), 6.51 (dd, *J* = 38.4, 16.0 Hz, 2H), 5.76 (dd, *J* = 6.3, 2.2 Hz, 1H), 3.36 (dd, *J* = 20.9, 6.1 Hz, 1H), 3.10 (dd, *J* = 21.0, 2.0 Hz, 1H), 2.12 (s, 3H), 1.37 (s, 3H), 1.23 (s, 3H), 1.16 (s, 3H), 1.08 (s, 3H), 0.72 (s, 3H); ^13^C NMR (150 MHz, CDCl_3_) *δ* 183.24, 164.89, 164.53, 164.27 (d, *J *=* *252.0 Hz), 164.22 (d, *J *=* *252.0 Hz), 149.51, 147.72, 145.70, 145.49, 140.91, 138.32, 131.74, 130.50 (d, *J* = 8.8 Hz), 130.48 (d, *J* = 8.8 Hz), 130.38 (d, *J *=* *8.6 Hz, C × 2), 130.32 (d, *J *=* *8.6 Hz, C × 2), 128.17, 117.21, 116.79 (d, *J *=* *1.6 Hz), 116.73, 116.48 (d, *J *=* *1.6 Hz), 116.26 (d, *J *=* *22.2 Hz, C × 2), 116.22 (d, *J *=* *21.9 Hz, C × 2), 44.39, 43.91, 40.34, 37.69, 37.40, 36.90, 34.76, 34.56, 34.31, 32.89, 31.72, 30.72, 30.67, 30.27, 29.74, 29.02, 28.31, 23.09, 18.89, 12.82.

##### 2,3-Di(4-chloro)cinnamoyloxy-24-nor-friedela-1,3,5(10),7-tetraen-29-oic acid (3c)

White powder; yield 75%; HR-ESI-MS *m/z*: 803.2903 [M + Na]^+^ (calcd for C_47_H_50_NaCl_2_O_6_, 803.2877); ^1^H NMR (600 MHz, CDCl_3_) *δ* 7.76 (dd, *J* = 19.0, 16.0 Hz, 2H), 7.42 (dd, *J* = 18.5, 8.5 Hz, 4H), 7.36–7.30 (m, 5H), 7.26 (s, 2H), 7.10–7.07 (m, 2H), 6.55 (dd, *J* = 38.8, 16.0 Hz, 2H), 5.77 (dd, *J* = 6.3, 2.2 Hz, 1H), 3.37 (dd, *J* = 20.9, 6.1 Hz, 1H), 3.10 (dd, *J* = 20.9, 2.0 Hz, 1H), 2.12 (s, 3H), 1.43 (s, 3H), 1.34 (s, 3H), 1.26 (s, 6H), 1.23 (s, 3H), 1.17 (s, 3H), 1.08 (s, 3H), 0.72 (s, 3H); ^13^C NMR (150 MHz, CDCl_3_) *δ* 182.65, 164.78, 164.42, 149.49, 147.77, 145.60, 145.38, 140.85, 138.27, 136.87, 136.78, 132.70, 132.64, 131.81, 129.59(C × 2), 129.54(C × 2), 129.38(C × 2), 129.35(C × 2), 128.17, 117.57, 117.27, 117.20, 116.73, 44.39, 43.91, 40.33, 37.70, 37.40, 36.90, 35.13, 34.59, 34.31, 32.89, 31.65, 30.67, 30.47, 30.28, 29.85, 29.02, 28.31, 23.08, 18.89, 12.83.

##### 2,3-Di(4-trifluoromethyl)cinnamoyloxy-24-nor-friedela-1,3,5(10),7-tetraen-29-oic acid (3d)

White powder; yield 90%; HR-ESI-MS *m/z*: 871.3434 [M + Na]^+^ (calcd for C_49_H_50_NaF_6_O_6_, 871.3404); ^1^H NMR (600 MHz, CDCl_3_) *δ* 7.83 (dd, *J* = 18.8, 16.0 Hz, 2H), 7.60 (s, 4H), 7.59–7.55 (m, 4H), 7.11 (s, 1H), 6.66 (dd, *J* = 39.5, 16.1 Hz, 2H), 5.77 (dd, *J* = 6.4, 2.2 Hz, 1H), 3.37 (dd, *J* = 20.9, 6.1 Hz, 1H), 3.11 (dd, *J* = 20.8, 2.0 Hz, 1H), 2.41 (d, *J* = 15.8 Hz, 1H), 2.13 (s, 4H), 1.38 (s, 4H), 1.23 (s, 3H), 1.17 (s, 3H), 1.08 (s, 3H), 0.72 (s, 3H); ^13^C NMR (150 MHz, CDCl_3_) *δ* 183.07, 164.43, 164.07, 149.50, 147.95, 145.18, 144.96, 140.72, 138.16, 137.48, 137.42, 132.37 (d, *J *=* *32.5 Hz), 132.29 (d, *J *=* *32.6 Hz), 131.99, 126.04 (d, *J *=* *3.8 Hz, C × 2), 126.02 (d, *J *=* *3.5 Hz, C × 2), 123.82 (d, *J *=* *272.3 Hz, C × 2), 119.54, 119.23, 117.18, 116.70, 44.38, 43.92, 40.34, 37.68, 37.42, 36.88, 34.74, 34.57, 34.30, 32.88, 31.71, 30.73, 30.67, 30.26, 29.75, 29.01, 28.31, 23.08, 18.91, 12.84.

##### 2,3-Di(4-methyl)cinnamoyloxy-24-nor-friedela-1,3,5(10),7-tetraen-29-oic acid (3e)

White powder; yield 73%; HR-ESI-MS *m/z*: 763.3958 [M + Na]^+^ (calcd for C_49_H_56_NaO_6_, 763.3969); ^1^H NMR (600 MHz, CDCl_3_) *δ* 7.80 (dd, *J* = 19.1, 16.0 Hz, 2H), 7.39 (dd, *J* = 17.9, 8.1 Hz, 5H), 7.17–7.13 (m, 4H), 7.11–7.07 (m, 2H), 6.54 (dd, *J* = 38.0, 16.0 Hz, 2H), 5.77 (dd, *J* = 6.4, 2.2 Hz, 1H), 3.37 (dd, *J* = 20.9, 6.1 Hz, 1H), 3.10 (dd, *J* = 20.9, 2.1 Hz, 1H), 2.12 (s, 3H), 1.34 (s, 3H), 1.23 (s, 3H), 1.17 (s, 3H), 1.08 (s, 3H), 0.72 (s, 3H); ^13^C NMR (150 MHz, CDCl_3_) *δ* 182.83, 165.25, 164.89, 149.52, 147.55, 147.04, 146.84, 141.26, 141.16, 141.04, 138.45, 131.60, 131.57, 131.54, 129.75(C × 2), 129.72(C × 2), 128.48(C × 2), 128.44(C × 2), 128.16, 117.23, 116.77, 115.94, 115.64, 44.39, 43.91, 40.32, 37.70, 37.39, 36.92, 35.12, 34.59, 34.31, 32.90, 31.72, 30.68, 30.47, 30.34, 29.85, 29.03, 28.32, 23.08, 21.64, 21.63, 18.88, 12.82.

##### 2,3-Di(4-methoxy)cinnamoyloxy-24-nor-friedela-1,3,5(10),7-tetraen-29-oic acid (3f)

White powder; yield 70%; HR-ESI-MS *m/z*: 795.3917 [M + Na]^+^ (calcd for C_49_H_56_NaO_8_, 795.3867); ^1^H NMR (600 MHz, CDCl_3_) *δ* 7.78 (dd, *J* = 18.8, 15.9 Hz, 2H), 7.54 (dd, *J* = 8.6, 0.7 Hz, 1H), 7.44 (dd, *J* = 18.3, 8.8 Hz, 4H), 7.36 (q, *J* = 2.3 Hz, 1H), 7.13 (dd, *J* = 8.6, 2.5 Hz, 1H), 7.10 (s, 1H), 6.85 (t, *J* = 9.0 Hz, 4H), 6.45 (dd, *J* = 37.4, 15.9 Hz, 2H), 5.76 (dd, *J* = 6.4, 2.2 Hz, 1H), 4.12 (q, *J* = 7.1 Hz, 1H), 3.82 (d, *J* = 4.4 Hz, 6H), 3.36 (dd, *J* = 20.8, 6.1 Hz, 1H), 3.10 (dd, *J* = 20.8, 2.0 Hz, 1H), 2.12 (s, 3H), 1.42 (s, 3H), 1.33 (s, 6H), 1.23 (s, 3H), 1.18 (s, 3H), 1.08 (s, 3H), 0.73 (s, 3H); ^13^C NMR (150 MHz, CDCl_3_) *δ* 182.95, 165.43, 165.07, 161.79, 161.71, 149.48, 147.48, 146.67, 146.47, 141.12, 138.53, 131.49, 130.20 (C × 2), 130.15 (C × 2), 128.18, 127.09, 127.03, 117.24, 116.80, 114.49, 114.45 (C × 2), 114.43 (C × 2), 114.19, 55.52 (C × 2), 44.40, 43.90, 40.32, 37.71, 37.38, 36.93, 35.01, 34.67, 34.34, 32.89, 31.71, 30.67, 30.46, 30.33, 29.84, 29.02, 28.32, 23.07, 18.86, 12.82.

##### 2,3-Di(3–(2-thienyl))acroloyloxy-24-nor-friedela-1,3,5(10),7-tetraen-29-oic acid (3 g)

White powder; yield 91%; HR-ESI-MS *m/z*: 747.2775 [M + Na]^+^ (calcd for C_43_H_48_NaO_6_S_2_, 747.2785); ^1^H NMR (600 MHz, CDCl_3_) *δ* 7.92 (dd, *J* = 19.7, 15.7 Hz, 2H), 7.66 (ddt, *J* = 12.2, 7.0, 1.3 Hz, 1H), 7.55–7.49 (m, 1H), 7.43 (td, *J* = 8.0, 3.1 Hz, 1H), 7.39–7.34 (m, 3H), 7.24–7.22 (m, 1H), 7.03 (ddd, *J* = 7.2, 5.1, 3.6 Hz, 2H), 6.38 (dd, *J* = 33.7, 15.7 Hz, 2H), 5.76 (dd, *J* = 6.4, 2.2 Hz, 1H), 3.36 (dd, *J* = 20.9, 6.0 Hz, 1H), 3.10 (dd, *J* = 20.9, 2.1 Hz, 1H), 2.11 (s, 3H), 1.42 (s, 3H), 1.23 (s, 3H), 1.17 (s, 3H), 0.71 (s, 3H); ^13^C NMR (151 MHz, CDCl_3_) *δ* 182.99, 164.90, 164.55, 149.49, 147.62, 140.97, 139.48, 139.43, 139.36, 139.15, 138.37, 131.74, 131.63, 131.58, 129.31, 129.21, 128.28, 128.25, 128.16, 117.18, 116.75, 115.68, 115.36, 44.39, 43.90, 40.32, 37.69, 37.38, 36.91, 34.78, 34.54, 34.33, 32.89, 31.65, 30.67, 30.33, 30.28, 29.84, 29.02, 28.31, 23.08, 18.85, 12.81.

#### General procedures for synthesising compound 3h and 3i

Celastrol (100 mg, 0.22 mmol) and NaBH_4_ (42 mg, 1.11 mmol, 5 equiv) were dissolved in 4 ml DCM (extra 0.4 ml methanol for solubilization) and stirred at room temperature for 0.5 h. Then DMAP (27 mg, 0.22 mmol), triethylamine (91 μL, 0.66 mmol) and acetic anhydride (83 μL, 0.88 mmol, 4 equiv) were added to the solution directly. The mixture was stirred at 48 °C for 12 h. After confirming the progress of the reaction by a TLC, the mixture dissolved in 10 ml water and 6 ml DCM, the DCM layer was washed with water (10 ml × 3) and dried over anhydrous MgSO4. Then purified by normal phase column chromatography (PE/EA =12:1) to afford compound **3h** as a white solid (60 mg, 60%). Compound **3i** was similarly synthesised just by replacing acetic anhydride with acrylic anhydride (101 μL, 0.88 mmol).

##### 2,3-Diacryloyloxy-24-nor-friedela-1,3,5(10),7-tetraen-29-oic acid (3h)

White powder; yield 60%; HR-ESI-MS *m/z*: 559.3197 [M + Na]^+^ (calcd for C_33_H_44_NaO_6_, 559.3030); ^1^H NMR (600 MHz, CDCl_3_) *δ* 6.99 (s, 1H), 5.73 (dd, *J* = 6.3, 2.2 Hz, 1H), 3.32 (dd, *J* = 20.9, 6.0 Hz, 1H), 3.06 (d, *J* = 20.9 Hz, 1H), 2.39 (d, *J* = 15.6 Hz, 1H), 2.30 (s, 3H), 2.27 (s, 3H), 2.06 (s, 3H), 1.33 (s, 3H), 1.29 (s, 3H), 1.21 (s, 3H), 1.13 (s, 3H), 1.05 (s, 3H), 0.68 (s, 3H); ^13^C NMR (150 MHz, CDCl_3_) *δ* 183.74, 168.85, 168.51, 149.46, 147.66, 140.71, 138.17, 131.66, 127.93, 117.13, 116.71, 44.34, 43.88, 40.34, 37.65, 37.34, 36.85, 34.72, 34.52, 34.29, 32.82, 31.65, 30.63, 30.28, 30.23, 29.68, 28.99, 28.25, 23.07, 20.85, 20.58, 18.85, 12.73.

##### 2,3-Diacetoxy-24-nor-friedela-1,3,5(10),7-tetraen-29-oic acid (3i)

White powder; yield 57%; HR-ESI-MS *m/z*: 583.3015 [M + Na]^+^ (calcd for C_35_H_44_NaO_6_, 583.3030); ^1^H NMR (600 MHz, CDCl_3_) *δ* 7.06 (s, 1H), 6.56 (ddd, *J* = 20.0, 17.4, 1.3 Hz, 2H), 6.27 (dd, *J* = 10.5, 7.6 Hz, 1H), 5.99 (dd, *J* = 10.4, 1.2 Hz, 1H), 5.96 (dd, *J* = 10.5, 1.2 Hz, 1H), 5.75 (dd, *J* = 6.3, 2.2 Hz, 1H), 3.34 (dd, *J* = 20.9, 6.1 Hz, 1H), 3.08 (dd, *J* = 20.9, 2.1 Hz, 1H), 2.72 (t, *J* = 6.3 Hz, 1H), 2.07 (s, 3H), 1.35 (s, 3H), 1.33 (s, 3H), 1.28 (s, 3H), 1.22 (s, 3H), 1.18 (s, 3H), 1.07 (s, 3H), 0.71 (s, 3H); ^13^C NMR (150 MHz, CDCl_3_) *δ* 183.97, 164.05, 163.68, 149.41, 147.75, 140.59, 138.00, 133.03, 132.80, 131.75, 128.20, 128.11, 127.62, 117.15, 116.69, 44.38, 43.90, 40.37, 37.69, 37.38, 36.90, 34.74, 34.54, 34.33, 32.85, 31.68, 30.66, 30.33, 30.28, 29.72, 28.98, 28.27, 23.03, 18.81, 12.70.

#### General procedures for synthesising compound 4a–4n

A mixture of celastrol (100 mg, 0.22 mmol), BOP (118 mg, 0.27 mmol), DIEA (118 μL, 0.66 mmol) and ethylamine (40 μL, 0.66 mmol, 3 equiv) in DCM (3 ml) was stirred at room temperature overnight. Then the mixture was concentrated and purified by normal phase column chromatography (DCM/MeOH = 30:1) to afford the target compound **4a** as a red solid (82 mg, 82%). The following compounds **4 b-4n** were prepared according to the procedures same as **4a**.

##### 3-Hydroxy-9β,13α-dimethyl-2-oxo-24,25,26-trinoroleana-1(10),3,5,7-tetraen-29-oic acid N-ethylamide (4a)

Red powder; yield 82%; HR-ESI-MS *m/z*: 478.3381 [M + H]^+^ (calcd for C_31_H_44_NO_3_, 478.3316); ^1^H NMR (600 MHz, CDCl_3_) *δ* 7.02 (dd, *J* = 7.1, 1.4 Hz, 1H), 6.53 (d, *J* = 1.4 Hz, 1H), 6.34 (d, *J* = 7.1 Hz, 1H), 5.64 (t, *J* = 5.4 Hz, 1H), 5.30 (s, 1H), 3.16 (qd, *J* = 7.2, 5.2 Hz, 3H), 2.45 (d, *J* = 13.7 Hz, 1H), 2.21 (s, 3H), 1.43 (s, 3H), 1.26 (s, 4H), 1.14 (s, 3H), 1.12 (s, 3H), 1.07 (d, *J* = 9.2 Hz, 3H), 0.64 (s, 3H); ^13^C NMR (150 MHz, CDCl_3_) *δ* 178.38, 177.72, 170.71, 164.94, 146.14, 134.36, 127.54, 119.63, 118.18, 117.32, 45.22, 44.51, 43.21, 40.31, 39.51, 38.32, 36.50, 35.15, **34.62**, 33.94, 33.61, 31.74, 31.25, 31.01, 30.29, 29.59, 28.82, 21.89, 18.47, **14.76**, 10.41.

##### 3-Hydroxy-9β,13α-dimethyl-2-oxo-24,25,26-trinoroleana-1(10),3,5,7-tetraen-29-oic acid 2-methoxyethylamide (4b)

Red powder; yield 70%; HR-ESI-MS *m/z*: 508.3462 [M + H]^+^ (calcd for C_32_H_46_NO_4_, 508.3421); ^1^H NMR (600 MHz, CDCl_3_) *δ* 7.02 (dd, *J* = 7.1, 1.4 Hz, 1H), 6.75 (s, 1H), 6.52 (d, *J* = 1.5 Hz, 1H), 6.34 (q, *J* = 8.8, 7.6 Hz, 2H), 6.20 (d, *J* = 9.9 Hz, 1H), 6.11 (t, *J* = 5.1 Hz, 1H), 5.46 (d, *J* = 6.4 Hz, 1H), 2.21 (s, 3H), 1.25 (s, 3H), 1.21 (s, 3H), 1.15 (s, 3H), 1.12 (s, 3H), 1.08 (s, 3H), 1.02 (s, 3H), 0.63 (s, 3H); ^13^C NMR (150 MHz, CDCl_3_) *δ* 179.76, 178.06, 170.68, 164.99, 146.15, 134.41, 127.52, 119.59, 118.20, 117.35, **71.01**, **58.87**, 45.23, 44.52, 43.20, 40.43, 39.38, 38.32, 36.48, 35.07, **34.44**, 33.93, 33.61, 31.75, 31.32, 30.96, 30.20, 29.58, 28.81, 21.87, 18.41, 10.41.

##### 3-Hydroxy-9β,13α-dimethyl-2-oxo-24,25,26-trinoroleana-1(10),3,5,7-tetraen-29-oic acid 2-dimethylaminoethylamide (4c)

Red powder; yield 53%; HR-ESI-MS *m/z*: 521.3906 [M + H]^+^ (calcd for C_33_H_49_N_2_O_3_, 521.3738); ^1^H NMR (600 MHz, CDCl_3_) *δ* 7.02 (d, *J* = 5.7 Hz, 1H), 6.82 (s, 1H), 6.50 (d, *J* = 1.5 Hz, 1H), 6.35 (d, *J* = 7.2 Hz, 1H), 3.28 (s, 2H), 2.47 (s, 6H), 2.21 (s, 3H), 1.44 (s, 3H), 1.26 (s, 3H), 1.16 (s, 3H), 1.11 (s, 3H), 0.61 (s, 3H); ^13^C NMR (150 MHz, CDCl_3_) *δ* 180.49, 178.50, 170.42, 165.03, 146.19, 134.43, 127.54, 119.59, 118.29, 117.44, **58.81**, 45.21, **44.68**(C × 2), 44.51, 43.16, 40.49, 39.53, 38.34, **36.84**, 36.44, 34.95, 33.92, 33.67, 31.75, 31.26, 30.87, 29.96, 29.56, 28.79, 21.88, 18.45, 10.42.

##### 3-Hydroxy-9β,13α-dimethyl-2-oxo-24,25,26-trinoroleana-1(10),3,5,7-tetraen-29-oic acid (R/S)-tetrahydrofurfurylamide (4d)

Red powder; yield 58%; HR-ESI-MS *m/z*: 534.3638 [M + H]^+^ (calcd for C_34_H_48_NO_4_, 534.3578); ^1^H NMR (600 MHz, CDCl_3_) *δ* 7.03 (dd, *J* = 7.2, 1.4 Hz, 1H), 6.54 (d, *J* = 1.4 Hz, 1H), 6.35 (d, *J* = 7.1 Hz, 2H), 3.86–3.80 (m, 2H), 3.75–3.69 (m, 2H), 2.96 (dtd, *J* = 13.8, 8.1, 4.2 Hz, 1H), 2.21 (s, 3H), 1.43 (s, 3H), 1.16 (s, 3H), 1.11 (s, 3H), 1.02 (s, 3H), 0.63 (s, 3H); ^13^C NMR (150 MHz, CDCl_3_) *δ* 179.77/179.66 (*R/S*), 178.11/178.00, 171.02, 165.04, 146.15, 134.55, 127.50, 119.56, 118.22, 117.45, **77.68/77.59**, **68.21/68.24)**, 45.27, 44.50/44.54, **43.80,** 43.27, 40.50/40.39, 39.52/39.57, 38.31, 36.46, 35.09, 34.01/33.93, 33.60, 31.75, 31.22, 30.98/30.95, **30.75**, 30.29, 29.55/29.57, 28.83, **26.03**, 21.88, 18.43/18.46, 10.42. (*R/S,* optical antipodes).

##### 3-Hydroxy-9β,13α-dimethyl-2-oxo-24,25,26-trinoroleana-1(10),3,5,7-tetraen-29-oic acid 2-morpholinylethylamide (4e)

Red powder; yield 65%; HR-ESI-MS *m/z*: 563.4022 [M + H]^+^ (calcd for C_35_H_51_N_2_O_4_, 563.3843); ^1^H NMR (600 MHz, CDCl_3_) *δ* 6.99 (d, *J* = 5.6 Hz, 2H), 6.50 (d, *J* = 1.4 Hz, 1H), 6.32 (d, *J* = 7.2 Hz, 1H), 3.71 (s, 4H), 3.20 (d, *J* = 5.5 Hz, 2H), 2.64 (d, *J* = 9.4 Hz, 6H), 2.20 (s, 3H), 1.43 (s, 3H), 1.25 (s, 3H), 1.14 (d, *J* = 15.3 Hz, 6H), 0.61 (s, 3H); ^13^C NMR (150 MHz, CDCl_3_) *δ* 178.47, 178.03, 170.18, 164.81, 146.16, 134.04, 127.57, 119.68, 118.12, 117.15, **66.99**, **56.47**, **53.27**, 45.17, 44.47, 43.10, 40.42, 39.45, 38.31, **37.00**, **36.97**, 36.50, **35.45**, 35.08, 33.87, 33.59, 31.76, 31.25, 30.98, 30.25, 29.50, 28.81, 21.88, 18.40, 10.39.

##### 3-Hydroxy-9β,13α-dimethyl-2-oxo-24,25,26-trinoroleana-1(10),3,5,7-tetraen-29-oic acid (R/S)-2–(1-methylpyrrolidin-2-yl)ethan-1-amide (4f)

Red powder; yield 53%; HR-ESI-MS *m/z*: 561.4238 [M + H]^+^ (calcd for C_36_H_53_N_2_O_3_, 561.4051); ^1^H NMR (600 MHz, CDCl_3_) *δ* 7.09 (d, *J* = 7.1 Hz, 1H), 6.50 (d, *J* = 1.3 Hz, 1H), 6.37 (d, *J* = 5.4 Hz, 1H), 3.70–3.64 (m, 1H), 3.22–3.16 (m, 3H), 2.21 (s, 4H), 2.16 (q, *J* = 6.6, 5.4 Hz, 2H), 1.43 (s, 3H), 1.25 (s, 6H), 1.14 (d, *J* = 3.1 Hz, 3H), 1.10 (s, 4H), 0.53 (d, *J* = 6.8 Hz, 3H). ^13^C NMR (150 MHz, CDCl_3_) *δ*181.17/181.47 (*R/S*), 178.32, 171.68, 165.55/165.58, 146.20, 135.48, 127.40, 119.34/119.30, 118.48/118.51, 118.19/118.21, **68.10/67.72**, **56.64/56.61**, 45.31, 44.43, 43.38, **40.59/40.53**, 40.41, 39.55/39.52, 38.42/38.44, 36.33, 35.39, 33.71, 33.69, **33.58**, 31.67, 31.17, 30.82, **30.02/29.98**, 29.84, 29.49, **29.11**, 28.77, **26.54**, 21.77/21.73, 18.50, 10.47.

##### 3-Hydroxy-9β,13α-dimethyl-2-oxo-24,25,26-trinoroleana-1(10),3,5,7-tetraen-29-oic acid 2–(4-methylpiperazinyl) ethylamide (4g)

Red powder; yield 63%; HR-ESI-MS *m/z*: 576.4317 [M + H]^+^ (calcd for C_36_H_54_N_3_O_3_, 576.4160); ^1^H NMR (600 MHz, CDCl_3_) *δ* 7.00 (dd, *J* = 7.1, 1.4 Hz, 1H), 6.51 (d, *J* = 1.4 Hz, 1H), 6.33 (d, *J* = 7.1 Hz, 1H), 3.19 (q, *J* = 5.6 Hz, 2H), 2.33 (s, 3H), 2.20 (s, 3H), 1.44 (s, 3H), 1.25 (d, *J* = 4.6 Hz, 4H), 1.14 (d, *J* = 13.7 Hz, 6H), 1.04 (dd, *J* = 14.1, 3.7 Hz, 1H), 0.62 (s, 3H); ^13^C NMR (150 MHz, CDCl_3_) *δ* 178.48, 178.27, 170.28, 164.86, 146.17, 134.15, 127.57, 119.67, 118.16, 117.22, **56.00**, **55.16** (C × 2), **52.56** (C × 2), **46.03**, 45.19, 44.50, 43.13, 40.43, 39.47, 38.32, 36.53, **35.85**, 35.13, 33.87, 33.62, 31.78, 31.27, 30.96, 30.22, 29.52, 28.82, 21.89, 18.41, 10.41.

##### 3-Hydroxy-9β,13α-dimethyl-2-oxo-24,25,26-trinoroleana-1(10),3,5,7-tetraen-29-oic acid phenethylamide (4h)

Red powder; yield 67%; HR-ESI-MS *m/z*: 554.3686 [M + H]^+^ (calcd for C_37_H_48_NO_3_, 554.3629); ^1^H NMR (600 MHz, CDCl_3_) *δ* 7.22 (d, *J* = 7.4 Hz, 1H), 7.14 (d, *J* = 6.9 Hz, 2H), 7.01 (dd, *J* = 7.1, 1.4 Hz, 1H), 6.98 (s, 1H), 6.53 (d, *J* = 1.4 Hz, 1H), 6.32 (s, 1H), 5.66 (t, *J* = 5.3 Hz, 1H), 3.37 (q, *J* = 6.8 Hz, 2H), 2.74 (t, *J* = 6.9 Hz, 2H), 2.38 (d, *J* = 15.3 Hz, 1H), 2.21 (s, 3H), 1.63 (s, 4H), 1.43 (s, 4H), 1.23 (s, 3H), 1.09 (d, *J* = 8.8 Hz, 6H), 0.53 (s, 3H); ^13^C NMR (150 MHz, CDCl_3_) *δ* 178.50, 177.79, 170.34, 164.89, 146.15, **139.01**, 134.06, **128.85** (C × 2), **128.83** (C × 2), 127.56, **126.73**, 119.72, 118.10, 117.11, 45.14, 44.44, 43.13, **40.76**, 40.45, 39.43, 38.32, 36.48, 35.33, **35.01**, 33.85, 33.64, 31.71, 31.18, 30.90, 30.25, 29.53, 28.76, 21.87, 18.42, 10.41.

##### 3-Hydroxy-9β,13α-dimethyl-2-oxo-24,25,26-trinoroleana-1(10),3,5,7-tetraen-29-oic acid 4-methoxyphenethylamide (4i)

Red powder; yield 65%; HR-ESI-MS *m/z*: 584.3725 [M + H]^+^ (calcd for C_38_H_50_NO_4_, 584.3734); ^1^H NMR (600 MHz, CDCl_3_) *δ* 7.05 (d, *J* = 8.5 Hz, 2H), 7.01 (dd, *J* = 7.1, 1.4 Hz, 1H), 6.82 (d, *J* = 8.6 Hz, 2H), 6.53 (d, *J* = 1.4 Hz, 1H), 6.33 (d, *J* = 7.2 Hz, 1H), 5.66 (t, *J* = 5.3 Hz, 1H), 3.79 (s, 4H), 3.35–3.30 (m, 2H), 2.68 (t, *J* = 6.9 Hz, 2H), 2.21 (s, 3H), 2.10 (ddd, *J* = 13.6, 4.8, 2.1 Hz, 1H), 1.42 (s, 4H), 1.23 (s, 3H), 1.09 (d, *J* = 9.1 Hz, 6H), 0.52 (s, 3H); ^13^C NMR (150 MHz, CDCl_3_) *δ* 178.43, 177.79, 170.48, 164.91, **158.45**, 146.14, 134.19, **130.95**, **129.79** (C × 2), 127.54, 119.67, 118.12, 117.19, **114.23** (C × 2), **55.42**, 45.15, 44.44, 43.16, **40.96**, 40.44, 39.45, 38.30, 36.51, 35.03, **34.38**, 33.84, 33.62, 31.71, 31.17, 30.89, 30.27, 29.53, 28.74, 21.85, 18.41, 10.41.

##### 3-Hydroxy-9β,13α-dimethyl-2-oxo-24,25,26-trinoroleana-1(10),3,5,7-tetraen-29-oic acid 4-fluoro-2-phenethylamide (4j)

Red powder; yield 70%; HR-ESI-MS *m/z*: 572.3566 [M + H]^+^ (calcd for C_37_H_47_FNO_3_, 572.3534); ^1^H NMR (600 MHz, CDCl_3_) *δ* 7.11–7.08 (m, 2H), 7.03 (dd, *J* = 7.1, 1.4 Hz, 1H), 6.95 (s, 2H), 6.54 (d, *J* = 1.4 Hz, 1H), 6.34 (d, *J* = 7.1 Hz, 1H), 5.67 (t, *J* = 5.4 Hz, 1H), 3.34 (d, *J* = 6.7 Hz, 3H), 2.72 (t, *J* = 7.0 Hz, 2H), 2.40 (d, *J* = 15.2 Hz, 1H), 2.22 (s, 3H), 2.11 (ddd, *J* = 13.4, 4.8, 2.0 Hz, 1H), 1.43 (s, 3H), 1.23 (s, 3H), 1.10 (s, 3H), 1.08 (s, 3H), 0.50 (s, 3H); ^13^C NMR (150 MHz, CDCl_3_) *δ* 178.32, 177.93, 170.71, 164.96, **161.79** (d, *J *=* *244.3 Hz), 146.14, **134.67** (d, *J *=* *3.2 Hz), 134.41, **130.28** (d, *J *=* *7.7 Hz, C × 2), 127.54, 119.64, 118.20, 117.38, **115.61**(d, J = 21.5 Hz, C × 2), 45.17, 44.42, 43.21, **40.96**, 40.56, 39.48, 38.33, 36.48, 35.01, **34.57**, 33.86, 33.65, 31.70, 31.16, 30.92, 30.28, 29.56, 28.73, 21.82, 18.41, 10.43.

##### 3-Hydroxy-9β,13α-dimethyl-2-oxo-24,25,26-trinoroleana-1(10),3,5,7-tetraen-29-oic acid 2–(4-trifluoromethyl-phenyl)-ethylamide (4k)

Red powder; yield 58%; HR-ESI-MS *m/z*: 622.3483 [M + H]^+^ (calcd for C_38_H_47_F_3_NO_3_, 622.3503); ^1^H NMR (600 Hz, CDCl_3_) *δ* 7.53 (d, *J* = 7.9 Hz, 2H), 7.02 (dd, *J* = 7.1, 1.4 Hz, 1H), 6.53 (d, *J* = 1.4 Hz, 1H), 6.33 (d, *J* = 7.1 Hz, 1H), 5.71 (t, *J* = 5.5 Hz, 1H), 3.44–3.33 (m, 3H), 2.21 (s, 3H), 1.88–1.76 (m, 6H), 1.58–1.53 (m, 4H), 1.43 (s, 3H), 1.23 (s, 3H), 1.11 (s, 3H), 1.09 (s, 3H), 0.52 (s, 3H). ^13^C NMR (150 MHz, CDCl_3_) *δ* 178.36, 178.05, 170.55, 164.93, 146.14, **143.21**, 134.35, **129.26** (d, J = 4.0 Hz), **129.25** (C × 2), 127.56, **125.69** (d, J = 3.5 Hz, C × 2), **122.94** (dd, J = 292.2, 129.3 Hz), 119.63, 118.20, 117.35, 45.16, 44.39, 43.19, **40.69**, 40.51, 39.45, 38.32, 36.43, **35.26**, 35.00, 33.83, 33.60, 31.69, 31.10, 30.91, 30.29, 29.55, 28.73, 21.84, 18.49, 10.41.

##### 3-Hydroxy-9β,13α-dimethyl-2-oxo-24,25,26-trinoroleana-1(10),3,5,7-tetraen-29-oic acid 2-(pyrrol-1-yl) ethanamide (4l)

Red powder; yield 63%; HR-ESI-MS *m/z*: 543.3607 [M + H]^+^ (calcd for C_35_H_47_N_2_O_3_, 543.3581); ^1^H NMR (600 MHz, MeOD) δ 7.21 (dd, *J* = 7.1, 1.4 Hz, 1H), 6.62 (t, *J* = 2.1 Hz, 3H), 6.46 (d, *J* = 1.4 Hz, 2H), 6.00 (t, *J* = 2.1 Hz, 3H), 5.49 (s, 1H), 3.95 (d, *J* = 6.3 Hz, 3H), 2.43 (d, *J* = 16.5 Hz, 1H), 2.22 (d, *J* = 12.7 Hz, 4H), 2.13 (s, 1H), 2.01 (ddd, *J* = 14.2, 7.8, 3.9 Hz, 3H), 1.50 (s, 3H), 1.45 (s, 3H), 1.29 (s, 3H), 0.58 (d, *J* = 13.7 Hz, 3H); ^13^C NMR (150 MHz, MeOD) δ 181.27, 180.10, 172.25, 166.48, 147.84, 136.38, 128.60, **121.70** (C × 2), 120.51, 120.07, 119.70, **109.08** (C × 2), **49.57**, 46.29, 45.82, 44.23, **42.48**, 41.44, 40.62, 38.87, 37.57, 36.11, 34.69, 34.07, 32.05, 32.02, 31.72, 30.69, 30.47, 29.75, 22.20, 19.17, 10.30.

##### 3-Hydroxy-9β,13α-dimethyl-2-oxo-24,25,26-trinoroleana-1(10),3,5,7-tetraen-29-oic acid 2–(2-thienyl) ethylamide (4m)

Red powder; yield 55%; HR-ESI-MS *m/z*: 560.3201 [M + H]^+^ (calcd for C_35_H_46_NO_3_S, 560.3193); ^1^H NMR (600 MHz, CDCl_3_) δ 7.15 (dd, *J* = 5.1, 1.2 Hz, 1H), 7.02 (dd, *J* = 7.1, 1.4 Hz, 1H), 6.93 (dd, *J* = 5.1, 3.4 Hz, 1H), 6.79 (dd, *J* = 3.5, 1.1 Hz, 1H), 6.54 (d, *J* = 1.3 Hz, 1H), 6.34 (d, *J* = 7.2 Hz, 1H), 5.86 (t, *J* = 5.6 Hz, 1H), 3.39 (d, *J* = 4.7 Hz, 2H), 3.00–2.94 (m, 2H), 2.44–2.39 (m, 1H), 2.21 (s, 3H), 2.11 (ddd, *J* = 13.4, 4.8, 2.0 Hz, 1H), 1.48 (ddd, *J* = 14.8, 5.7, 2.0 Hz, 1H), 1.43 (s, 3H), 1.24 (s, 3H), 1.13 (s, 3H), 1.10 (s, 3H), 0.60 (s, 3H). ^13^C NMR (150 MHz, CDCl_3_) *δ* 178.35, 177.95, 170.68, 164.94, 146.14, **141.49**, 134.37, 127.55, **127.27**, **125.55**, **124.13**, 119.64, 118.19, 117.34, 45.20, 44.45, 43.21, **41.01**, 40.50, 39.49, 38.33, 36.48, 35.05, 33.86, 33.63, 31.73, 31.17, 30.92, 30.26, **29.67**, 29.56, 28.79, 21.87, 18.53, 10.42.

##### 3-Hydroxy-9β,13α-dimethyl-2-oxo-24,25,26-trinoroleana-1(10),3,5,7-tetraen-29-oic acid 2-pyridylethylamide (4n)

Red powder; yield 71%; HR-ESI-MS *m/z*: 555.3699 [M + H]^+^ (calcd for C_36_H_47_N_2_O_3_, 555.3581); ^1^H NMR (600 MHz, CDCl_3_) δ 7.22–7.14 (m, 2H), 6.98 (dd, *J* = 7.1, 1.4 Hz, 2H), 6.46 (d, *J* = 1.4 Hz, 1H), 6.29 (d, *J* = 7.1 Hz, 1H), 3.48 (s, 2H), 2.93 (q, *J* = 5.9 Hz, 2H), 2.64 (d, *J* = 9.3 Hz, 2H), 2.42 (d, *J* = 15.7 Hz, 1H), 2.19 (s, 3H), 1.40 (s, 3H), 1.23 (s, 3H), 1.11 (d, *J* = 3.9 Hz, 6H), 0.52 (s, 3H); ^13^C NMR (150 MHz, CDCl_3_) *δ* 178.46, 178.04, 170.61, 164.94, **160.14**, **148.50**, 146.12, **137.51**, 134.15, 127.47, **123.98**, **121.95**, 119.65, 118.05, 117.14, 45.17, 44.55, 43.13, 40.37, 39.44, **39.04**, 38.33, 36.55, **35.92**, 34.97, 33.92, 33.59, 31.77, 31.27, 30.90, 30.14, 29.42, 28.77, 21.84, 18.33, 10.39.

### Biological experiments

#### Reagents

DMEM-high glucose, RPMI 1640 and PBS were purchased from HyClone. Penicillin-Streptomycin (100×) (P/S) solution, 0.25% trypsin and Foetal Bovine Serum (FBS) were purchased from Gibco. DMSO was obtained from Sigma. Cell Counting Kit-8 (CCK-8), 20 × TBS and PMSF were purchased from Meilunbio^®^. SDS-PAGE Running Buffer, RIPA Lysis buffer, Western Transfer Buffer, 1M Tris-HCL, 10% SDS, 30% Acr-Bis, TEMED, BCA kit, Cell-cycle and Apoptosis Kit were purchased from Beyotime. Super ECL Detection Reagent were purchased from US Everbright^®^ Inc. Prestained Protein Ladder was purchased from Thermo Scientific. Recombinant Human IL-6 (C009) was purchased from Novoprotein. *β*-actin (C4) sc-47778 was obtained from SANTA. HRP Goat Anti-Mouse IgG was obtained from ImmunoWay. HRP Goat Anti-Rabbit IgG was obtained from LABLEAD. STAT3 (79D7) Rabbit mAb (4904 T) and p-STAT3 (Y705) Rabbit mAb (9145 T) were obtained from CST. Survivin (EP2880Y) Rabbit mAb (ab76424), MCL1 (Y37) Rabbit mAb (ab32087), JAK2 (EPR108 (2)) Rabbit mAb (ab108596) and p-JAK2 (E132) Rabbit mAb (ab32101) were obtained from Abcam. Reagents and buffer solution for SPR were obtained from GE Healthcare. Recombinant human STAT3 protein were obtained from Cusabio.

#### *Cytotoxic assay* in vitro

The cytotoxic activities of compounds were evaluated against A549, HCT-116 (cultured in RPMI 1640), HepG2 (cultured in DMEM-high glucose) by CCK-8 method. For this assay, 100 μL (5 × 10^3^/mL) cells per well were seeded in 96-well plates and allowed to incubate for 24 h. Then, the compounds with different concentrations (0.02, 0.1, 0.5, 2.5, 12.5 μM) were added. After 24 h of incubation, CCK-8 solution (10%) was added, and the plates were incubated again for another 4 h at 37 °C. The OD values at 450 nm was immediately read by a microplate reader (Tecan Infinite M200 Pro). Subsequently, the IC_50_ values were calculated by Graphpad Prism 5. Three independent experiments were performed. Data are presented as the mean ± SD (*n* = 3). By using the same method, **4m**’s toxicity to normal colon cell was detected in NCM-460 cells.

#### In silico ADMET analysis

The ADMET properties of the compounds were predicted using Qikprop module in Schrödinger 2009 platform (Schrödinger, LLC, New York, NY) with the default settings.

#### Western blot analysis

HCT-116 cells were seeded (1 × 10^7^/mL, 2 ml/orifice) in 6-well plates and incubated for 24 h. The cells were serum-starved. Then celastrol or **4m** with specific concentrations were added for 6h incubation. After 6 h, cells were stimulated by IL-6 (25 mg/mL). The cells were harvested after 30 min and then the proteins were extracted with lysis buffer and quantified by BCA method. Each sample was separated on 10% SDS-PAGE and transferred to PVDF membrane (Millipore). The membranes were blocking and sequentially incubated with primary and secondary antibodies diluted in 5% bovine serum albumin (BioSharp). Subsequently, the bands were detected in a gel imaging analysis system ((Bio-Rad. ChemiDoc XRS).

#### SPR analysis

Purified rhSTAT3 protein (optimum pH = 5.5) was immobilised on sensor chip CM5 (GE Healthcare) and carried out at 25 °C on Biacore T200 instruments (GE Healthcare). Approximately 14937 RU of STAT3 was amino coupled to a CM5 Chip (according to the manufacturer’s protocol), and another cell being left blank for reference subtraction. HBS-EP buffer was used as running buffer. The test compounds (10 mM, DMSO) were diluted to 3.91 μM, 7.81 μM, 15.63 μM, 31.25 μM, 62.5 μM, 125 μM and 250 μM using PBS buffer with 5% DMSO. The operating conditions: contact time: 80 s, flow rate: 30 μL/min, dissociation time: 180 s. The ratio of the association and dissociation rate constants was determined as theaffinity (equilibrium constants, *K_D_*). The *K_D_* (equilibrium constant) values were calculated as *k_d_* (dissociation rate constant)/*k_a_* (associated rate constant) for each interaction and determined by globally fitting with Biacore T200 evaluation software 2.0 (GE Healthcare).

#### Molecular docking

The crystal structure of the STAT3 homodimer (PDB entry: 1BG1) was downloaded from the Protein Data Bank (PDB). The molecular docking studies were performed using the flexible docking protocol in Discovery Studio 2019. Small molecules **4m** and celastrol were prepared with full minimisation. Protein STAT3 was processed with clean protein and the active site was defined based on the key residues of the pTyr705 pocket (LYS-591, ARG-609, SER-611, GLU-612, SER-613)^22^ and set the radius of the sphere as 12.0 Å . We also selected the residues LYS-591, ARG-609, SER-611, GLU-612, SER-613 for flexible processing. For other parameters, we kept them as default settings in flexible docking.

#### Molecular dynamics simulations

Molecular dynamics (MD) simulations were performed to study the stability of molecular docking between **4m** and **CEL** with STAT3 in nanosecond scales for the duration of 100 ns. NAMD 2 (NAnoscale Molecular Dynamics) software was used with default parameters and procedures. The NPT ensemble with a temperature of 300 K and a pressure of 1 bar was applied to all the runs. The simulation length was 100 ns with relaxation time one ps for the ligand with STAT3. The docked complex system was solvated by a pre-equilibrated simple point charge in an orthorhombic box with a box wall distance of 10 Å. The system was neutralised by adding a salt concentration of 0.15 M salt (NaCl). The generated trajectories were then aligned and analysed by calculating their root mean square deviations (RMSD).

#### Cell-cycle and apoptosis assay

HCT-116 cells (log phases) were seeded in 6-well plates, and preincubated at 37 °C for 24 h. Then cells were further incubated with DMSO (blank control) or **4m** at different concentrations (1, 2.5, 5 μM) for 48 h. After the respective period of incubation, cells were harvested softly and washed twice with cold PBS, and collected by centrifugation. For apoptosis analysis, after centrifugation, the cells were resuspended with 1 × binding buffer solution and partial shipments to 100 μL. Then PI and Annexin V-FITC were added and the mixture was incubated at room temperature for 15 min in the darkness. Finally, the mixture was diluted with 1 × binding buffer and analysed by flow cytometry (Agilent Technologies, NovoCyte 3000) at 488 nm. NovoExpress software was used to analyse and generate the double dispersion point diagram. For cell-cycle analysis, the cells were fixed in 70% ethanol at 4 °C overnight.

After washing with PBS, cells were suspended in PBS containing 50 mg/ml PI and 100 mg/mL RNase A, incubated at 37 °C for 30 min, and protected from the light. Then the fluorescence intensity was measured at 488 nm by flow cytometry.

### Activity verification on colorectal cancer organoids

#### Human specimens

Malignant and primary colorectal tissues were collected through Nanjing Hospital of Chinese Medicine affiliated to Nanjing University of Chinese Medicine from patients undergoing surgical procedure at Colon and Rectal Surgical department. All experiments were reviewed and approved by the Ethics Board of Nanjing Hospital of Chinese Medicine and performed under protocols. Written informed consent form for research was acquired from donors prior to sampling procedure. Samples were acquired from adult patients who were treatment-naive. Pathological analysis of all samples was performed at department of pathology, Nanjing Hospital of Chinese Medicine.

#### Colorectal cancer organoids culture (CCOs)

Human CCOs recovered from cryopreserved organoid lines. CCOs were resuspended in Matrigel, seeded in 48-well plates and cultured in advanced DMEM/F12 medium supplemented with R-spondin 1 (500 ng/mL, Sino), Noggin (100 mg/mL, Sino), EGF (50 ng/mL, Sino), HEPES (1X, GIBCO), Glutamax (1X, GIBICO), Normocin (1X, InvivoGen), Gentamicin/amphoteritin B (1X, GIBCO), N2 (1X, Invitrogen), B27 (1X, Invitrogen), n-Acetylcysteine (1 mM, Invitrogen), Nicotinmamide (10 mM, Sigma-aldrich) and A-8301 (500 nM, Tocris). SB202190 (3 μM, Sigma-aldrich) and RHOK inhibitor Y27632 (10 μM) were added to the culture medium for organoids prior to primary seeding. The culture medium was refreshed every 48 h. CCOs were passaged every 10–14 days by dissociation mechanically with pipette tip, or with 1–2 ml TrypLETM Express (GIBCO) when needed.

#### Organoids viability assay

CCOs were seeded as described in 96 well plates. When organoids reach to 50% confluency, **L-OHP** (0.64 μM, 3.2 μM, 16 μM, 80 μM, 400 μM) and **4m** (0.024 μM, 0.12 μM, 0.60 μM, 3.0 μM, 15.0 μM) were applied to each group of CCOs respectively and DMSO was utilised as control. Cell viability was determined on 6 days post drug treatment utilising Cell-Counting Kit-8 (Shanghai Life-iLab Biotech Co., Ltd) according to the manufacturer’s instruction. The organoids were then imaged on an inverted biological microscope (Motic AS2000) with a digital camera (Moticam 2506) to detect the differences on the morphological features.

## Results and discussion

### Chemistry

**Scheme 1.**
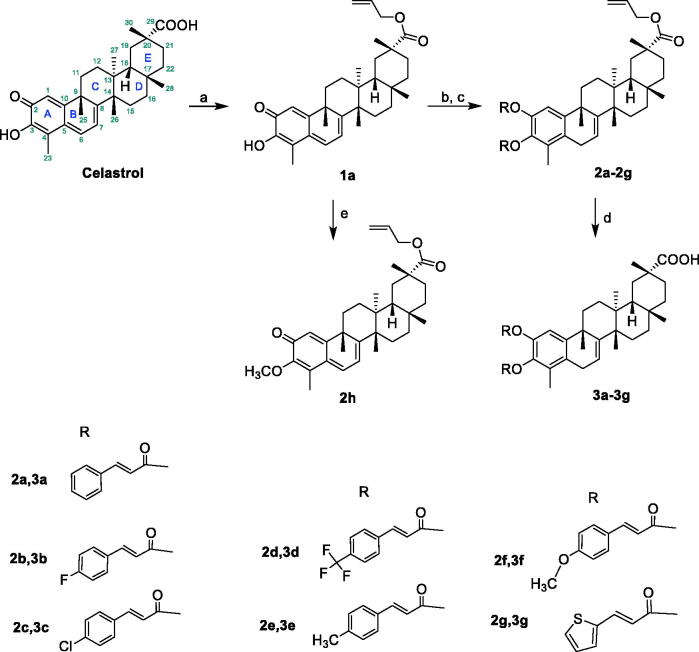
Reagents and conditions: (a) 3-allyl bromide, DMF, NaHCO_3_, rt, 12 h; (b) CH_2_Cl_2_, MeOH, NaBH_4_, rt, 0.5 h; (c) ROH, DMAP, EDCI, 70 °C, reflux, 12 h; (d) THF, Pd[P(C_6_H_5_)_3_]_4_, N-Methylaniline, rt, 1 h; (e) CH_3_I, K_2_CO_3_, DMF, rt, 8 h.

**Scheme 2.**
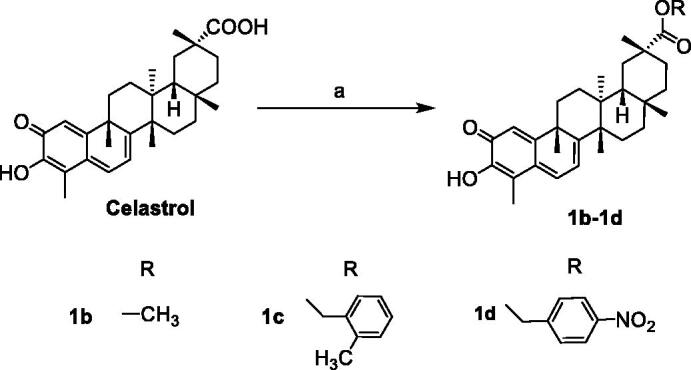
Reagents and conditions: (a) RI or RCl, Acetone or DMF, K_2_CO_3_, rt, 8 h.

**Scheme 3.**
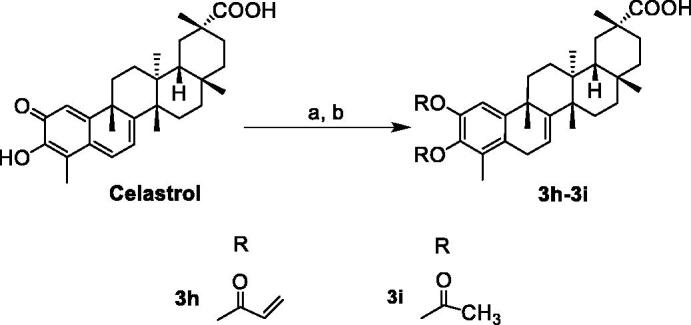
Reagents and conditions: (a) CH_2_Cl_2_, MeOH, NaBH_4_, rt, 0.5 h; (b) ROR, DMAP, TEA, 48 °C, reflux, 12 h.

**Scheme 4.**
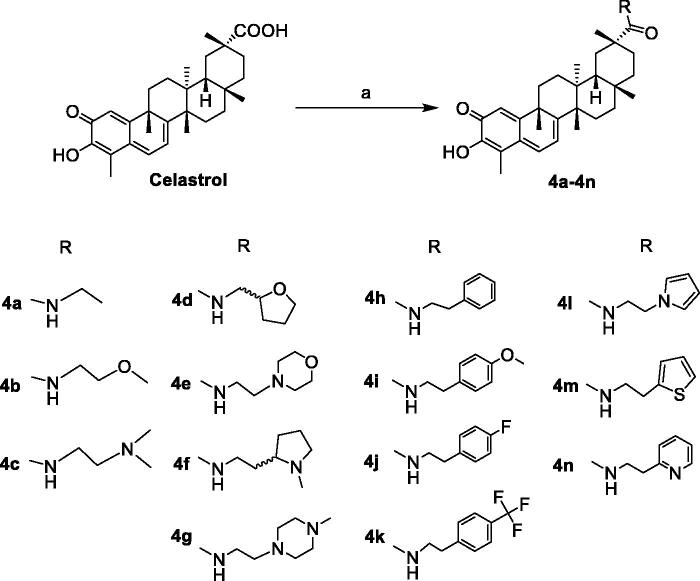
Reagents and conditions: (a) RH, CH_2_Cl_2_, BOP, DIEA, rt, 12 h.

The synthetic routes of celastrol derivatives are outlined in Schemes 1–4. The structures of the synthesised compounds were elucidated by ^1^H NMR, ^13^C NMR, and HR-MS. All spectral data were in accordance with the assumed structures.

Firstly, celastrol was esterified with the halides on the carboxyl to obtain the four C-20 ester derivatives **1a**–**1d** (Schemes 1–2). Secondly, on the basis that the carboxyl group has been protected, the C-2 carbonyl group of **1a** was reduced by NaBH_4_, and then directly esterified with cinnamic acid or other carboxy-containing compounds to obtain **2a**–**2g**. After that, their allyl group was de-protected by Pd[P(C_6_H_5_)_3_]_4_ respectively to obtain a series of novel A-ring double-substituted modified derivatives **3a**–**3g** (Schemes 1). In addition, **2h** was obtained through the methylation of **1a** with methyl iodide (Schemes 1). **3h** and **3i** were another two A-ring disubstituted derivatives produced by the reaction of celastrol with corresponding anhydride after C-2 carbonyl group reduction (Schemes 3). Finally, fourteen C-20 amide derivatives **4a**–**4n** were synthesised refer to the literature method ^23^ (Schemes 4). Among them, **2a**–**2h**, **3a**–**3h**, **4d**, **4f**, **4h**–**4n** were new synthesised compounds.

### *Cytotoxic assay* in vitro

According to the *in vitro* results (Table 1), the C-20 ester derivatives **1a**-**1d** showed more or less cytotoxicity compared with celastrol, except that **1d** have no effect on A549 under the test concentration. Almost all the C-20 amide derivatives except for **4l** showed equivalent or improved activities to at least one of the three cell lines. In particular, the antiproliferation effect of **4a**, **4d**, **4g**, **4j,** and **4m** was higher than celastrol in all the screened cell lines. We speculated that the C-29 carboxyl group contributed finitely to the celastrol’s anti-tumour activity, consistent with previous studies’ conclusions^23^^,^^24^. Thus, the structural modification of E-ring for derivatives with enhanced activity worth further investigation. In addition, the cytotoxic assay indicated that the most active compound **4m** showed lower toxicity to normal colon cells NCM-460 (IC_50_ =1.76 ± 0.42 μM) when compared with colon cancer cells HCT-116 (IC_50_ = 0.61 ± 0.07 μM).

**Table 1.** The antiproliferation effec of celastrol derivatives on three human cancer cell lines (A549, HCT-116, HepG2).

Compound		IC_50_ (μM)^a^	
	A549	HCT-116	HepG2
**Celastrol**	2.37 ± 0.02	1.40 ± 0.21	2.52 ± 0.02
**1a**	3.68 ± 0.18	0.70 ± 0.08	1.11 ± 0.04
**1b**	2.87 ± 1.44	0.79 ± 0.16	1.47 ± 0.63
**1c**	3.45 ± 0.60	2.46 ± 0.53	3.24 ± 0.05
**1d**	>12.50	1.41 ± 0.11	2.78 ± 0.17
**2a**	>12.50	>12.50	>12.50
**2b**	>12.50	>12.50	>12.50
**2c**	>12.50	>12.50	>12.50
**2d**	>12.50	>12.50	>12.50
**2e**	>12.50	>12.50	>12.50
**2f**	>12.50	>12.50	>12.50
**2g**	>12.50	>12.50	>12.50
**2h**	>12.50	>12.50	>12.50
**3a**	>12.50	>12.50	>12.50
**3b**	>12.50	>12.50	>12.50
**3c**	>12.50	>12.50	>12.50
**3d**	>12.50	>12.50	>12.50
**3e**	>12.50	>12.50	>12.50
**3f**	>12.50	>12.50	>12.50
**3g**	>12.50	>12.50	>12.0
**3h**	3.26 ± 0.66	1.50 ± 0.03	2.45 ± 0.23
**3i**	2.07 ± 0.24	1.66 ± 0.35	2.50 ± 0.02
**4a**	1.79 ± 0.01	0.75 ± 0.04	0.84 ± 0.09
**4b**	>12.50	1.38 ± 0.15	2.59 ± 0.10
**4c**	1.71 ± 0.12	1.58 ± 0.07	2.77 ± 0.14
**4d**	0.84 ± 0.04	0.80 ± 0.10	2.09 ± 0.18
**4e**	2.05 ± 0.40	1.08 ± 0.08	2.88 ± 0.04
**4f**	2.35 ± 0.17	1.95 ± 0.59	2.54 ± 0.11
**4g**	0.86 ± 0.04	0.81 ± 0.07	2.14 ± 0.03
**4h**	2.66 ± 0.52	1.15 ± 0.10	1.51 ± 0.12
**4i**	0.86 ± 0.23	0.65 ± 0.02	2.59 ± 0.24
**4j**	1.28 ± 0.10	0.70 ± 0.01	1.95 ± 0.23
**4k**	1.10 ± 0.06	0.67 ± 0.05	5.08 ± 0.01
**4l**	>12.50	>12.50	>12.50
**4m**	0.93 ± 0.03	0.61 ± 0.07	1.79 ± 0.22
**4n**	2.71 ± 0.10	1.06 ± 0.15	1.65 ± 0.07

^a^CCK8 methods, cells were incubated with corresponding compounds for 48 h. IC_50_ (μM) values (means ± SD, *n* = 3).

No matter the carboxyl group is protected or not, the A-ring disubstituted derivatives **2a–2g** and **3a–3g** had no antiproliferation effect below the maximum test concentration (12.5 μM), indicating the quinone methyl structure of celastrol is vital for activity. Significantly, celastrol was considered to affect protein function by forming covalent Michael adducts via binding to the electrophilic sites on quinone methide rings (A/B rings) of celastrol and nucleophilic thiol groups of cysteine residues^25–27^. It suggested us that preserving the unique quinone methyl structure of celastrol should be prioritised during structural modification. However, another two disubstituted compounds (**3h** and **3i**, with smaller substituent groups) retain similar activity as celastrol, and the previous study^28^ reported similar results, which contradicts our conclusion. Therefore, the structure-activity relationship of A-ring modification needs to be further analysed.

### In silico ADMET analysis of physicochemical and pharmacokinetic parameters

To evaluate active compounds’ potential for novel drug development, pharmacokinetic properties such as absorption, distribution, metabolism, excretion, and toxicity (ADMET) profiling of celastrol derivatives were determined using ADMET Predictor software. The obtained results are presented in Tables S1 in Supplementary Information. For the most active compounds **4m**, the lipophilicity was acceptable with the predicted octanol/water partition coefficient (logPo/w = 6.493). The predicted apparent Caco-2 cell permeability (PCaco) of **4m** in nm/s was 570.622, which means the ability to cross gut-blood barrier for non-active transport was great. Meanwhile, compound **4m** was expected to easily get across the blood-brain barrier, since the predicted brain/blood partition coefficient (logBB) was −0.855 and the predicted apparent MDCK cell permeability (PMDCK) in nm/sec was 628.767. Moreover, compound **4m** was predicted to be metabolised smoothly while it may undergo four kinds of metabolic reactions *in vivo*. In addition, the predicted skin permeability (logKp) of **4m** was −2.491 and the predicted human oral absorption was greater than 80%. All above indicated that **4m** has good physicochemical properties and drug development potential.

### Compound 4m inhibited the phosphorylation of STAT3 at site pTyr705

**Figure 1.**
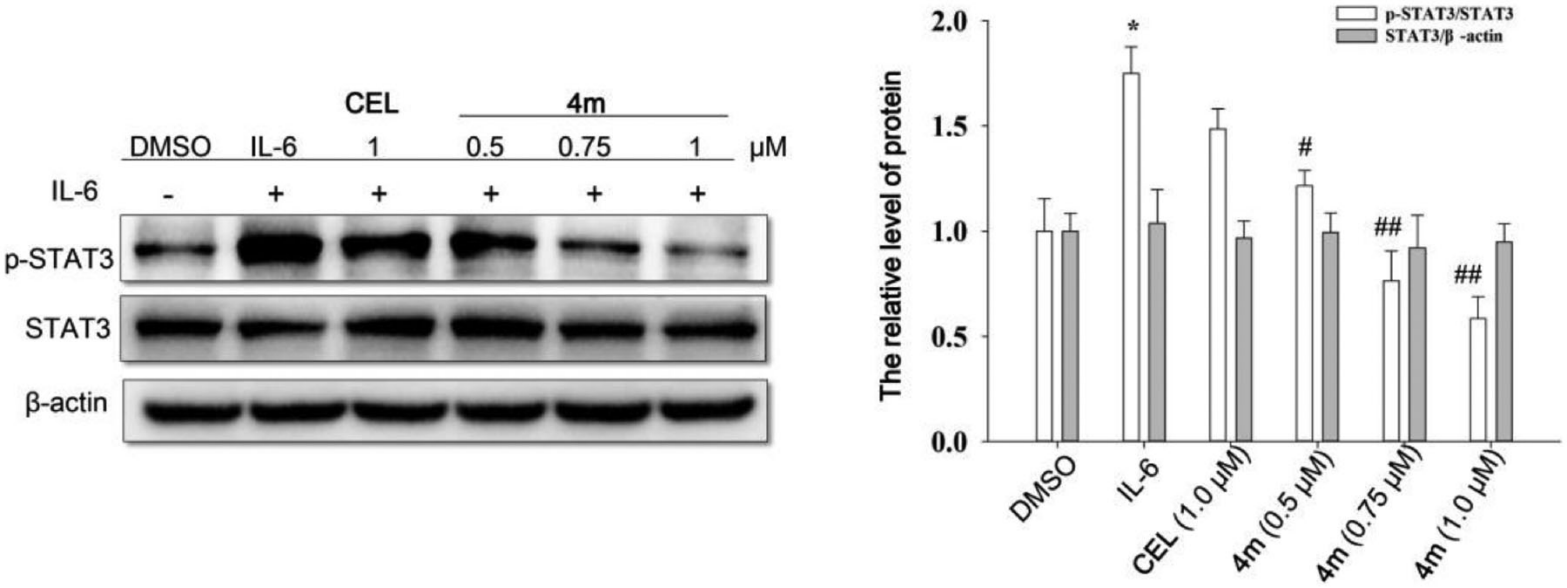
Compound **4m** inhibited STAT3 phosphorylation in HCT-116. The HCT-116 were serum-starved overnight, then left untreated or treated with **CEL** (1.0 μM) and **4m** (0.5, 0.75, 1.0 μM) for 6 h, followed by stimulation by IL-6 (25 mg/mL). The cells were harvested at 30 min and analysed by Western blot assays. Compared with DMSO (control) group, *p < 0.05; Compared with IL-6 (model) group, ^#^*p* < 0.05, ^##^*p* < 0.01.

Since celastrol had been confirmed as a STAT3 targeted inhibitor^17^, it is highly plausible that its derivatives with similar structures could function as novel STAT3 inhibitors. To further investigate whether the anti-tumour effects of **4m** were related to inhibition of STAT3 phosphorylation, we evaluated the level of p-STAT3 stimulated by IL-6 in HCT-116. As displayed in Figure 1, **4m** could effectively decrease the level of p-STAT3 at site pTyr705 in a concentration dependent manner, and the function of **4m** was stronger than **CEL** at 1.0 μM. However, the total level of STAT3 was not changed with **4m** treatment. These results indicated that **4m** could effectively inhibit the phosphorylation of STAT3 and this may be the main mechanism by which **4m** exerts its anti-tumour effect.

### Compound 4m inhibited the expression of the downstream gene of STAT3

**Figure 2.**
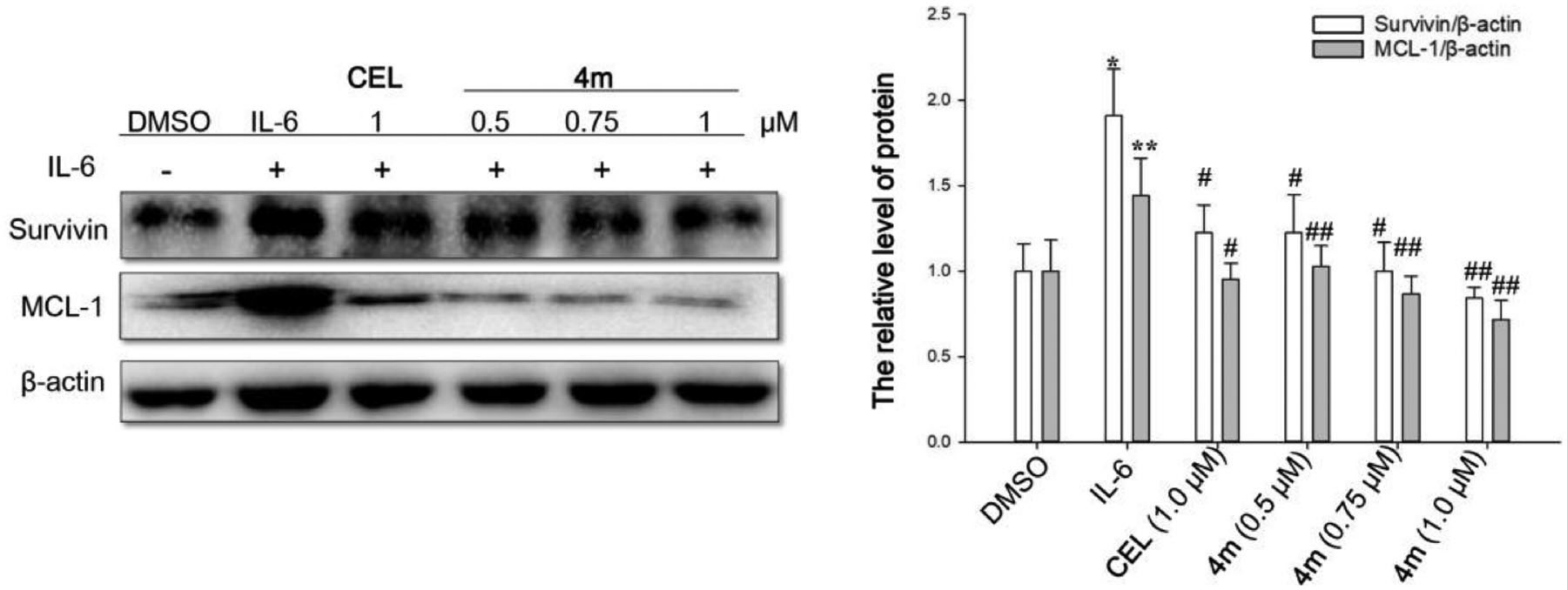
Compound **4m** inhibited STAT3 downstream genes (Survivin and Mcl-1) in HCT-116.

To further study the effects of 4**m** on the STAT3 pathway, we examined the expressions of STAT3-targeted genes (Survivin and Mcl-1). As shown in Figure 2, compound **4m** down-regulated the level of Survivin and Mcl-1. These results preliminarily indicated that **4m** could inhibit the activity of STAT3.

### Compound 4m had no effects on the typical upstream kinases JAK2

**Figure 3.**
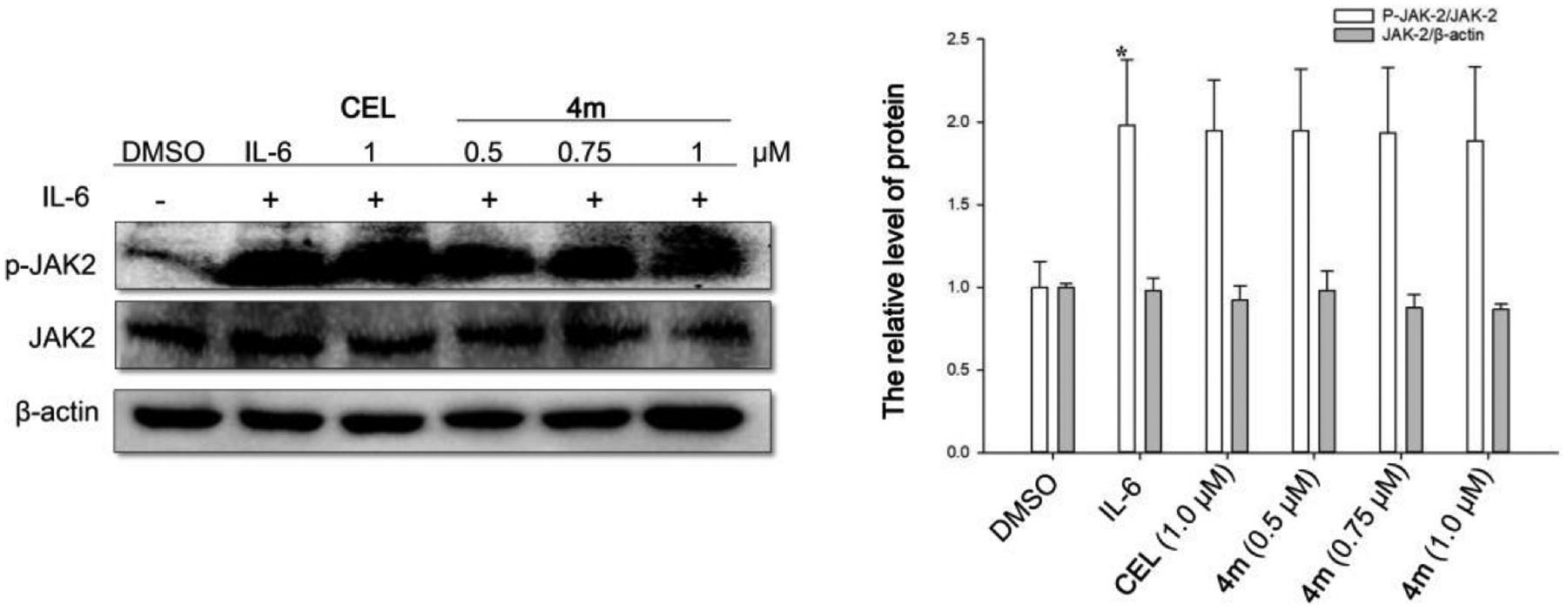
Effects of compound **4m** on STAT3 upstream tyrosine kinase (JAK2) in HCT-116.

As a signalling pathway closely related to the occurrence and development of tumours, the phosphorylation of STAT3 was usually mediated by p-JAK2, which is one of the typical STAT3 upstream tyrosine kinases^29^. To evaluate whether the inhibitory effect of **4m** on p-STAT3 is regulated by upstream JAK2, corresponding western blot was carried out. It was evident in Figure 3 that the levels of p-JAK2 and total proteins were not affected by **4m**, illustrating that **4m** might inhibit the phosphorylation of STAT3 by directly binding to STAT3 protein.

### SPR analysis of CEL and 4m to recombinant human STAT3 protein (rhSTAT3)

**Figure 4.**
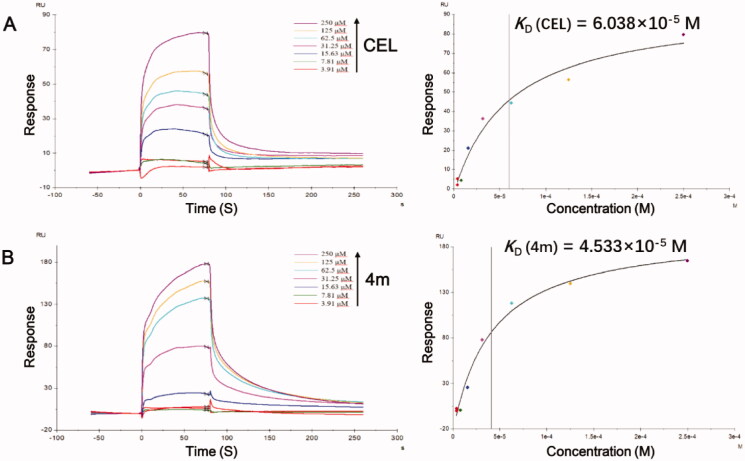
SPR analysis of **CEL** (A) and **4m** (B) with rhSTAT3 protein. Compound concentrations varied from 3.91 to 250 µM. Time measured in second (s).

Surface plasmon resonance (SPR) analysis detected the direct interaction between celastrol and STAT3 ^17^. In order to test the hypothesis that **4m** inhibits the phosphorylation of STAT3 through targeting STAT3 directly, we used SPR analysis to evaluate the interaction at the molecular level and celastrol (**CEL**) as a positive control. We observed that both **CEL** and **4m** could interact with the rhSTAT3 protein and the response unit (RU) values were proportional to compound concentrations within the selected ranges (Figure 4). According to the fitting calculation results, compound **4m** showed a slightly stronger binding affinity with a lower *K*_D_ value (the equilibrium dissociation constant) of 45.33 µM, when compared with **CEL** (*K*_D_ = 60.38 µM). In addition, the representative A-ring disubstituted derivative **3g** was also tes`ted with SPR analysis (See Figure S1 in Supplementary Information). Compound **3g** showed no creditable binding to STAT3 protein. And this might be the reason that **3g** and other A-ring disubstituted derivatives appear no effect in cell experiments. In general, the SPR experiment provides dependable evidences for the conception of screening novel STAT3 inhibitors from celastrol derivatives.

### Molecular docking between 4m, CEL, and STAT3 SH2 domain

**Figure 5.**
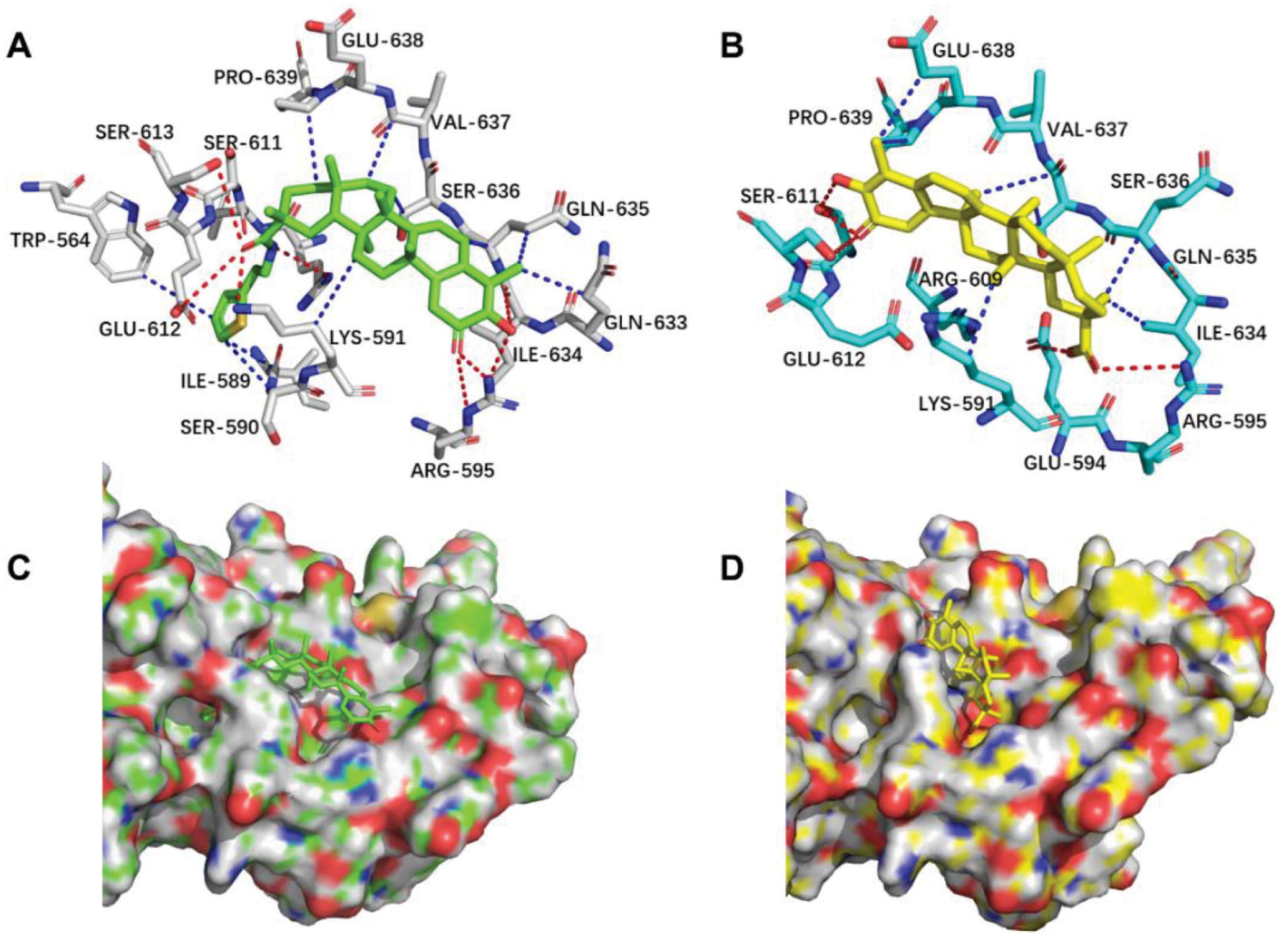
Molecular docking and binding analysis for compounds **4m** and **CEL** on STAT3 SH2 domain. (A) Binding interaction of compound **4m** (green) in key residues (gray). (B) Binding poses and interactions of **CEL** (yellow) in key residues (cyans) of STAT3 SH2 domain. (C) Binding poses of compound **4m** in surface of the binding pocket. (D) Binding poses of **CEL** in surface of the binding pocket.

As mentioned above, **4m** can bind to rhSTAT3 protein and inhibit the phosphorylation of STAT3 at site pTyr705. To further investigate the binding sites of **4m**, molecular docking was performed. As illustrated in Figure 5(A), multiple strong hydrogen bonds were formed between **4m** and the residues LYS-591, ARG-609, GLU-612, SER-613, ILE-634, ARG-595 of STAT3 protein. At the same time, **4m** had hydrophobic interactions with the residues TRP-564, ILE-589, PRO-639, VAL-637, SER-636, GLN-635, GLN-633, LYS-591. Compared with **4m**, the hydrogen bond and hydrophobic interaction between **CEL** and STAT3 protein are much less (Figure 5(B)), suggesting that **4m** binds to the SH2 domain more tightly than **CEL**, thereby inhibiting the phosphorylation of STAT3. These results were consistent with SPR analysis. Furthermore, it can be seen in Figure 5(C,D), 2-thiopheneethylamine moiety of **4m** was just inserted into the hole composed of residues SER-611, GLU-612, ILE-589, LYS-591 to enhance the affinity, indicating the size of this part just matches the size of the hole, which points out the direction for the future structural modification of **CEL**.

### Molecular dynamics simulations of the docking modes

**Figure 6.**
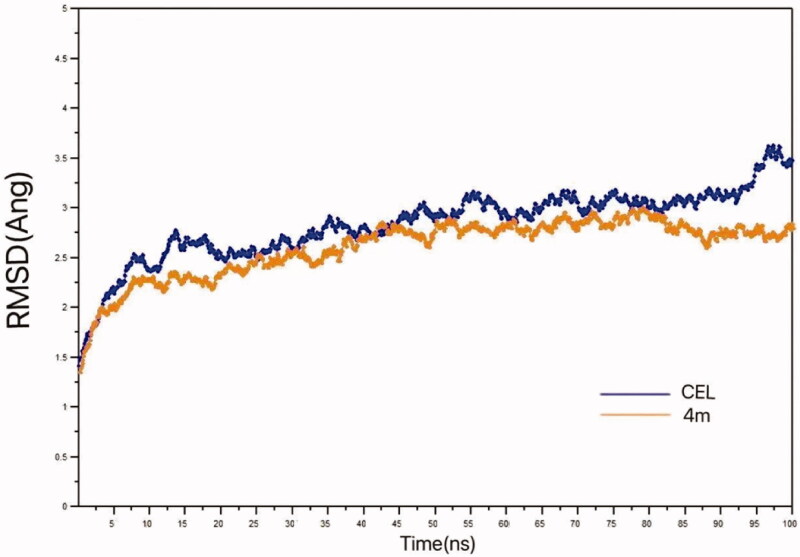
RMSD plots for the simulated STAT3-**4m** complex compared to those of the simulated STAT3-**CEL** complex.

Based on docking results, 100 ns MD simulations were carried out to estimate the dynamic stability of the docking modes of **4m** and **CEL** (Figure 6). Average RMSD of STAT3-**CEL** system increased from 1.3 to 2.8 Å around 15 ns and then increased to 3.5 Å at around 20 ns to 100 ns slowly and undulating. The average RMSD of STAT3-**4m** increased from 1.3 to 2.3 Å around 10 ns and then increased to 2.8 Å at around 10 ns to 45 ns and then reaches equilibrium from 45 ns and converges to 2.8 Å, at 100 ns. The average RMSD plot of STAT3-**4m** complex simulation has shown less deviation. It is consistently stabilised around 2.8 Å and signifies less structural deviation than the STAT3-**CEL** complex, supporting structural stability.

### Effects of 4m on cell-cycle distribution and apoptosis

**Figure 7.**
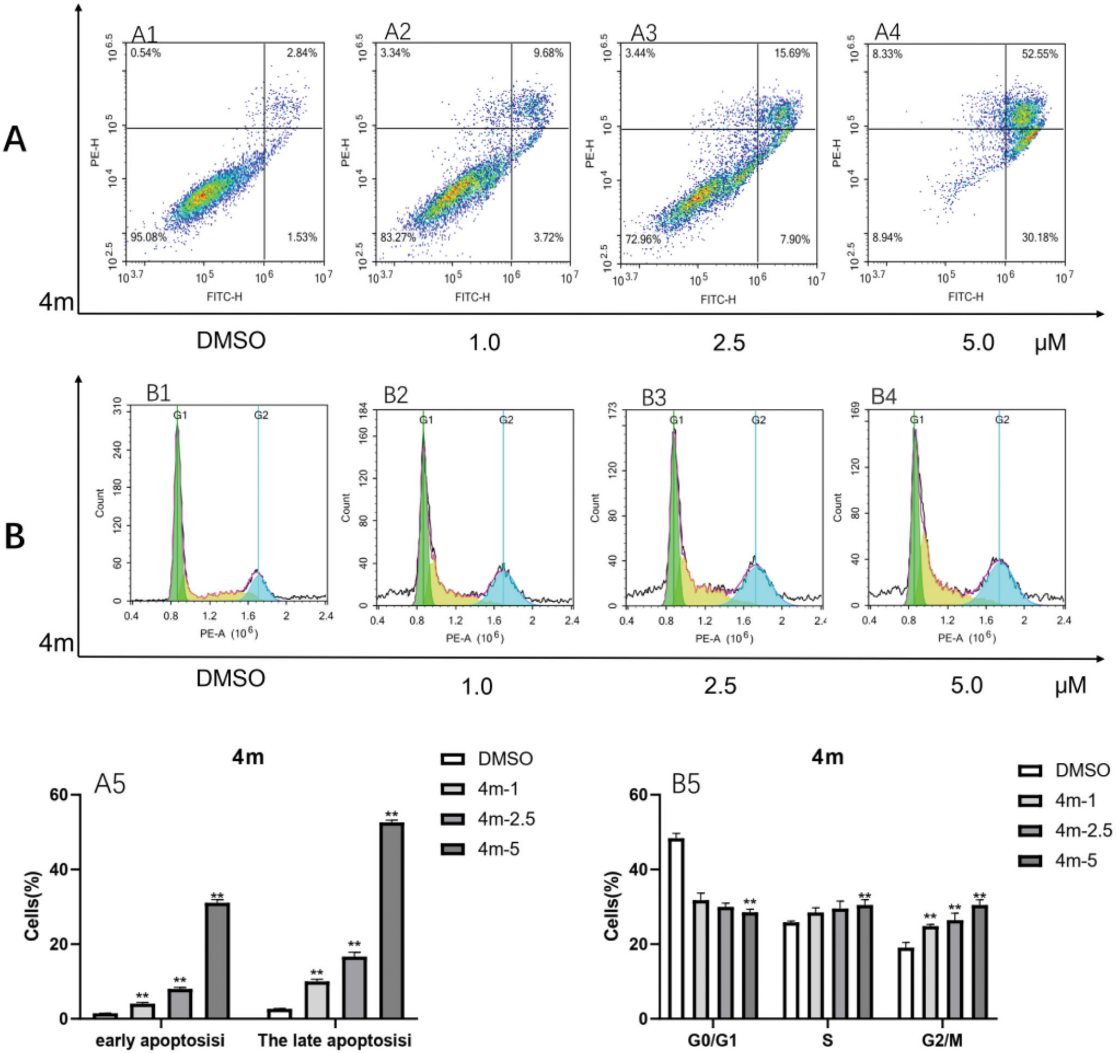
Cell-cycle analysis and apoptosis analysis of **4m** on HCT-116. Cells were treated with **4m** at distinct concentrations (1.0, 2.5, 5.0 μM) for 48 h, analysed by flow cytometry. The results were presented in the form of a column chart. The figures were representative of three independent experiments and the values were expressed as means ± SD. Compared with DMSO (control) group, **p < 0.01.

It has been reported that celastrol induces cell-cycle arrest and apoptosis in different cancer cell lines^30^^,^^31^. And there is more evidence showing that STAT3 activity has critical role in various carcinogenic processes such as preventing apoptosis and regulating cell-cycle^32^. Therefore, compound **4m** was selected for the characterisation of its effect on cell-cycle distribution and apoptosis on HCT-116. As observed in Figure 7(A), compound **4m** could induce apoptosis of HCT-116 cells in a concentration dependent manner compared with the control conditions (DMSO). Furthermore, compound **4m** also had a retardation effect on the cell-cycle distribution of HCT −116 cells compared with the control conditions. Moreover, low concentration of **4m** mainly arrested cell cycle at G2/M phase while **4m** arrested cell cycle in both S and G2/M phase at high concentration (Figure 7(B)). Hence, these results suggest that growth inhibition of the HCT-116 cells treated by **4m** may be related to induction of cell-cycle arrest and apoptosis.

### Effects of 4m on colorectal cancer organoids

**Figure 8.**
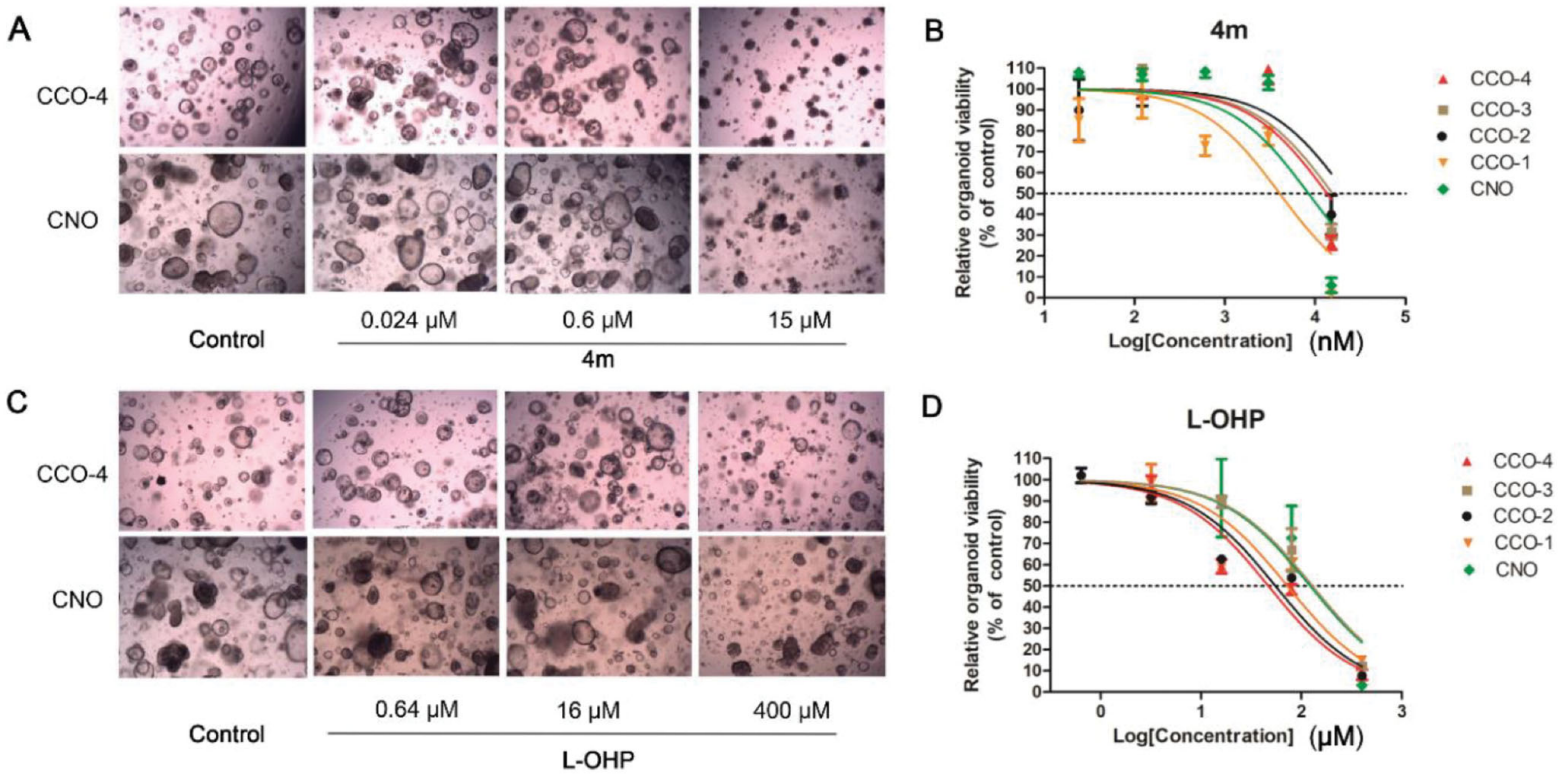
The inhibition effect of compound **4m** on human colorectal cancer organoids (CCOs) and colorectal normal organoid (CNO) was evaluated. Organoid were treated with the **4m** and oxaliplatin (**L-OHP**, positive drug) at different concentrations for 6 days and then cell viability was determined utilising CCK8. (A) Representative microscopy images of organoids incubated with **4m** at the indicated concentrations for 6 days. (B) Dose-dependent effect of **4m** on organoids viability. (C) Representative microscopy images of organoids incubated with **L-OHP** at the indicated concentrations for 6 days. (D) Dose-dependent effect of **L-OHP** on organoids viability.

Four colorectal cancer organoid cultures (CCO-1, CCO-2, CCO-3, and CCO-4) from humans were used to further investigate the activity of representative derivative **4m** and assess its potential in clinical application. The inhibitory effect of **4m** on human colorectal cancer organoids (CCOs) was evaluated. The commonly used drug oxaliplatin (**L-OHP**) in clinical was chosen as a control. Their cytotoxicity was also tested on colorectal normal organoid (CNO). As the results, **4m** showed much better activity than the positive drug **L-OHP** on all the colorectal cancer organoids (Figure 8). While **L-OHP** gradually emerges drug resistance in clinical application^33^, celastrol derivative **4m** would offer alternative as drug candidate. However, **4m** also had certain toxicity to CNO, which need to be taken into account before clinical application. As an available strategy, nano based drug delivery systems are functional to achieve optimal efficacy and reduce toxicity^34^ which can offer more opportunities for the translational development of celastrol and its derivatives.

## Conclusions

In this study, we reported the design and synthesis of a series of celastrol derivatives including 2,3-disubstitution in A-ring, esterification and amidation of C-20 carboxy group in E-ring. Some of the C-20 amides derivatives (**4a**, **4d**, **4g**, **4j**, and **4m)** showed improved anticancer activity against all the tested cancer cell lines compared with celastrol. The SAR was discussed and the preliminary results showed the possible negative effect of A-ring modifications of celastrol analogs on anticancer activity. Among all the synthesised derivatives, compound **4m** was the most potent, highly active on all tested tumour cell lines. Mechanism studies showed that **4m** exerts a significant inhibitory effect on STAT3 phosphorylation as well as the downstream genes without affecting the activated JAK2 and showed stronger binding affinity with STAT3 protein than celastrol. And the docking and molecular dynamics studies revealed that **4m** could bind to the SH2 domain of STAT3 protein. Furthermore, the flow cytometry test showed that the potent anti-tumour activity of compound **4m** on HCT-116 cells might be mediated by inducing cell-cycle arrest and apoptosis. Finally, we further verified the activity of **4m** on colorectal cancer organoids. These results indicated the potential of celastrol derivative**s** to be promising STAT3 inhibitors in the cancer treatment.

## Supplementary Material

Supplemental Material
