# Supplementary material for: Synthesis and biological evaluation of celastrol derivatives as potent antitumor agents with STAT3 inhibition
Source: J Enzyme Inhib Med Chem. 2021 Dec 11;37(1):236–51. doi: 10.1080/14756366.2021.2001805 (PMC8667935; doi:10.1080/14756366.2021.2001805)
Supplement: Supplemental Material [file IENZ_A_2001805_SM0145.pdf]

Supplementary Information

Synthesis and biological evaluation of celastrol derivatives as potent antitumor agents with STAT3 inhibition

Shaohua Xu[a,1], Ruolan Fan[a,1], Lu Wang[b,1], Weishen He[c], Haixia Ge[d], Hailan Chen[a], Wen Xu[a], Jian Zhang[e], Wei Xu[a,*], Yaqian Feng[f,*], Zhimin Fan[b,*]

[a] *College of Pharmacy, Fujian University of Traditional Chinese Medicine, Fuzhou, Fujian 350122, P.R. China.*

[b] *National Center of Colorectal Disease, Nanjing Hospital of Chinese Medicine Affiliated to Nanjing University of Chinese Medicine, Nanjing, Jiangsu 210022, P.R. China.*

[c] *Biology department, Boston college, Brighton, MA, 02135, USA.*

[d] *School of Life Sciences, Huzhou University, Huzhou, Zhejiang 313000, P.R. China.*

[e] *State Key Laboratory of Natural Medicines, China Pharmaceutical University, Nanjing, Jiangsu 210009, P.R. China.*

[f] *School of Pharmaceutical Sciences, Key Laboratory of Bioorganic Phosphorus Chemistry and Chemical Biology (Ministry of Education), Tsinghua University, Beijing 100084, P.R. China.*

[1] These authors contributed equally to this work.

*Corresponding authors: 2000017@fjtcm.edu.cn (Wei Xu); fengyq17@mails.tsinghua.edu.cn (Yaqian Feng); fanzm@njucm.edu.cn (Zhimin Fan).

Contents:

Table S1. Results of pharmacokinetic parameters of celastrol derivatives obtained with ADMET Predictor software.

Fig.S1 SPR analysis of **3g** with rhSTAT3 protein.

Fig.S2- Fig.S106 Structural characterization of the compounds (^{1}H NMR, ^{13}C NMR, ESI/HRMS spectrum of the compounds **1a-1d**, **2a-2h**, **3a-3i**, **4a-4n**).

Table S1. Results of pharmacokinetic parameters of celastrol derivatives obtained with ADMET Predictor software.

Molecule	logPo/w	logHERG	PCaco	logBB	PMDCK	logKp	#metab	logKhsa	Human Oral Absorption	Percent Human Oral Absorption
**Celastrol**	5.041	-2.069	74.313	-0.963	37.892	-4.071	2	1.054	1	76.993
**1a**	6.169	-4.874	648.628	-0.898	309.842	-3.047	3	1.686	1	100
**1b**	5.421	-4.293	595.674	-0.75	282.593	-3.473	2	1.485	1	95.396
**1c**	7.616	-5.928	832.128	-0.888	405.588	-2.485	4	2.225	1	100
**1d**	6.659	-6.056	88.244	-2.127	35.872	-4.351	4	2.055	1	74.843
**2a**	11.276	-8.741	1375.939	-1.308	698.489	-0.316	3	3.136	1	100
**2b**	11.756	-8.448	1378.162	-1.091	2280.2	-0.58	3	3.231	1	100
**2c**	12.298	-8.466	1378.484	-0.993	4252.395	-0.644	3	3.397	1	100
**2d**	13.306	-8.42	1393.405	-0.79	10000	-0.768	3	3.707	1	100
**2e**	11.958	-8.308	1175.574	-1.45	589.226	-0.911	5	3.531	1	100
**2f**	11.294	-8.171	1274.999	-1.484	643.271	-0.64	5	3.061	1	100
**2g**	10.986	-7.792	1229.208	-1.092	1970.498	-0.908	5	2.941	1	100
**2h**	6.884	-4.916	2010.674	-0.398	1052.511	-2.105	3	1.753	1	100
**3a**	10.201	-6.103	145.007	-1.57	78.046	-1.461	2	2.607	1	100
**3b**	10.815	-6.018	144.738	-1.394	253.844	-1.67	2	2.748	1	100
**3c**	11.201	-5.86	144.754	-1.276	473.301	-1.795	2	2.852	1	100
**3d**	12.209	-5.897	145.697	-1.098	1528.107	-1.897	2	3.152	1	100
**3e**	10.849	-5.845	145.76	-1.64	78.484	-1.844	4	2.943	1	100
**3f**	10.408	-5.965	156.117	-1.739	84.529	-1.555	4	2.599	1	100
**3g**	10.14	-5.547	210.135	-1.126	366.732	-1.513	4	2.431	1	100
**3h**	6.783	-3.402	145.505	-1.073	78.336	-3.034	2	1.475	1	79.458
**3i**	6.033	-2.616	118.373	-0.972	62.673	-3.708	2	1.314	1	73.46
**4a**	4.854	-3.052	565.15	-0.728	367.811	-3.172	2	1.115	1	100
**4b**	4.717	-3.291	596.888	-0.874	381.1	-2.952	3	0.937	1	91.289
**4c**	4.391	-4.26	101.359	-0.647	69.646	-5.249	3	1.049	3	75.598
**4d**	5.163	-3.497	555.974	-0.866	366.596	-3.078	3	1.17	1	80.391
**4e**	4.111	-3.976	115.362	-0.537	79.508	-5.145	4	0.889	3	74.964
**4f**	5.214	-4.529	135.046	-0.622	83.288	-5.109	3	1.373	1	69.692
**4g**	3.937	-5.072	24.492	-0.279	16.624	-7.336	4	1.042	2	61.902
**4h**	6.374	-4.141	705.36	-0.844	421.474	-2.297	3	1.579	1	89.33
**4i**	6.426	-3.856	481.271	-0.989	340.26	-2.561	4	1.601	1	86.661

**4j**	6.609	-4.018	710.956	-0.736	764.113	-2.426	3	1.621	1	90.767
**4k**	7.302	-4.03	744.276	-0.59	1755.75	-2.492	3	1.823	1	95.181
**4l**	6.082	-4.472	592.669	-0.947	374.675	-2.366	2	1.451	1	86.267
**4m**	6.493	-4.349	570.622	-0.855	628.767	-2.491	4	1.586	1	88.379
**4n**	5.982	-4.596	420.017	-1.123	263.589	-2.626	4	1.44	1	83.009

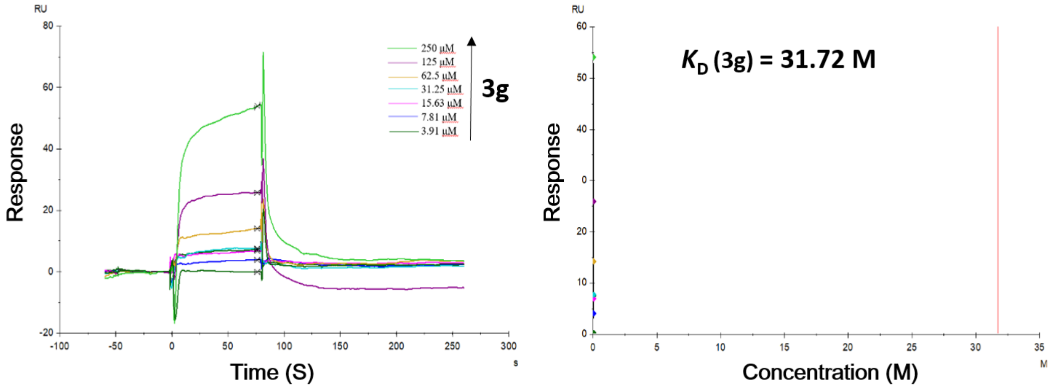

Fig. S1 SPR analysis of **3g** with rhSTAT3 protein.

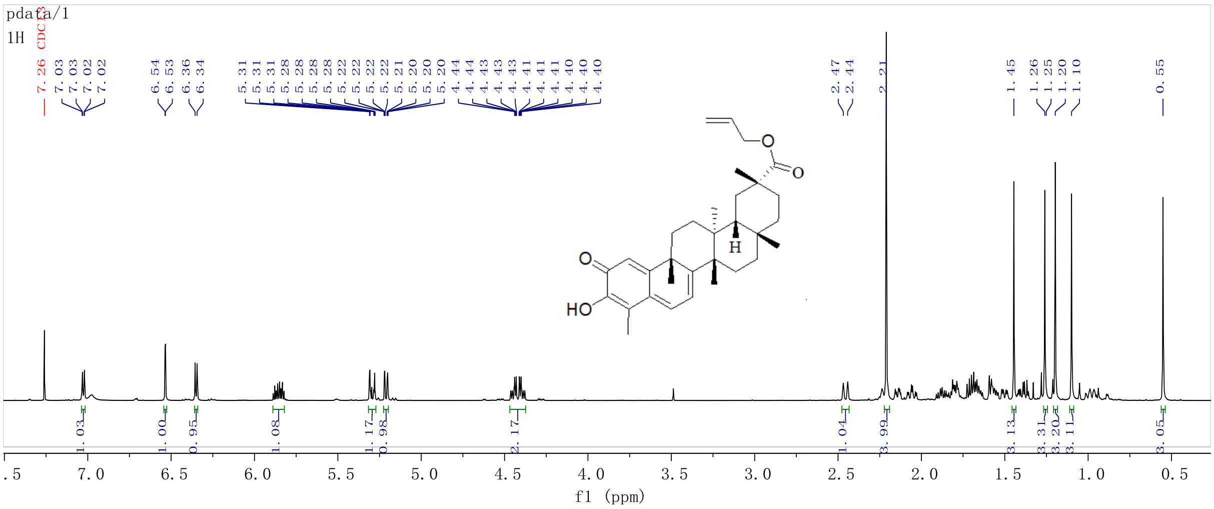

Fig. S2 ^{1}H NMR spectrum of compound **1a** ($CDCl_3$, 600 MHz)

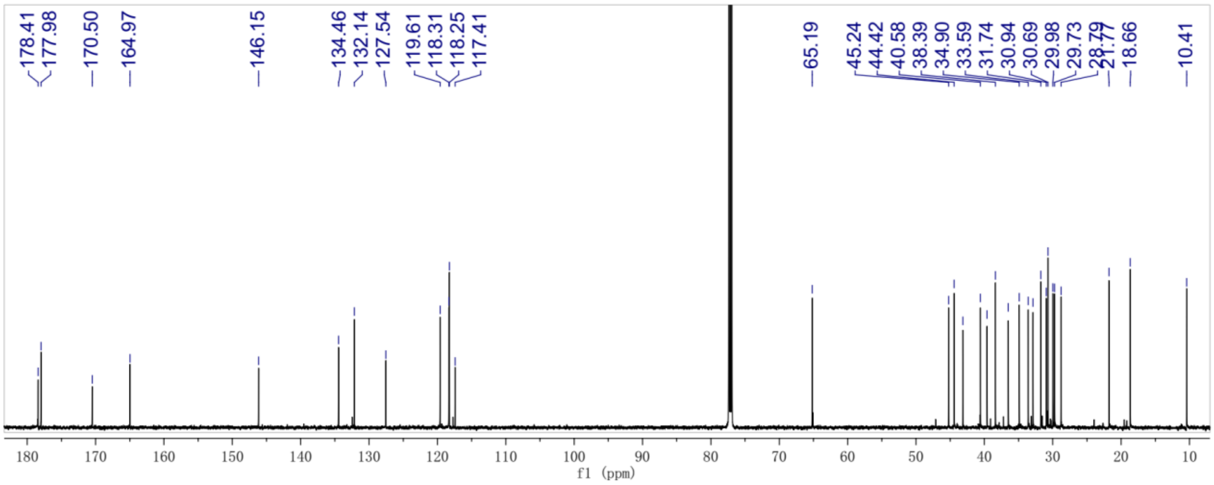

Fig. S3 ^{13}C NMR spectrum of compound **1a** ($CDCl_3$, 150 MHz)

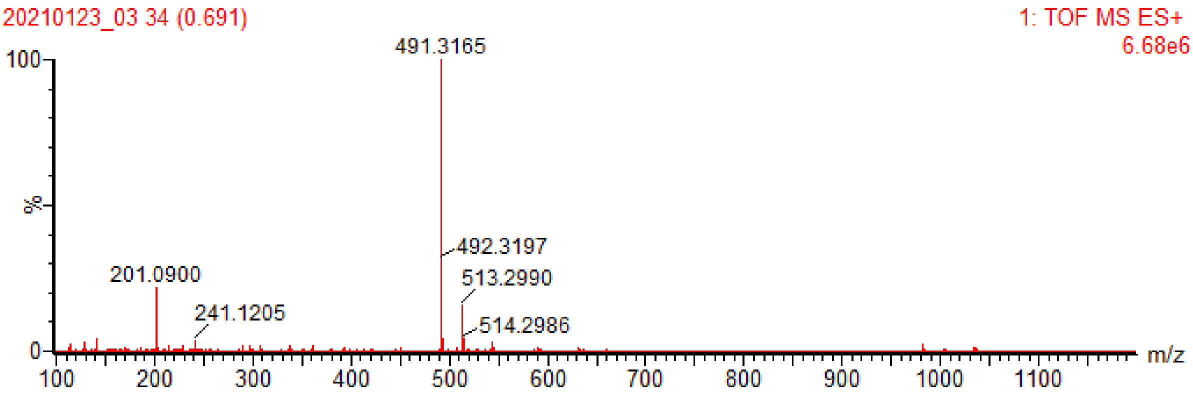

Fig. S4 HR-MS spectrum of compound **1a**

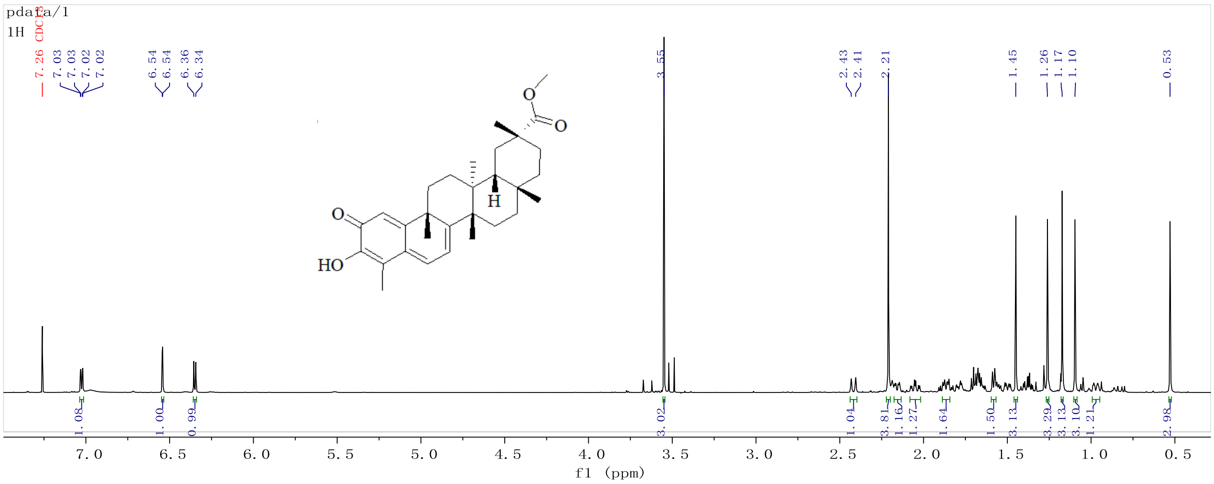

Fig. S5 ^{1}H NMR spectrum of compound **1b** ($CDCl_3$, 600 MHz)

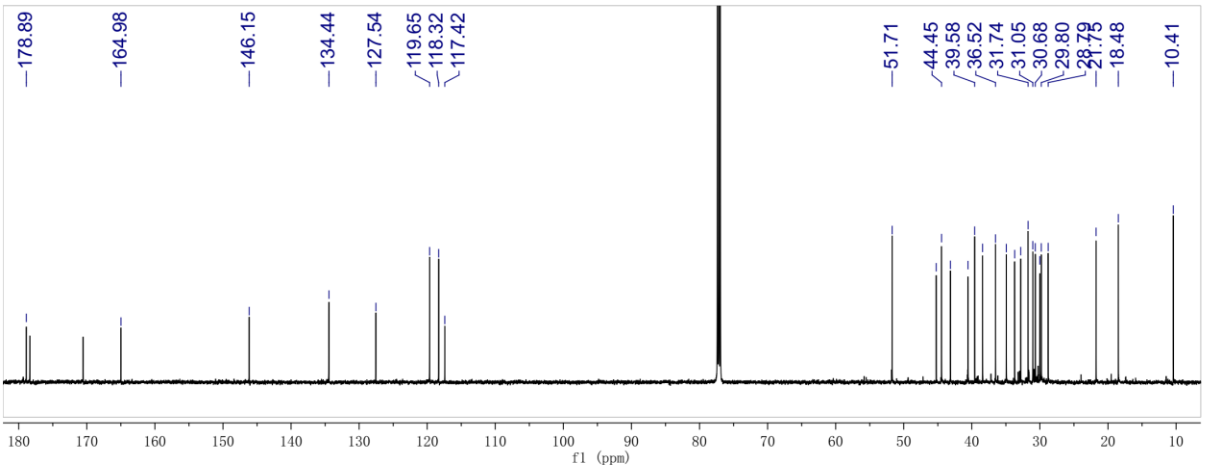

Fig. S6 ^{13}C NMR spectrum of compound **1b** ($CDCl_3$, 150 MHz)

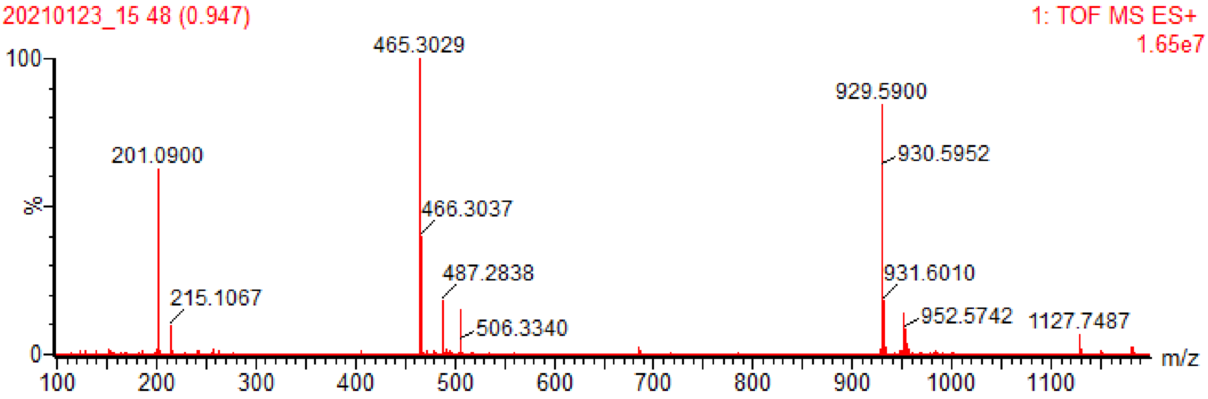

Fig. S7 HR-MS spectrum of compound **1b**

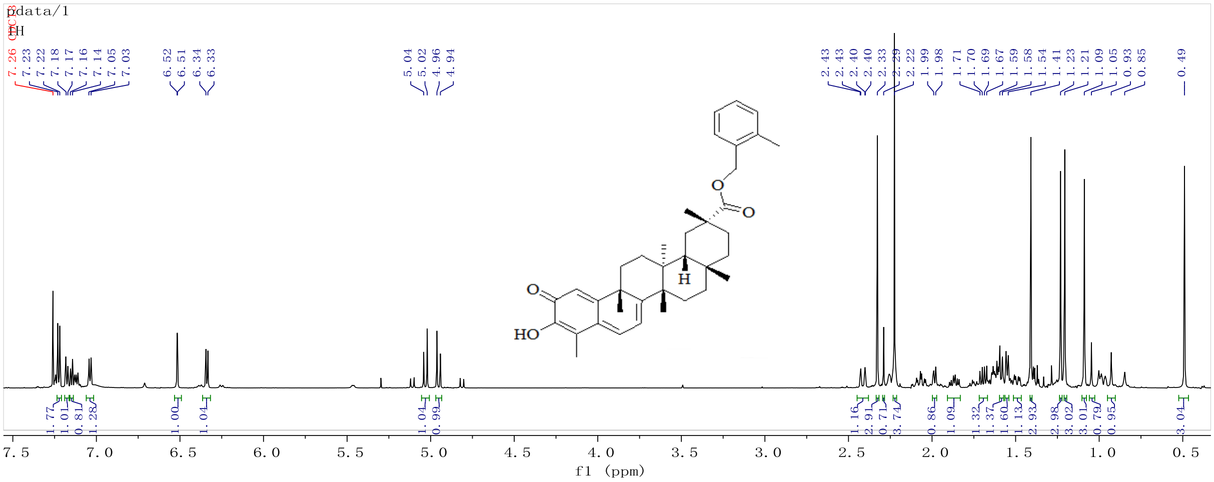

Fig. S8 ^{1}H NMR spectrum of compound **1c** ($CDCl_3$, 600 MHz)

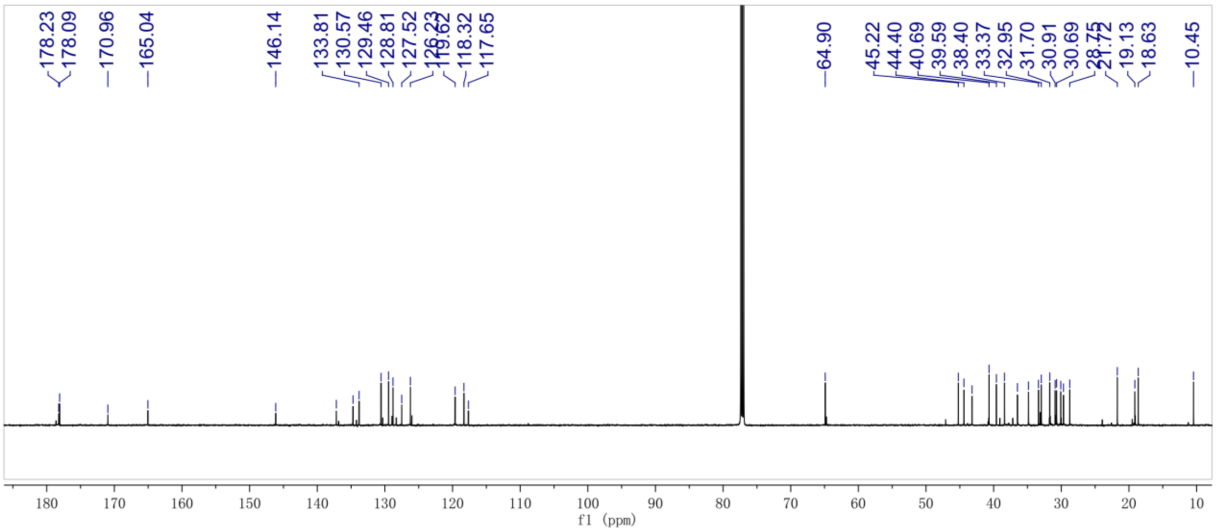

Fig. S9 ^{13}C NMR spectrum of compound **1c** ($CDCl_3$, 150 MHz)

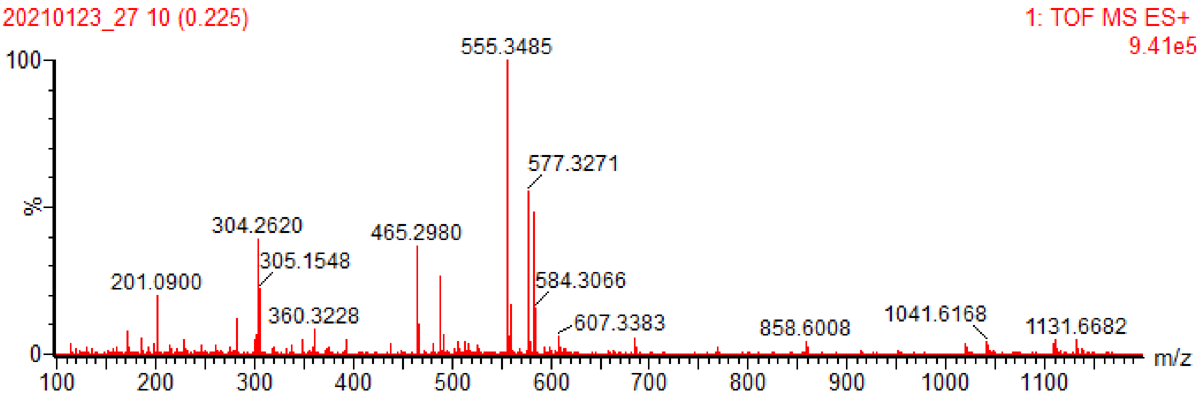

Fig. S10 HR-MS spectrum of compound **1c**

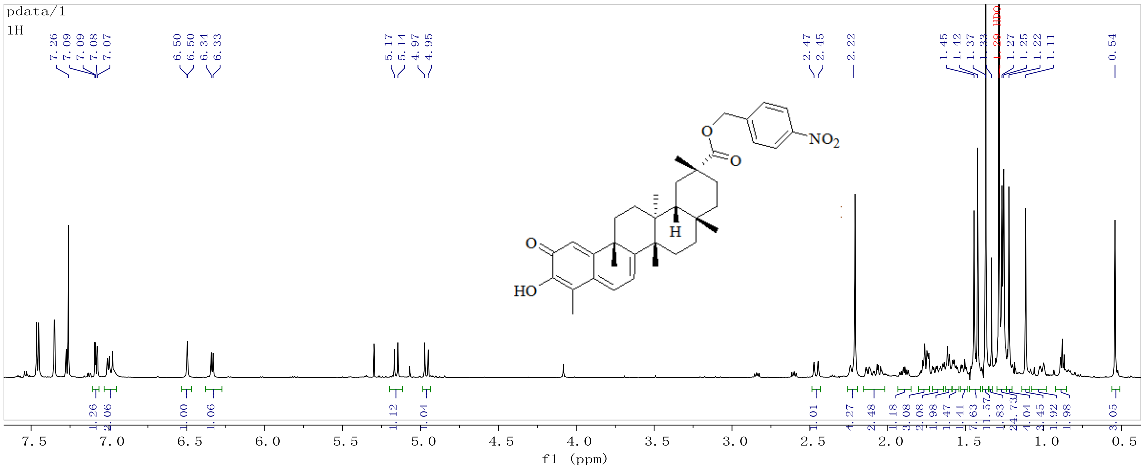

Fig. S11 ^{1}H NMR spectrum of compound **1d** ($CDCl_3$, 600 MHz)

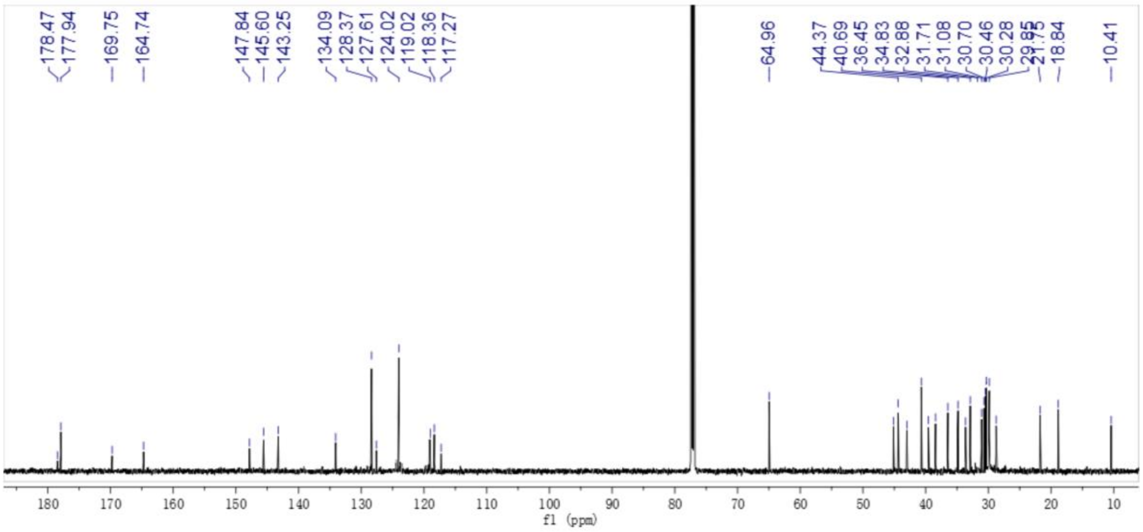

Fig. S12 ^{13}C NMR spectrum of compound **1d** ($CDCl_3$, 150 MHz)

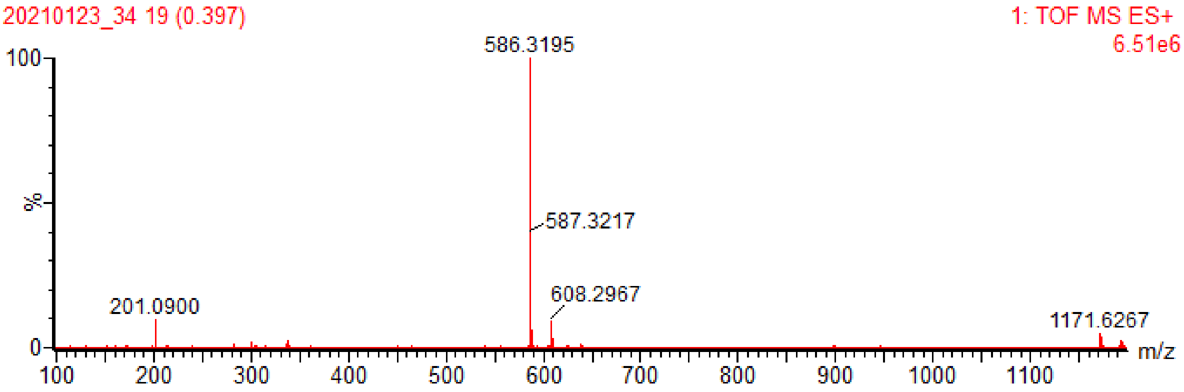

Fig. S13 HR-MS spectrum of compound **1d**

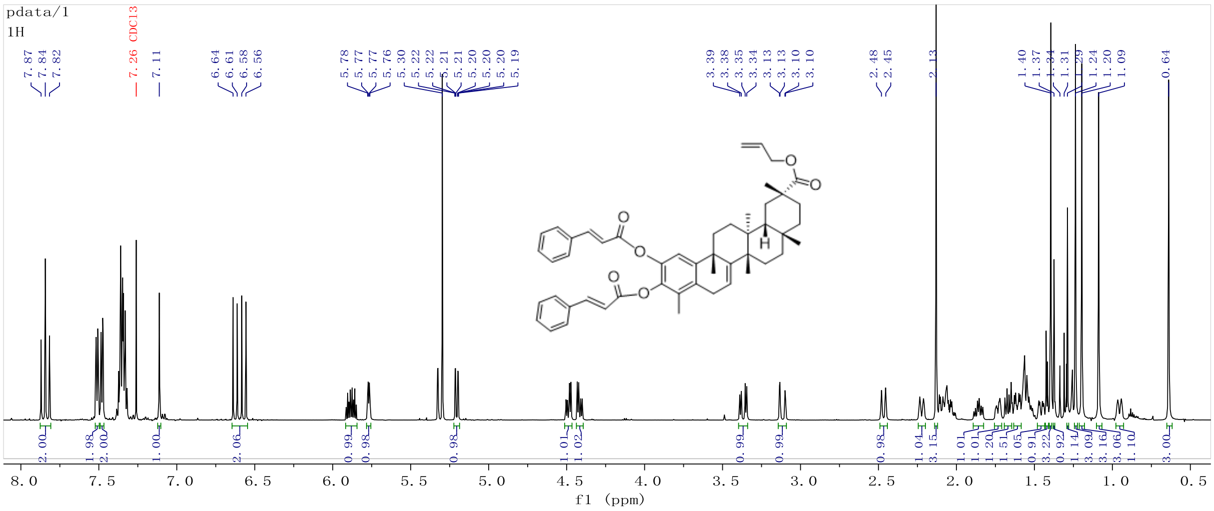

Fig. S14 ^{1}H NMR spectrum of compound **2a** ($CDCl_3$, 600 MHz)

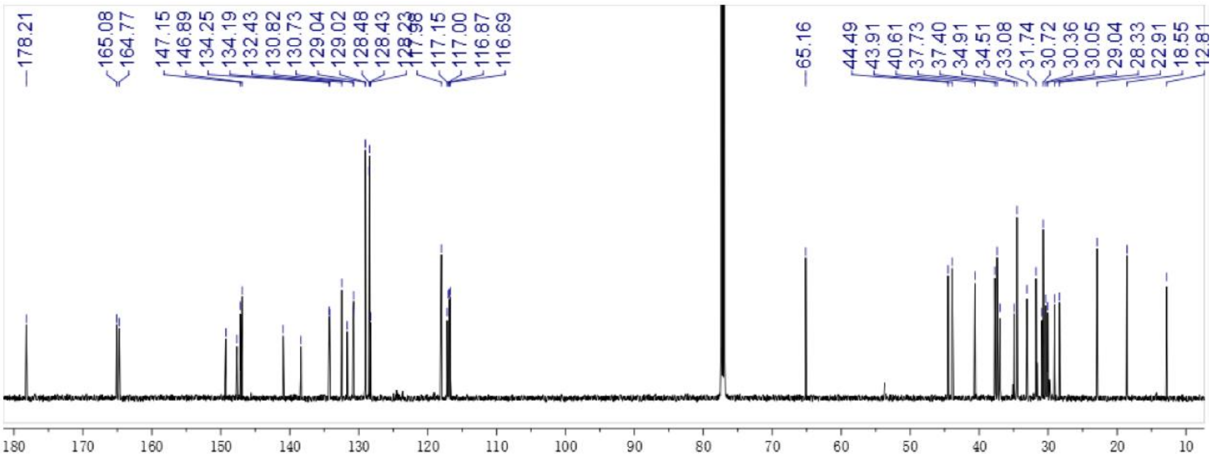

Fig. S15 ^{13}C NMR spectrum of compound **2a** ($CDCl_3$, 150 MHz)

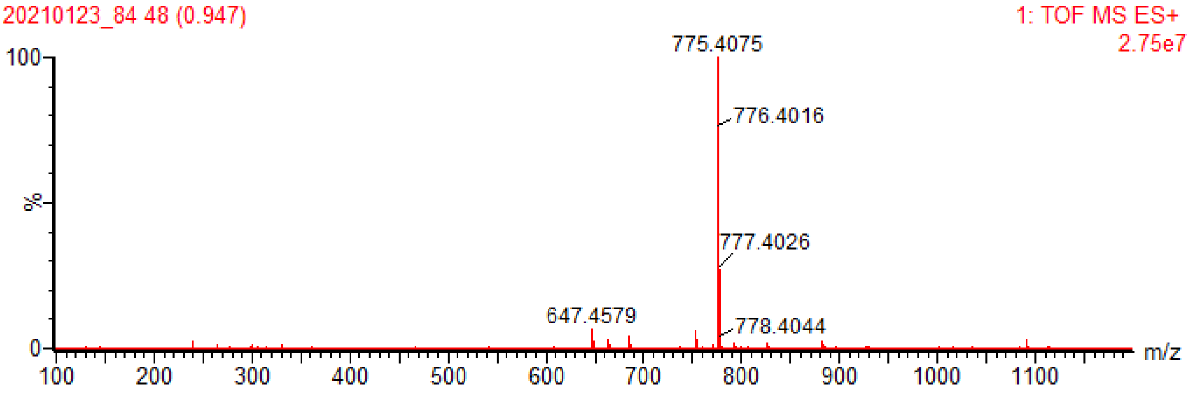

Fig. S16 HR-MS spectrum of compound **2a**

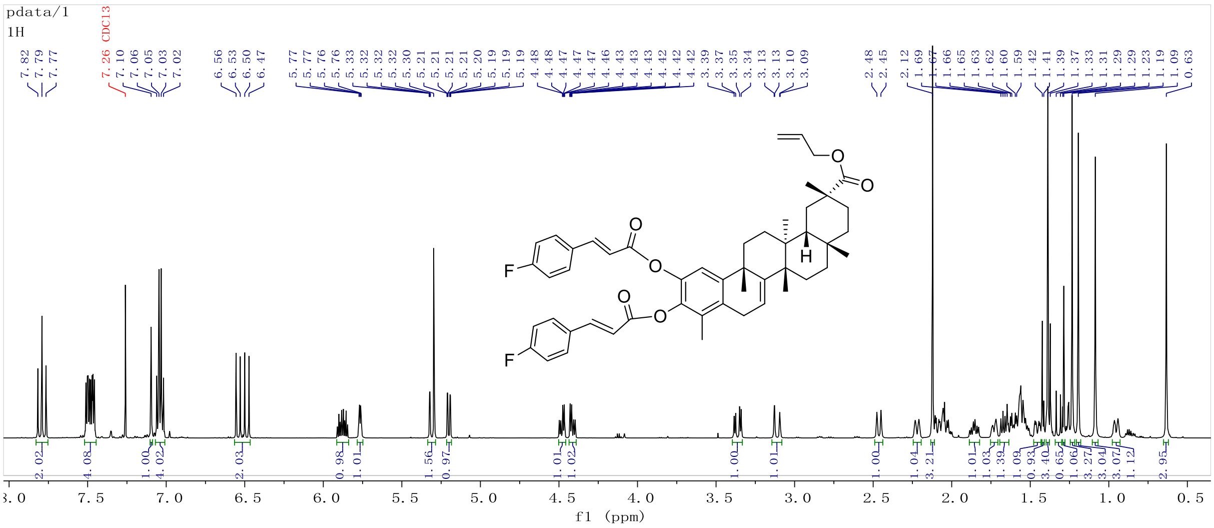

Fig. S17 ^{1}H NMR spectrum of compound **2b** ($CDCl_3$, 600 MHz)

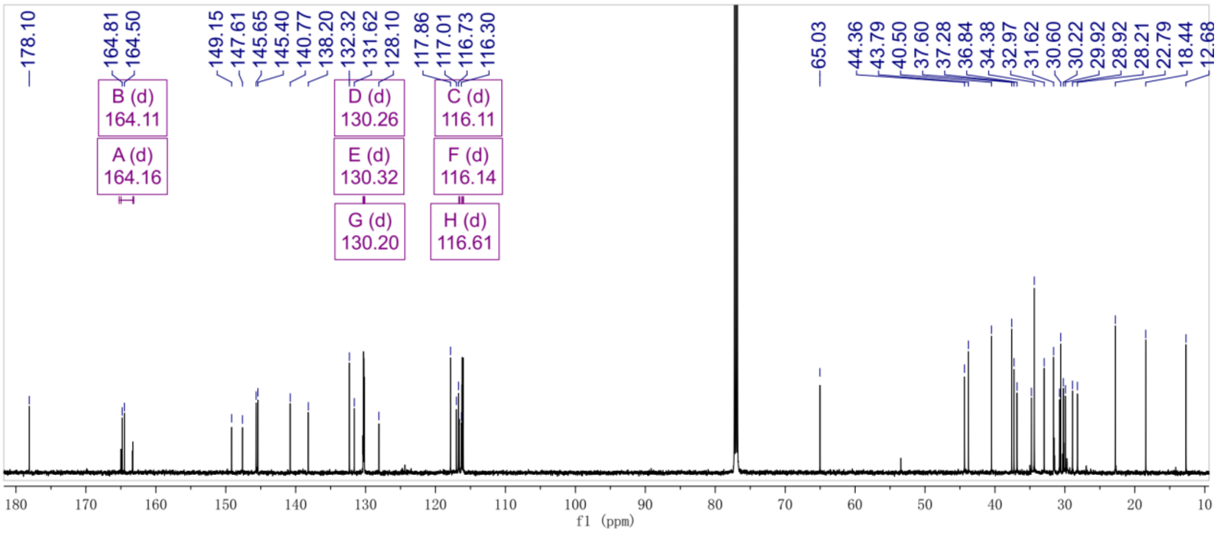

Fig. S18 ^{13}C NMR spectrum of compound **2b** ($CDCl_3$, 150 MHz)

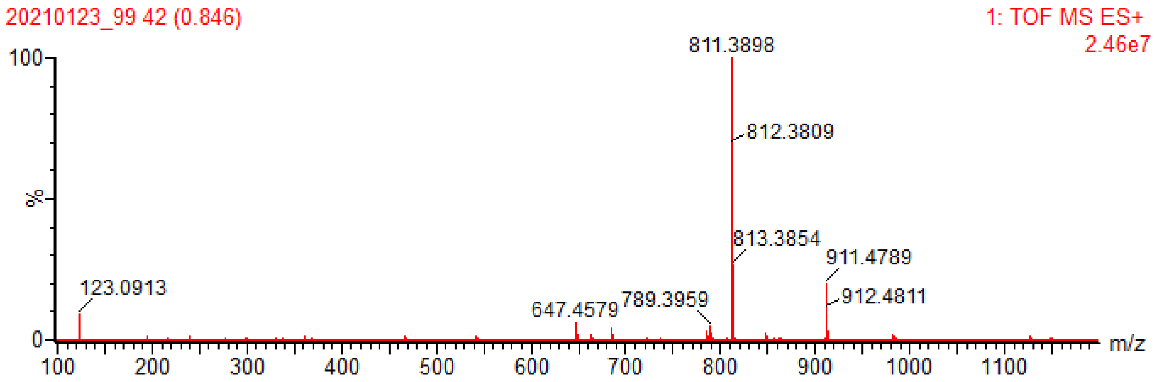

Fig. S19 HR-MS spectrum of compound **2b**

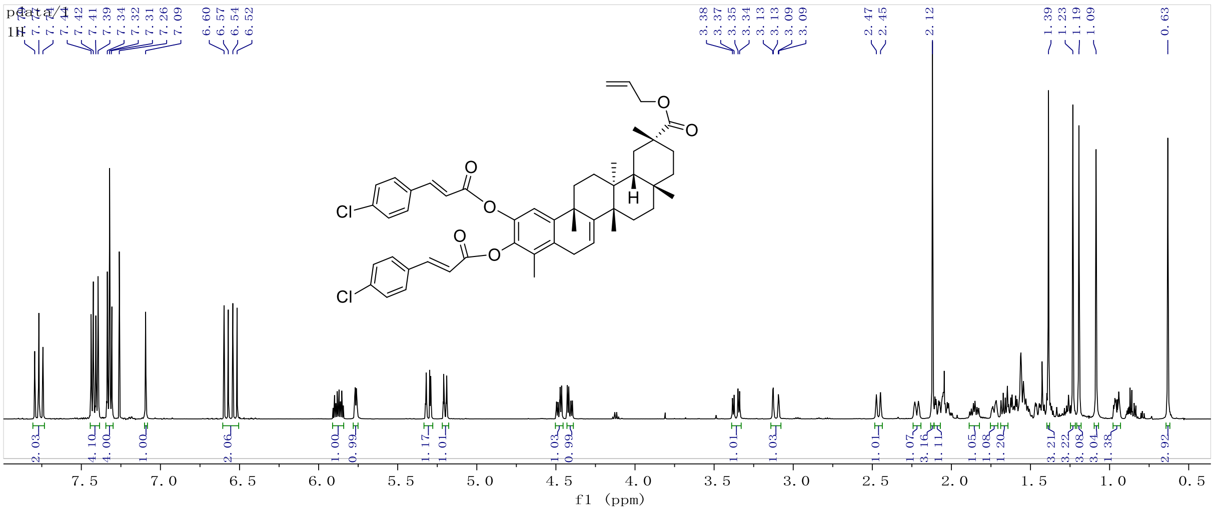

Fig. S20 ^{1}H NMR spectrum of compound **2c** ($CDCl_3$, 600 MHz)

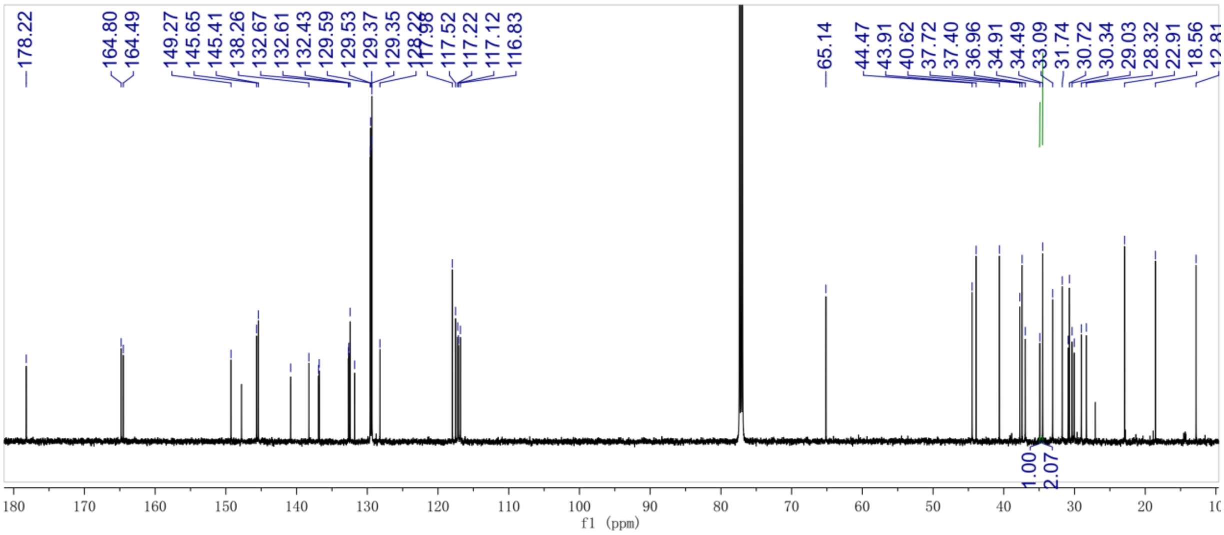

Fig. S21 ^{13}C NMR spectrum of compound **2c** ($CDCl_3$, 150 MHz)

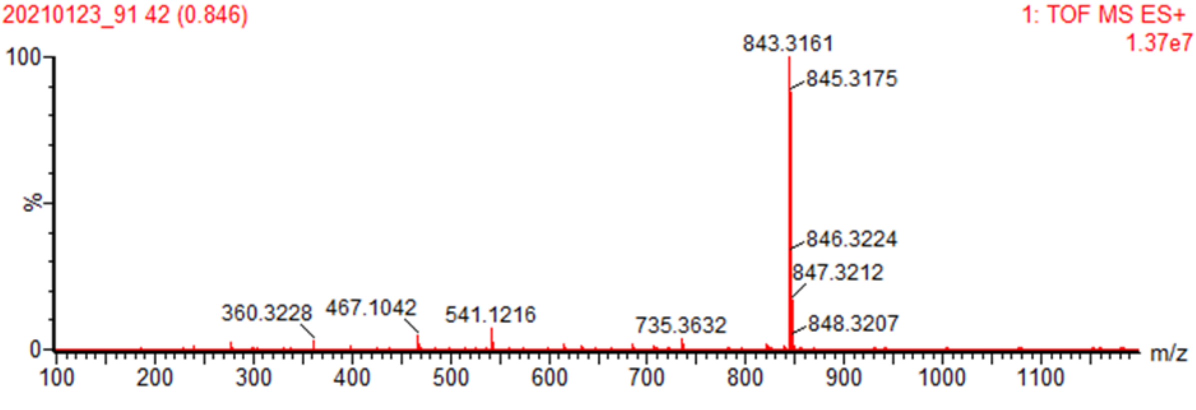

Fig. S22 HR-MS spectrum of compound **2c**

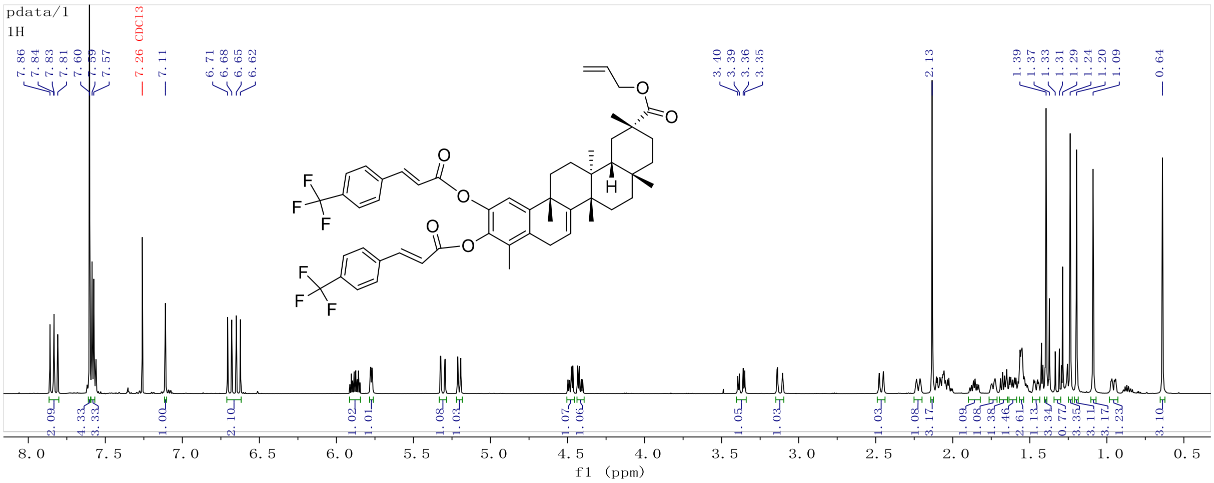

Fig. S23 ^{1}H NMR spectrum of compound **2d** ($CDCl_3$, 600 MHz)

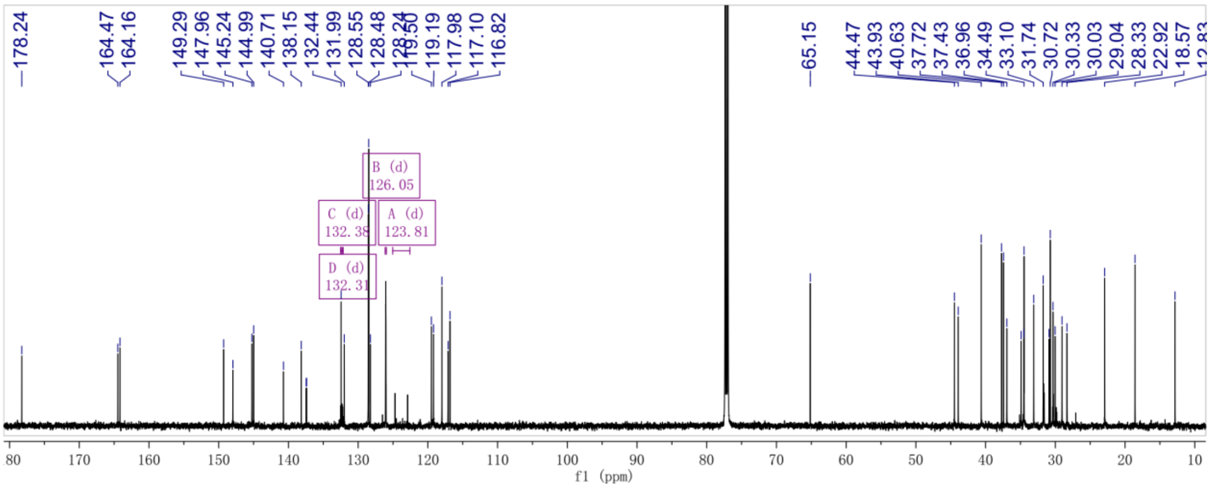

Fig. S24 ^{13}C NMR spectrum of compound **2d** ($CDCl_3$, 150 MHz)

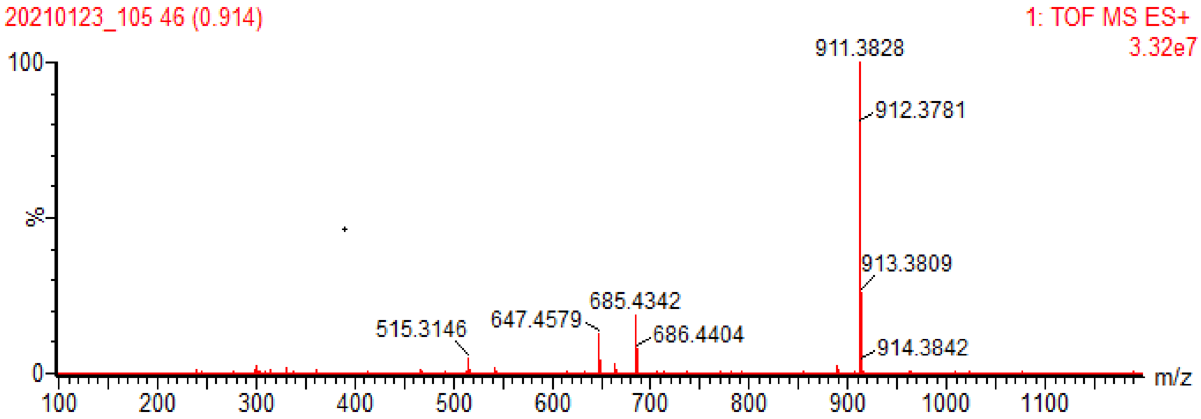

Fig. S25 HR-MS spectrum of compound **2d**

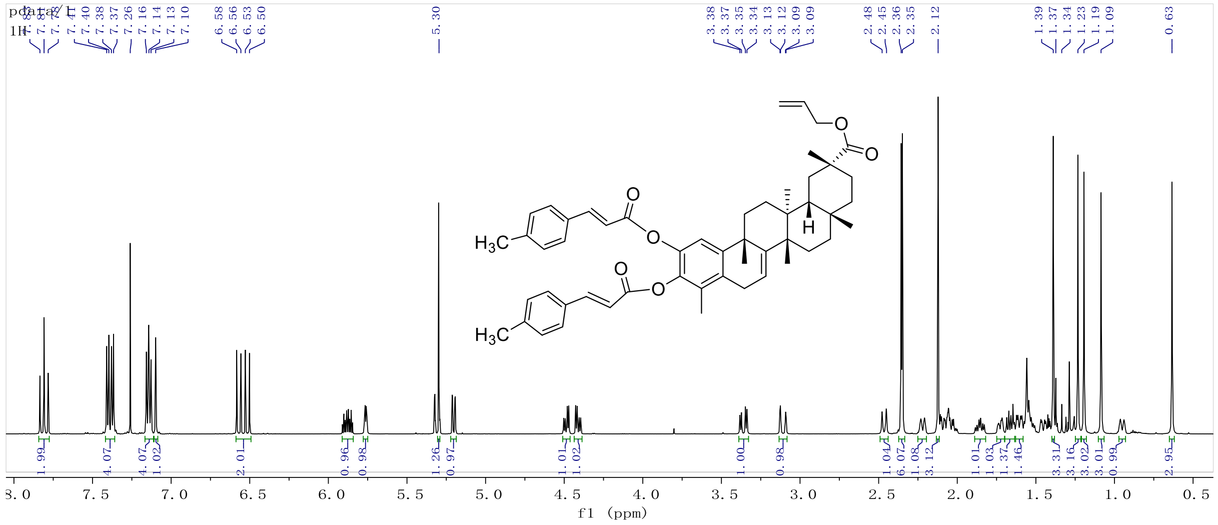

Fig. S26 ^{1}H NMR spectrum of compound **2e** ($CDCl_3$, 600 MHz)

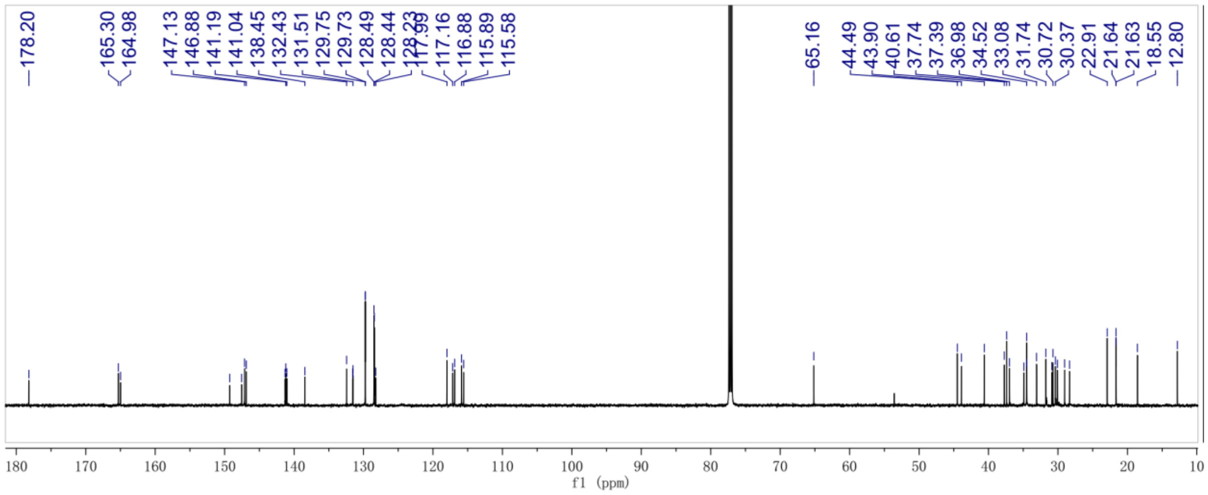

Fig. S27 ^{13}C NMR spectrum of compound **2e** ($CDCl_3$, 150 MHz)

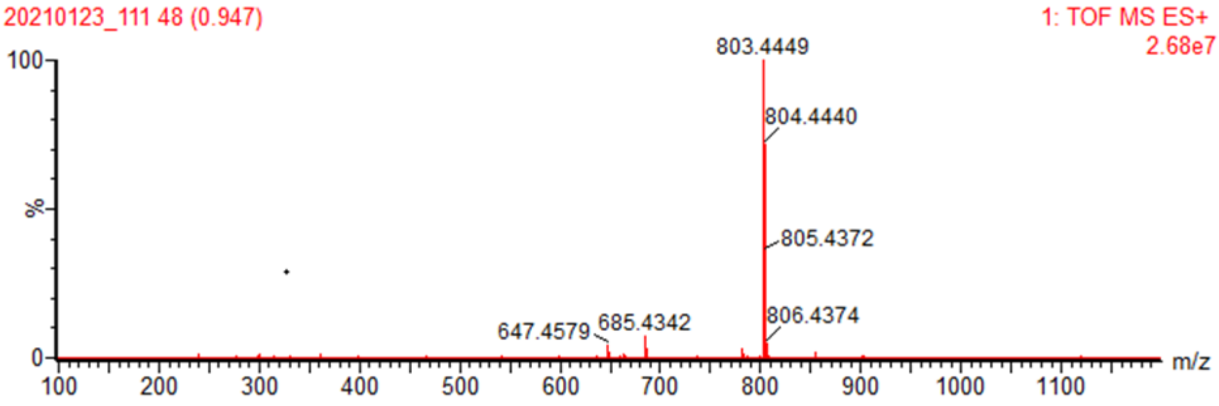

Fig. S28 HR-MS spectrum of compound **2e**

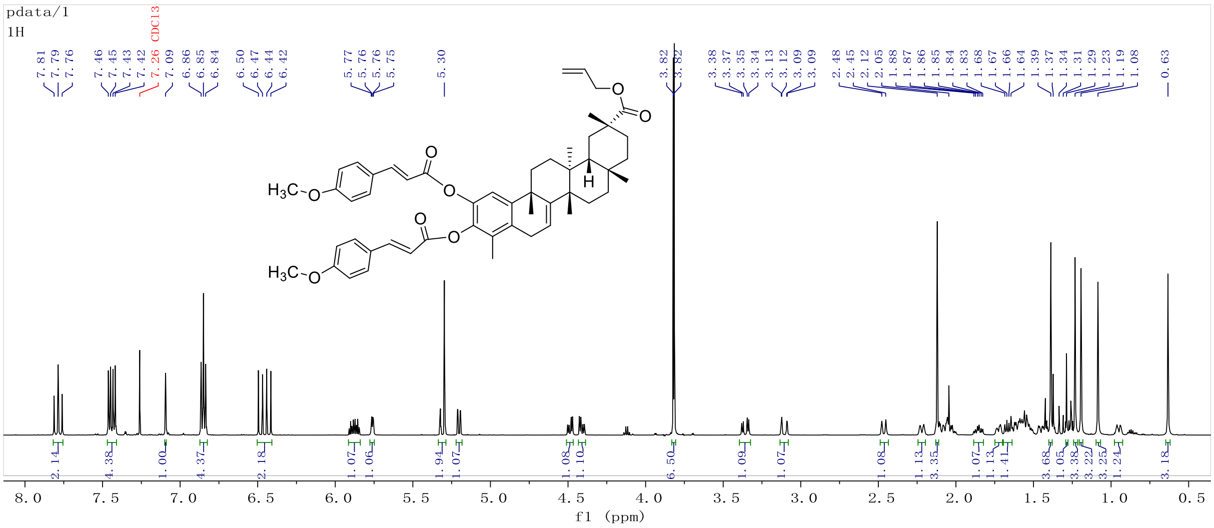

Fig. S29 ^{1}H NMR spectrum of compound **2f** ($CDCl_3$, 600 MHz)

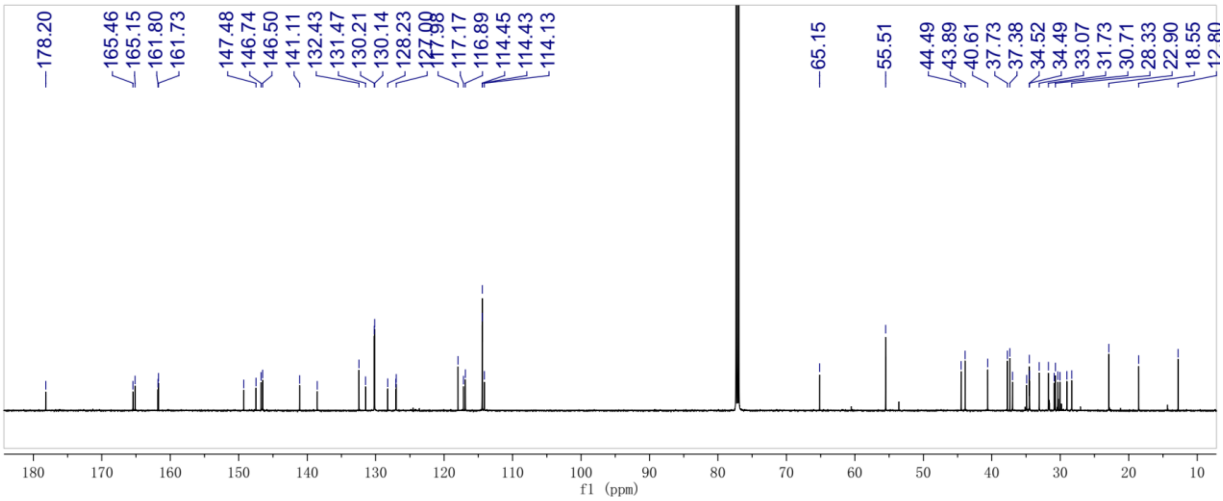

Fig. S30 ^{13}C NMR spectrum of compound **2f** ($CDCl_3$, 150 MHz)

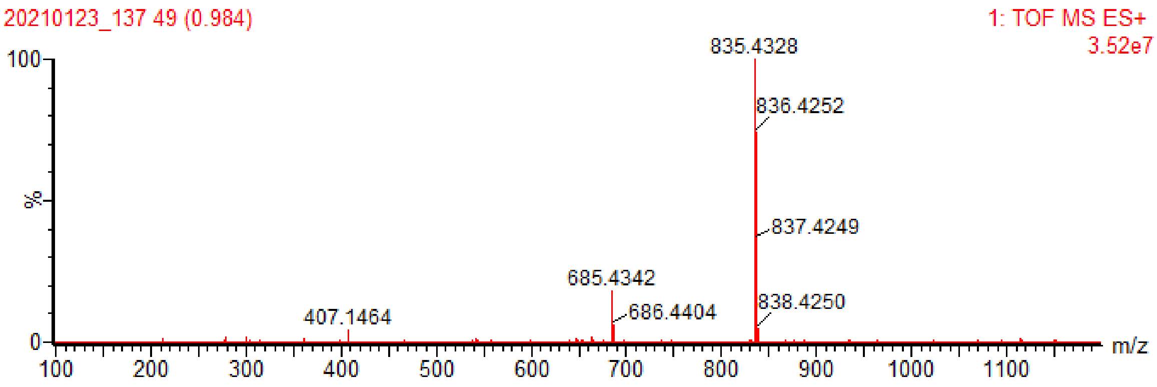

Fig. S31 HR-MS spectrum of compound **2f**

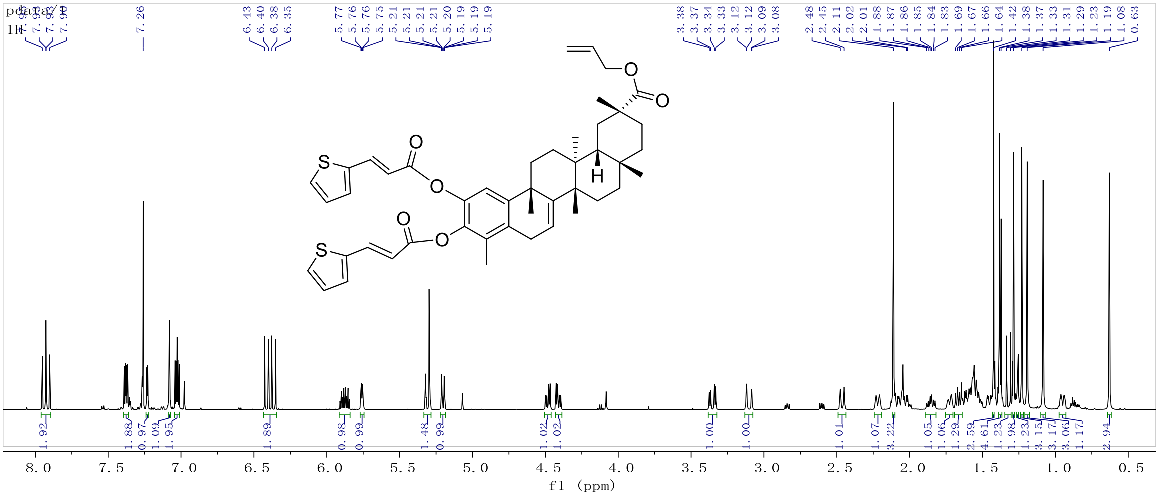

Fig. S32 ^{1}H NMR spectrum of compound **2g** ($CDCl_3$, 600 MHz)

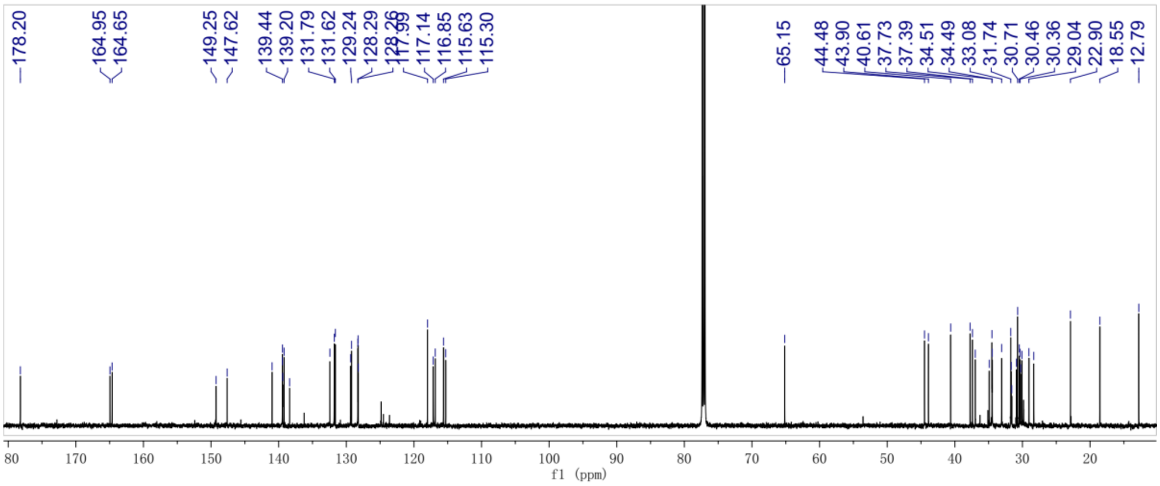

Fig. S33 ^{13}C NMR spectrum of compound **2g** ($CDCl_3$, 150 MHz)

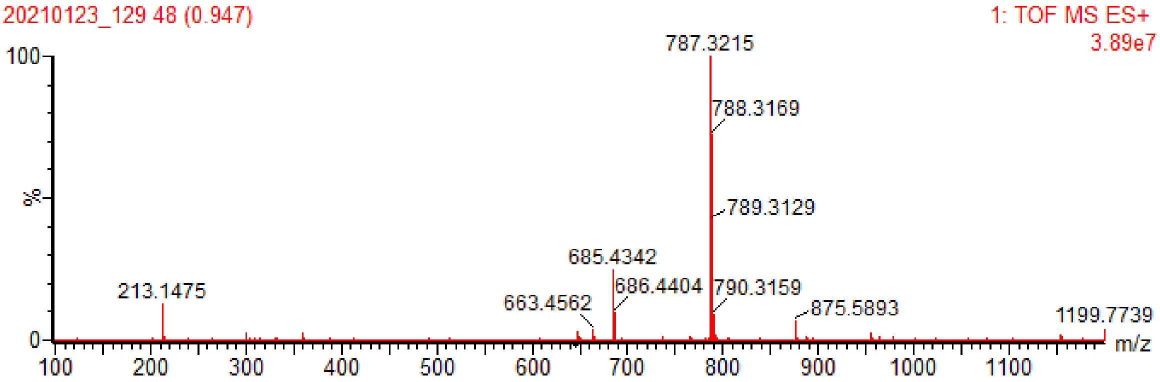

Fig. S34 HR-MS spectrum of compound **2g**

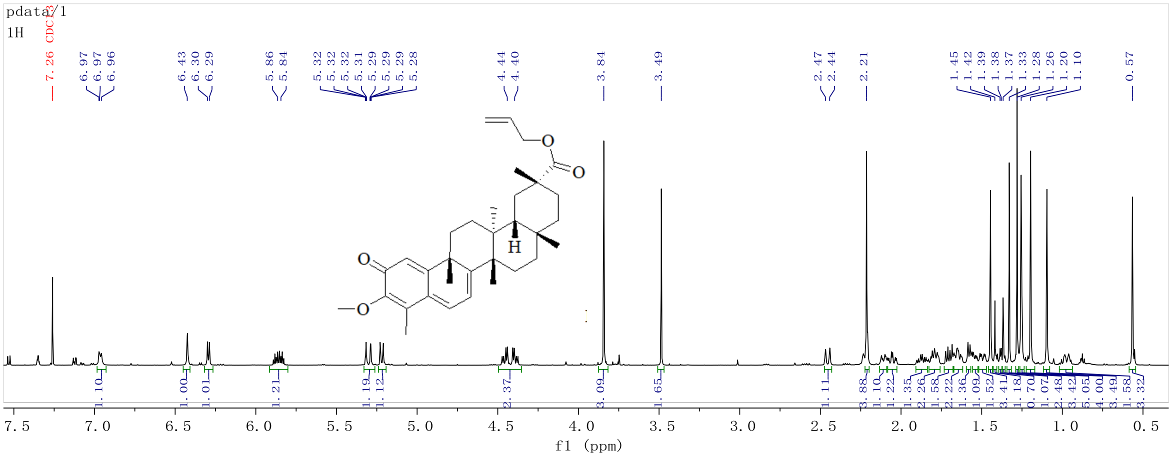

Fig. S35 ^{1}H NMR spectrum of compound **2h** ($CDCl_3$, 600 MHz)

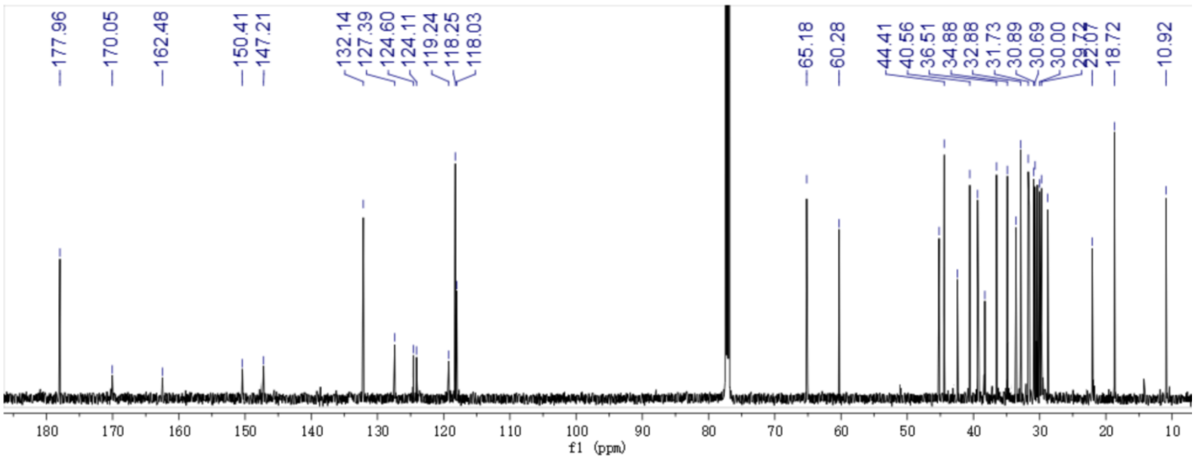

Fig. S36 ^{13}C NMR spectrum of compound **2h** ($CDCl_3$, 150 MHz)

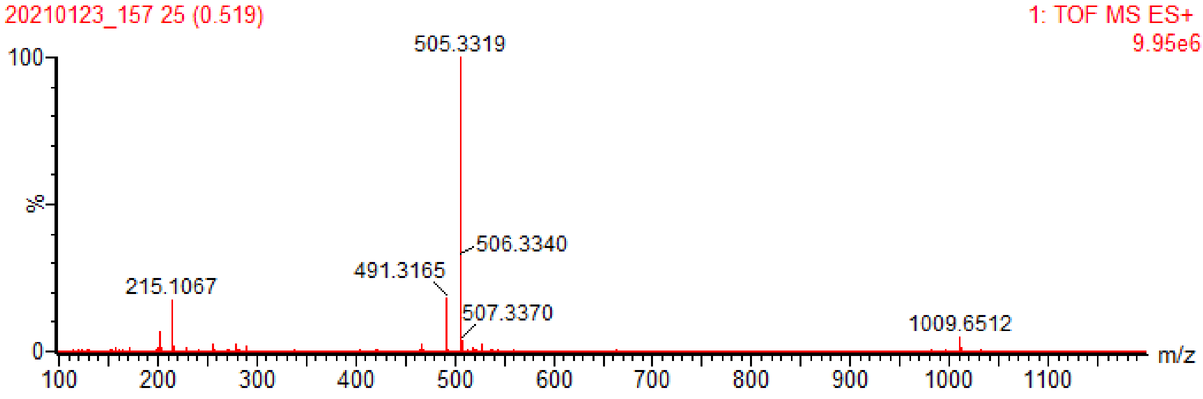

Fig. S37 HR-MS spectrum of compound **2h**

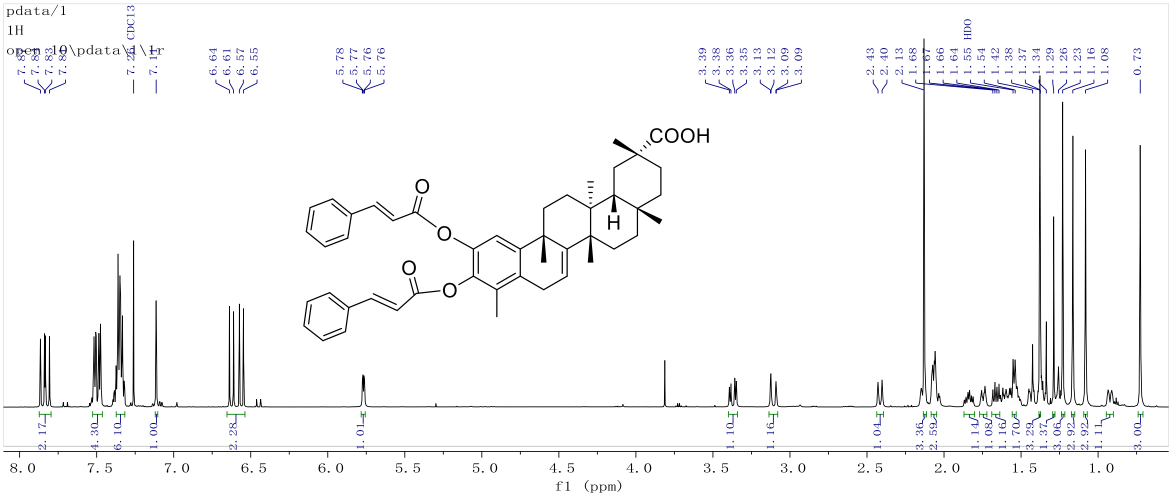

Fig. S38 ^{1}H NMR spectrum of compound **3a** ($CDCl_3$, 600 MHz)

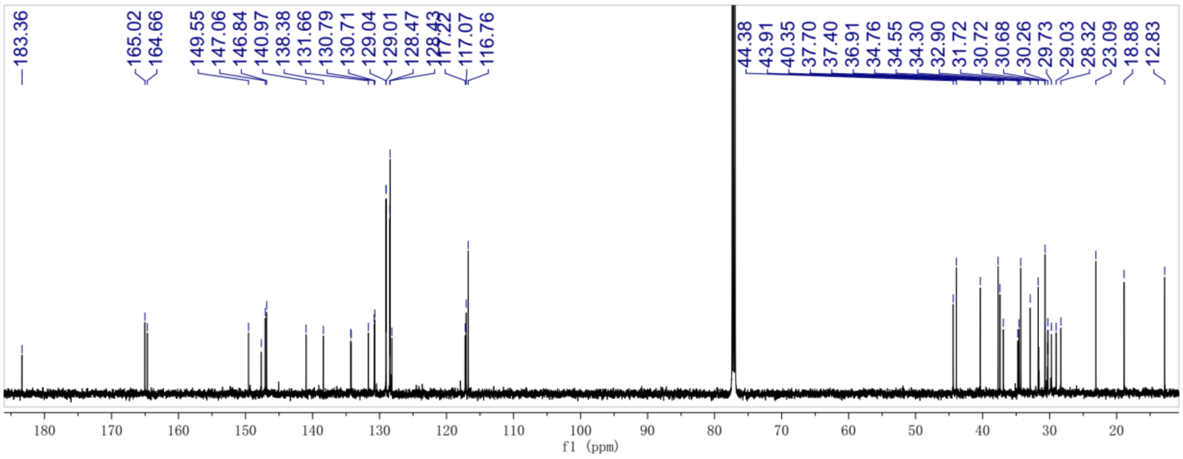

Fig. S39 ^{13}C NMR spectrum of compound **3a** ($CDCl_3$, 150 MHz)

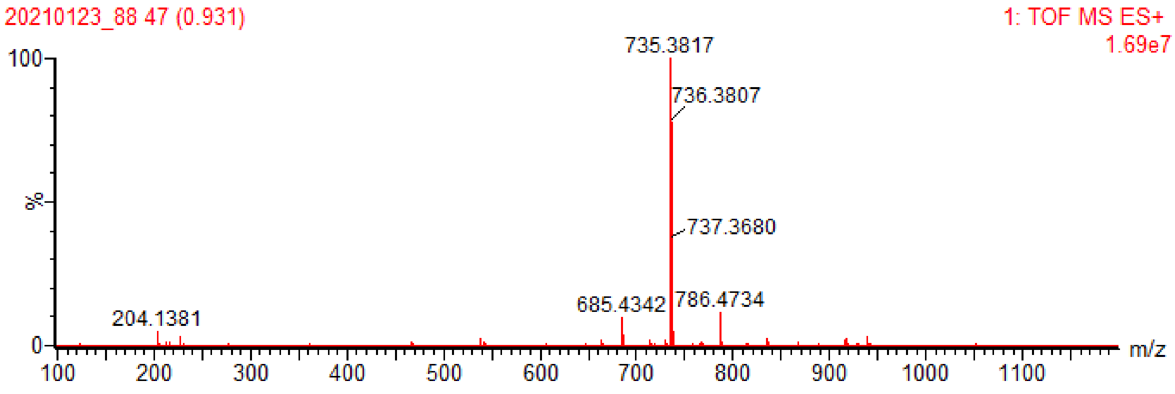

Fig. S40 HR-MS spectrum of compound **3a**

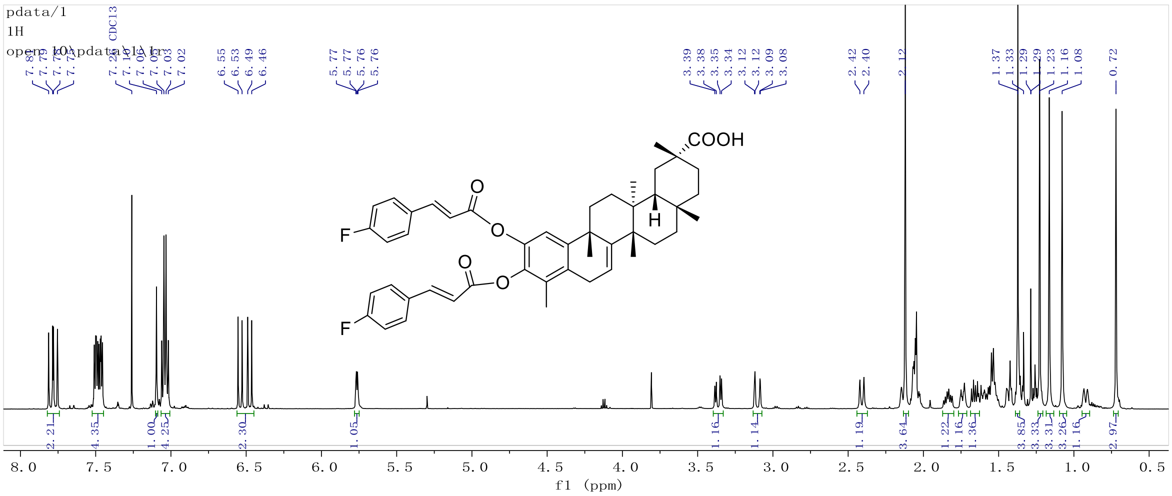

Fig. S41 ^{1}H NMR spectrum of compound **3b** ($CDCl_3$, 600 MHz)

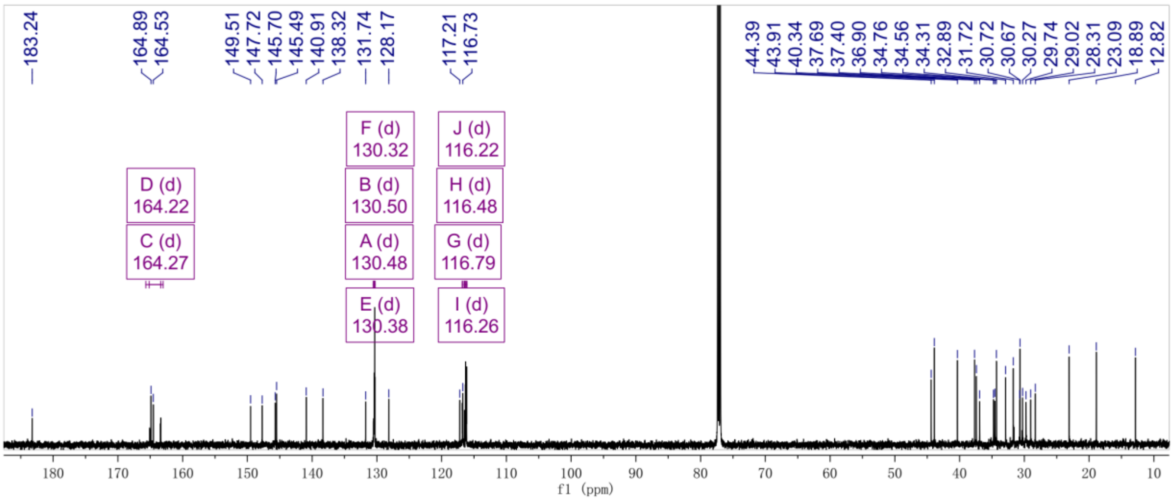

Fig. S42 ^{13}C NMR spectrum of compound **3b** ($CDCl_3$, 150 MHz)

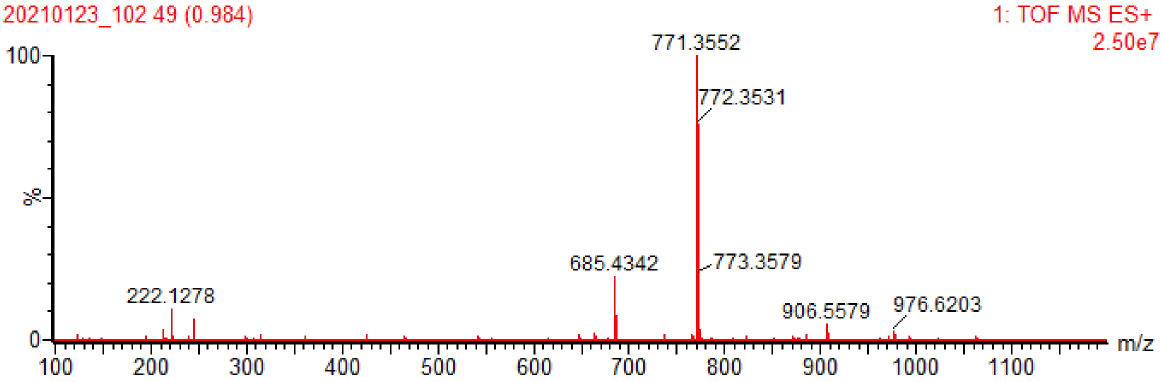

Fig. S43 HR-MS spectrum of compound **3b**

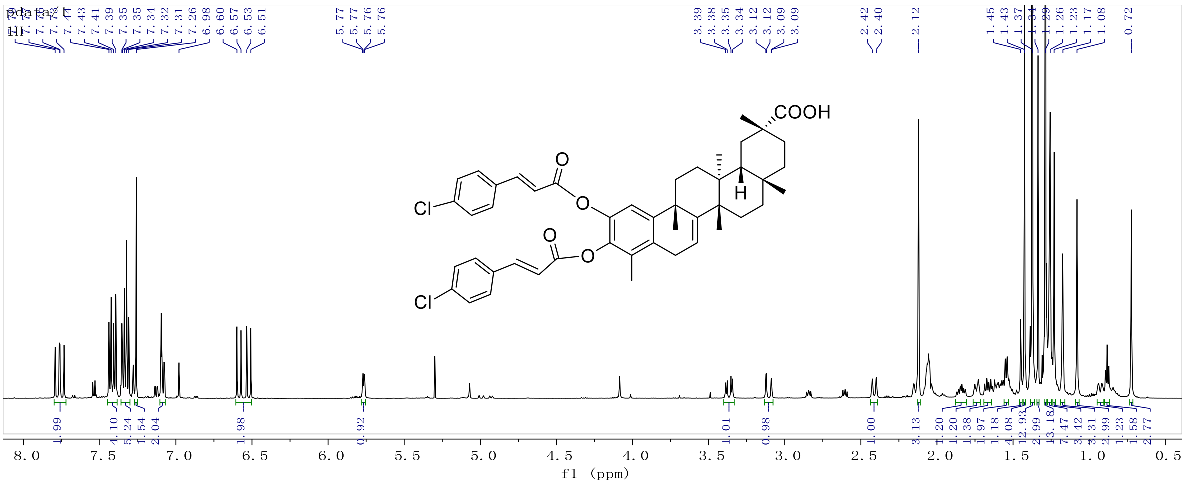

Fig. S44 ^{1}H NMR spectrum of compound **3c** ($CDCl_3$, 600 MHz)

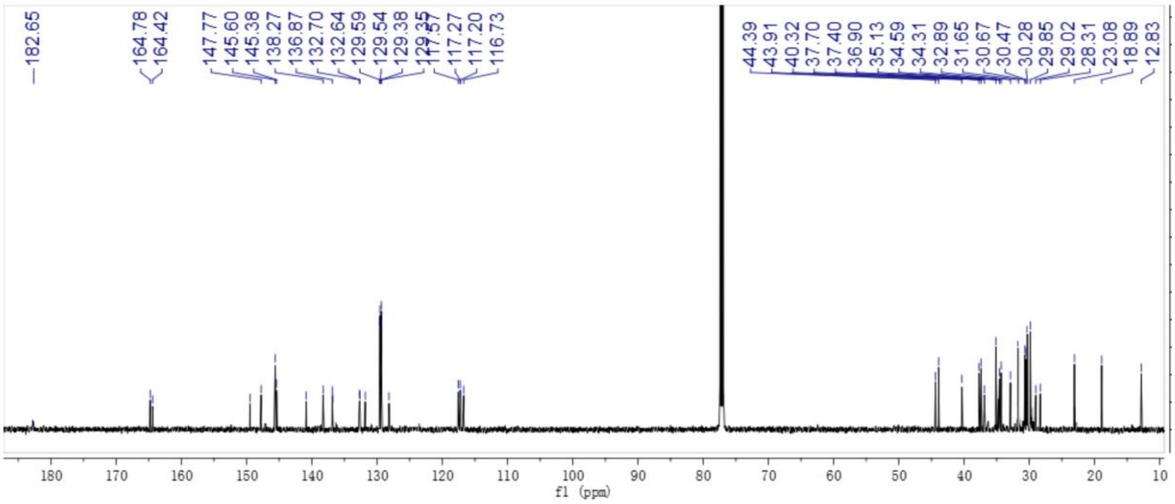

Fig. S45 ^{13}C NMR spectrum of compound **3c** ($CDCl_3$, 150 MHz)

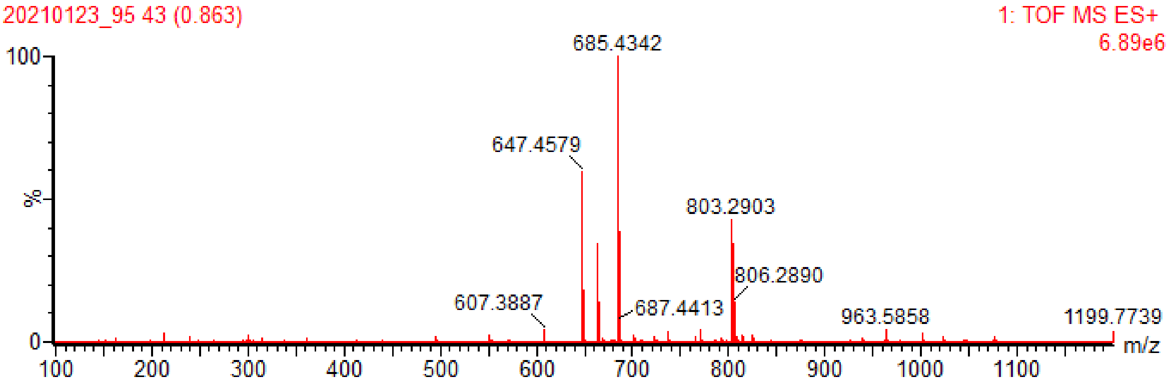

Fig. S46 HR-MS spectrum of compound **3c**

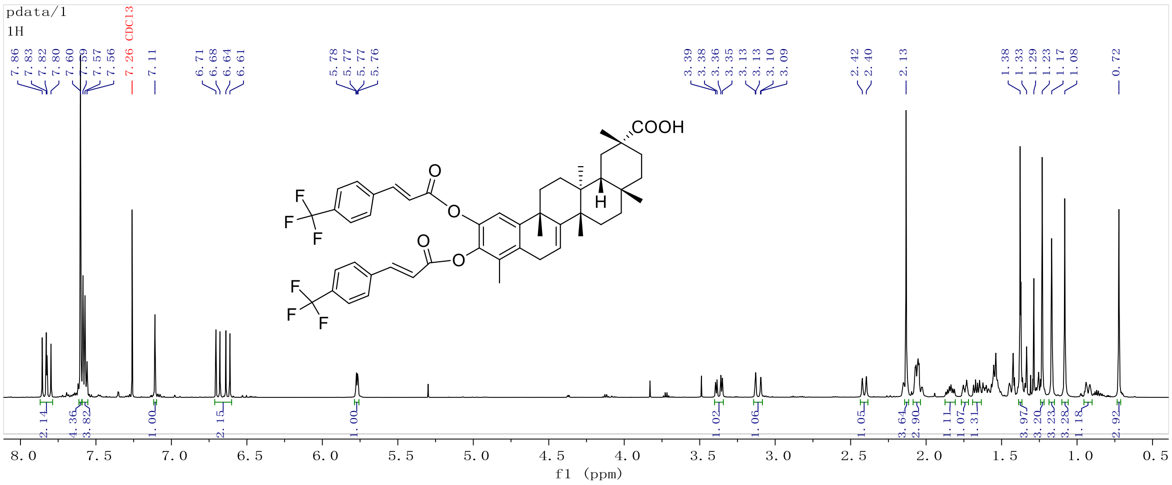

Fig. S47 ^{1}H NMR spectrum of compound **3d** ($CDCl_3$, 600 MHz)

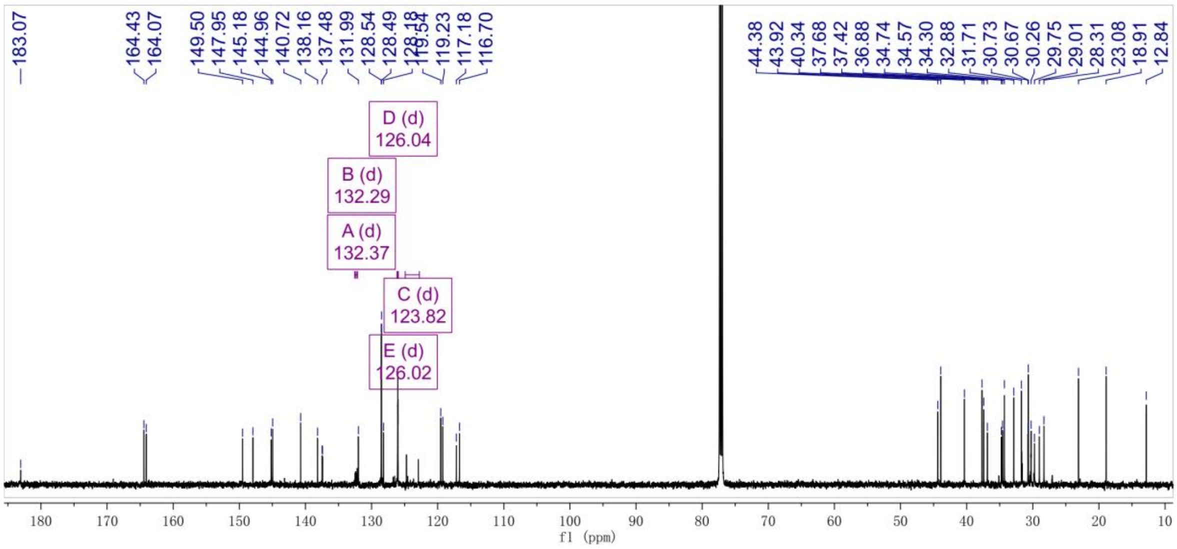

Fig. S48 ^{13}C NMR spectrum of compound **3d** ($CDCl_3$, 150 MHz)

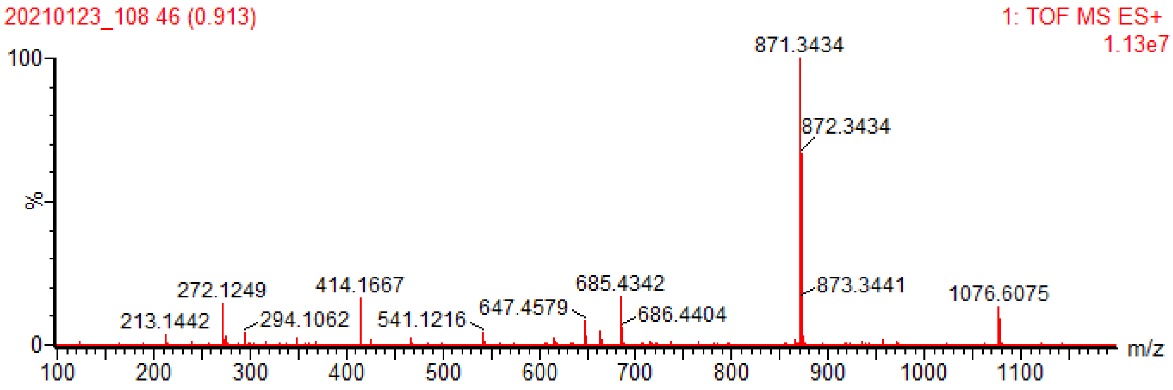

Fig. S49 HR-MS spectrum of compound **3d**

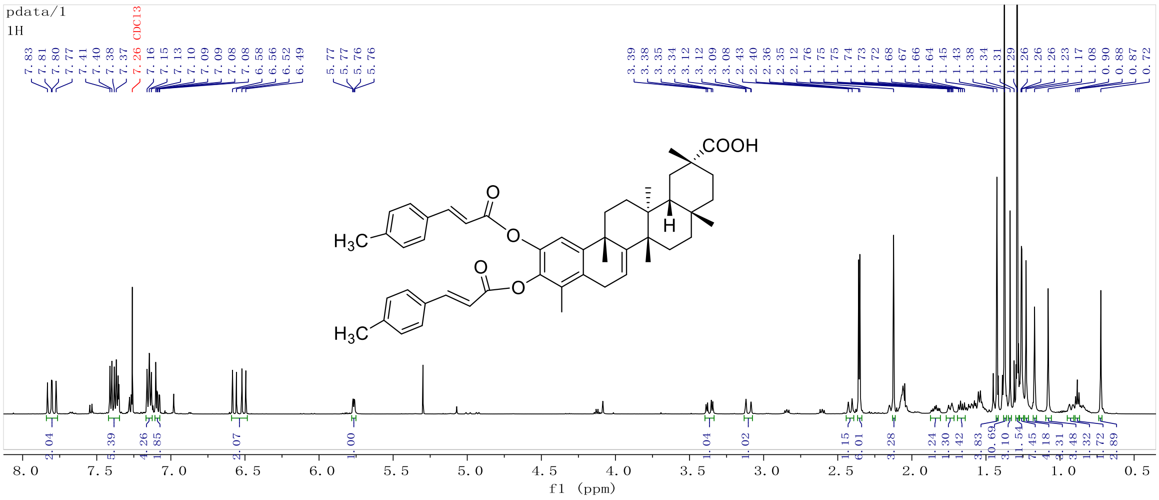

Fig. S50 ^{1}H NMR spectrum of compound **3e** ($CDCl_3$, 600 MHz)

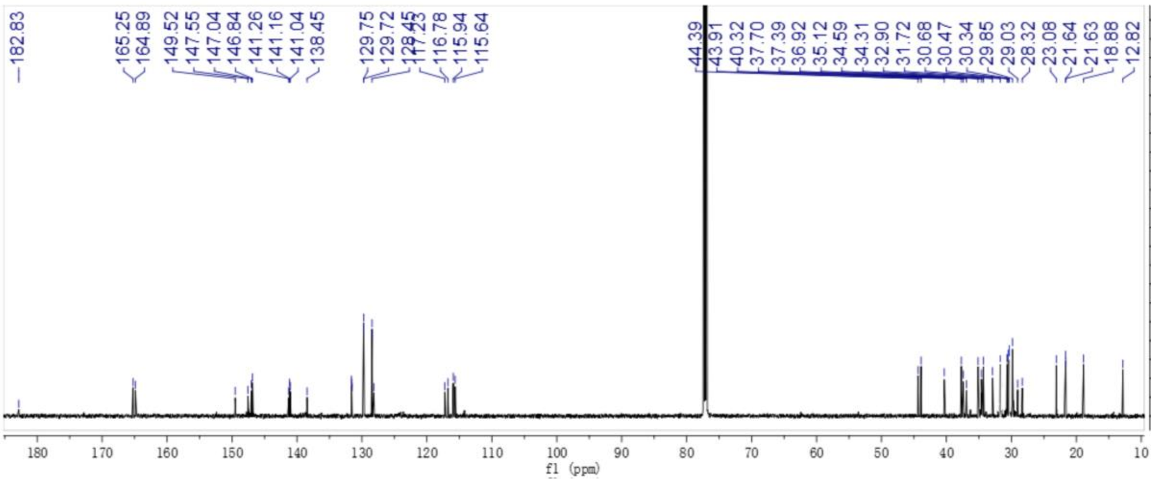

Fig. S51 ^{13}C NMR spectrum of compound **3e** ($CDCl_3$, 150 MHz)

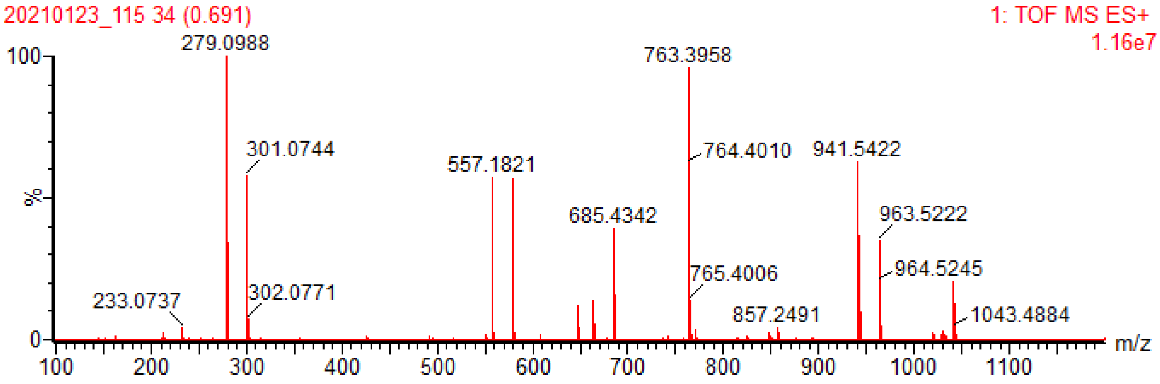

Fig. S52 HR-MS spectrum of compound **3e**

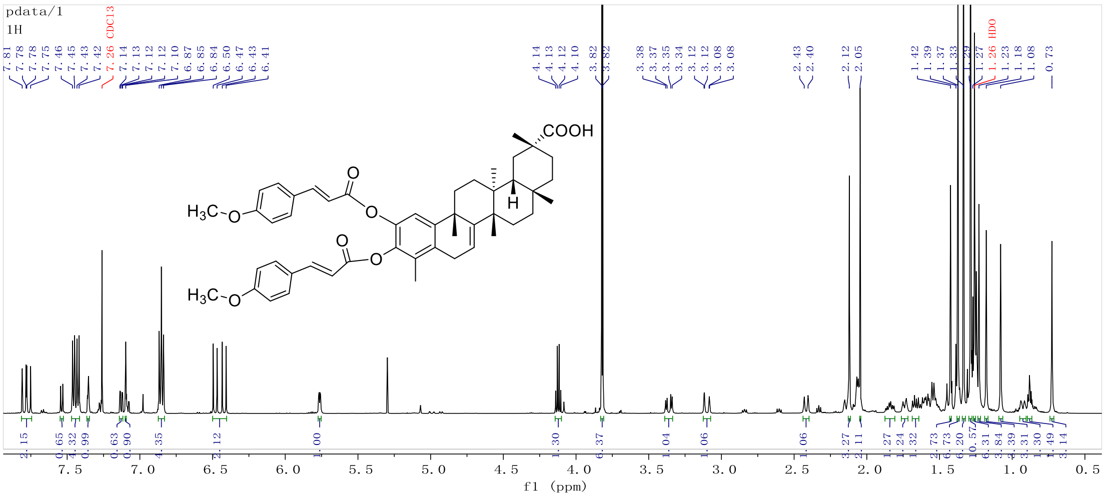

Fig. S53 ^{1}H NMR spectrum of compound **3f** ($CDCl_3$, 600 MHz)

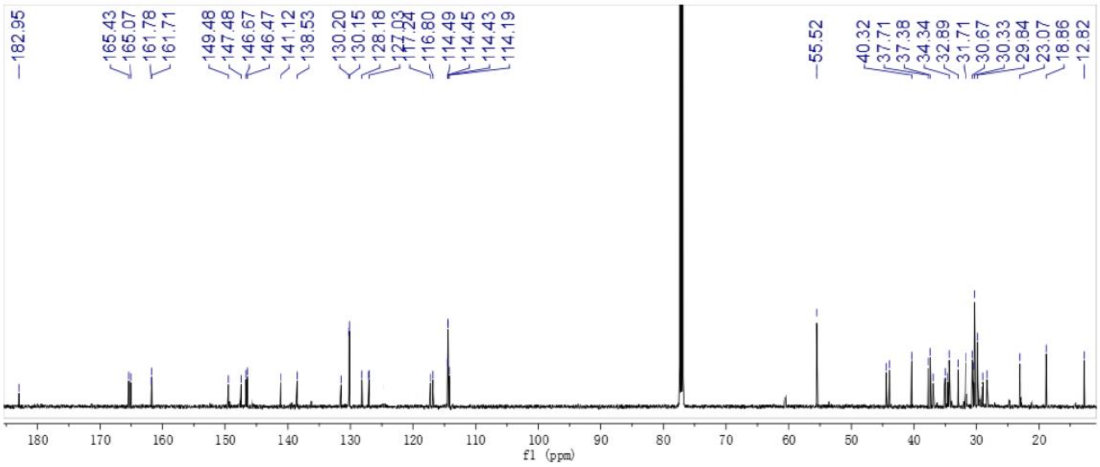

Fig. S54 ^{13}C NMR spectrum of compound **3f** ($CDCl_3$, 150 MHz)

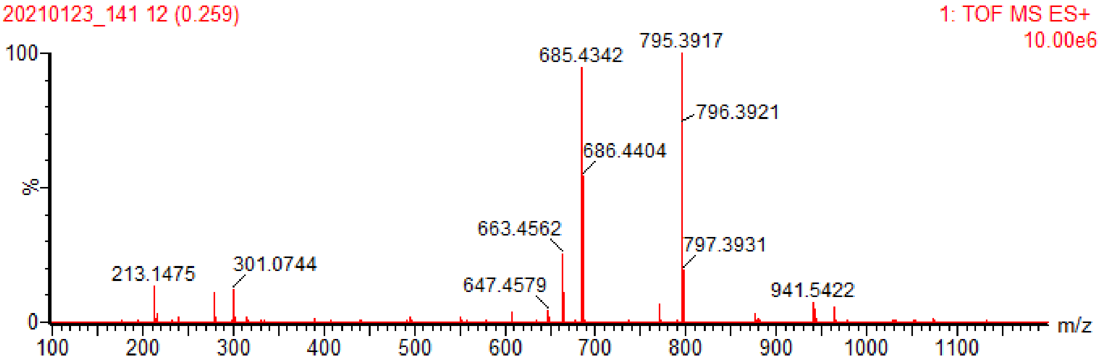

Fig. S55 HR-MS spectrum of compound **3f**

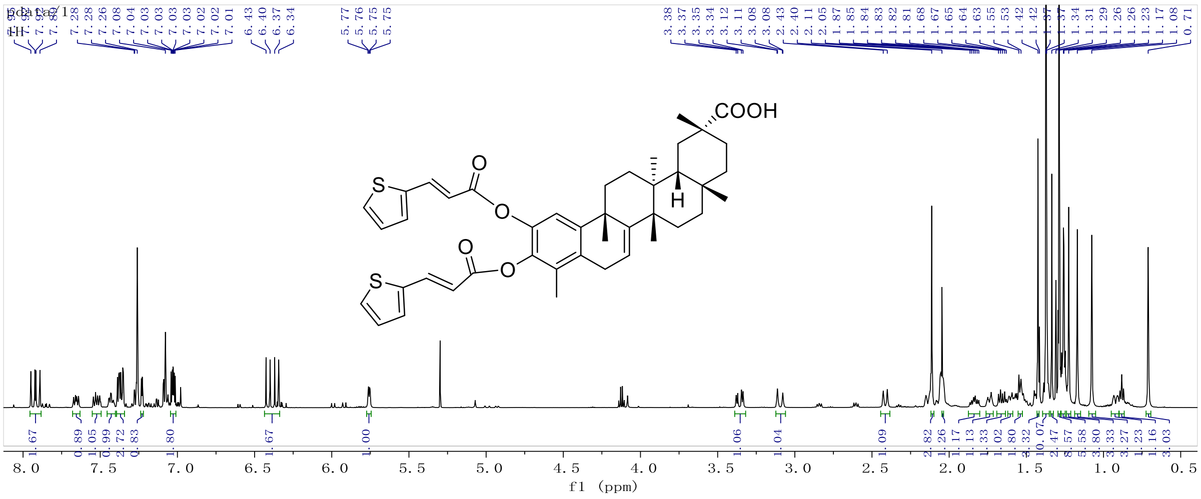

Fig. S56 ^{1}H NMR spectrum of compound **3g** ($CDCl_3$, 600 MHz)

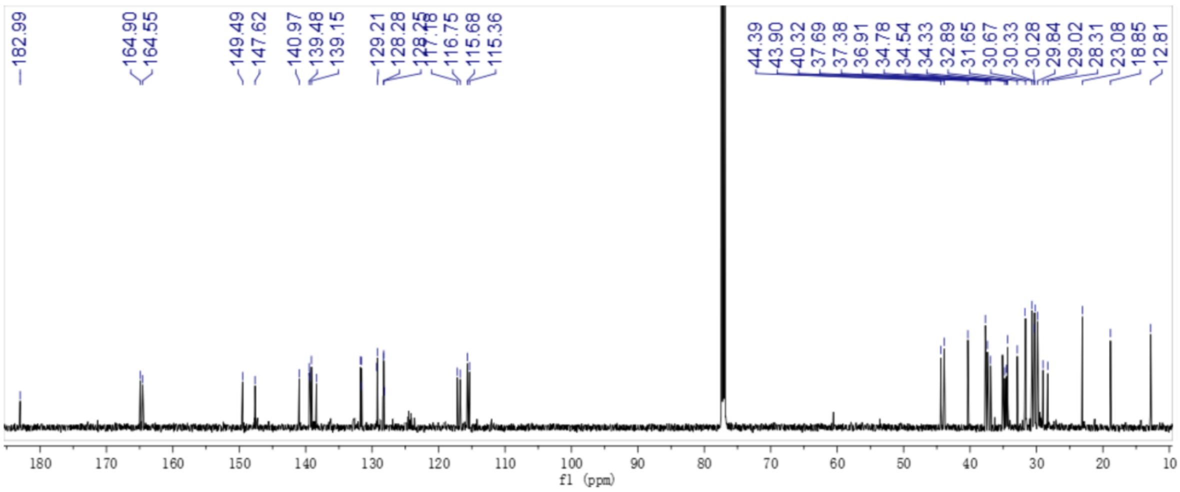

Fig. S57 ^{13}C NMR spectrum of compound **3g** ($CDCl_3$, 150 MHz)

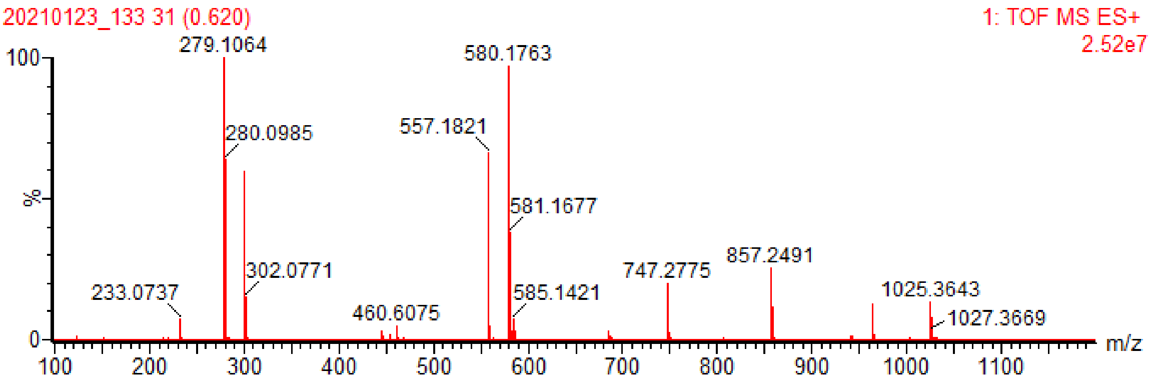

Fig. S58 HR-MS spectrum of compound **3g**

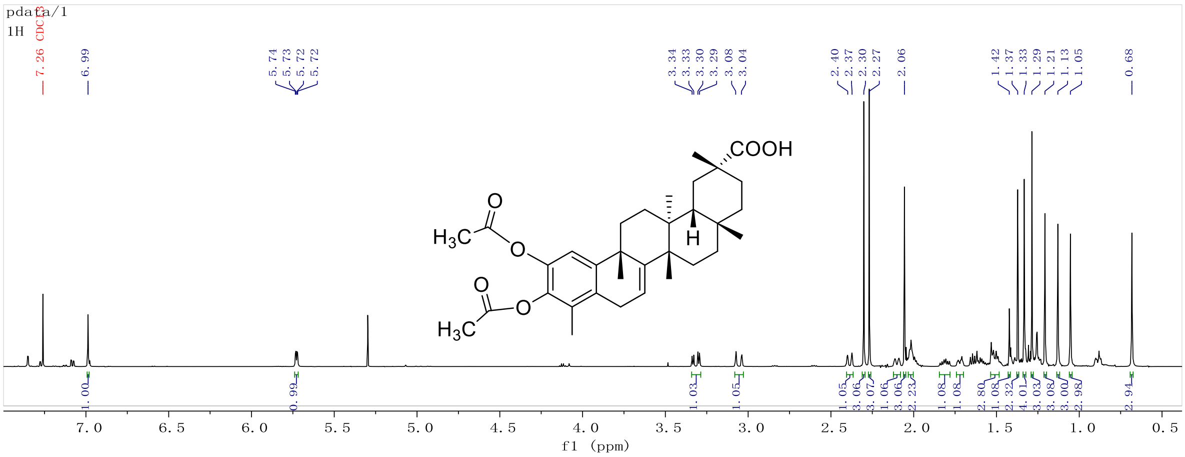

Fig. S59 ^{1}H NMR spectrum of compound **3h** ($CDCl_3$, 600 MHz)

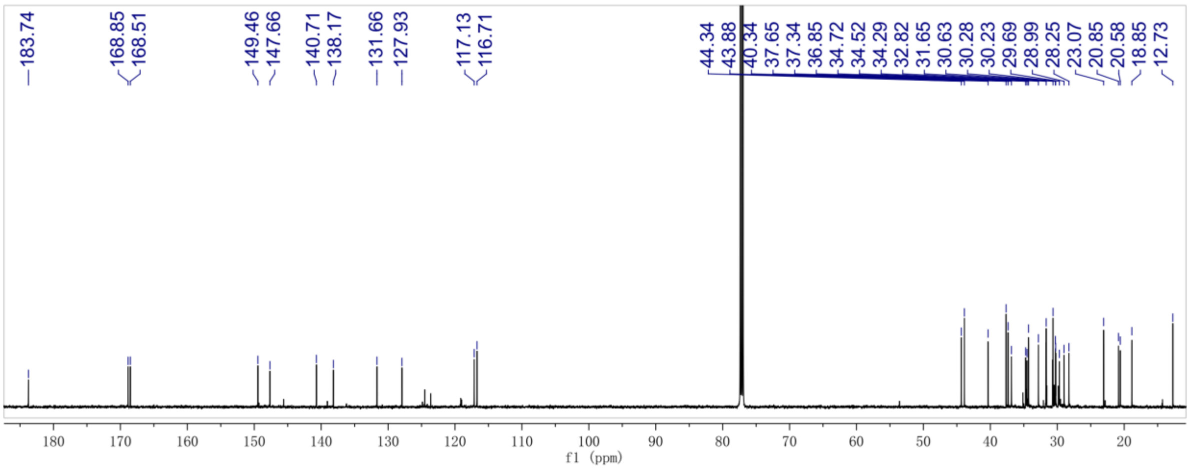

Fig. S60 ^{13}C NMR spectrum of compound **3h** ($CDCl_3$, 150 MHz)

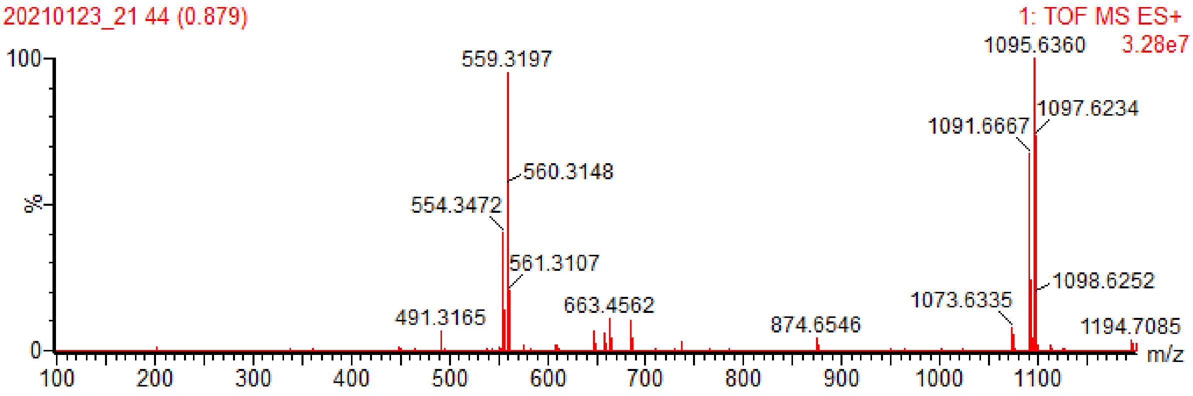

Fig. 61 HR-MS spectrum of compound **3h**

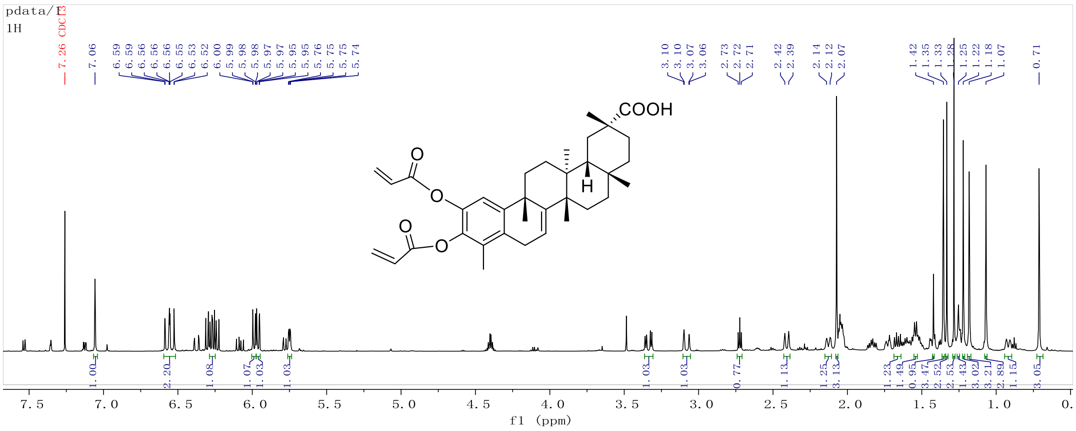

Fig. S62 ^{1}H NMR spectrum of compound **3i** ($CDCl_3$, 600 MHz)

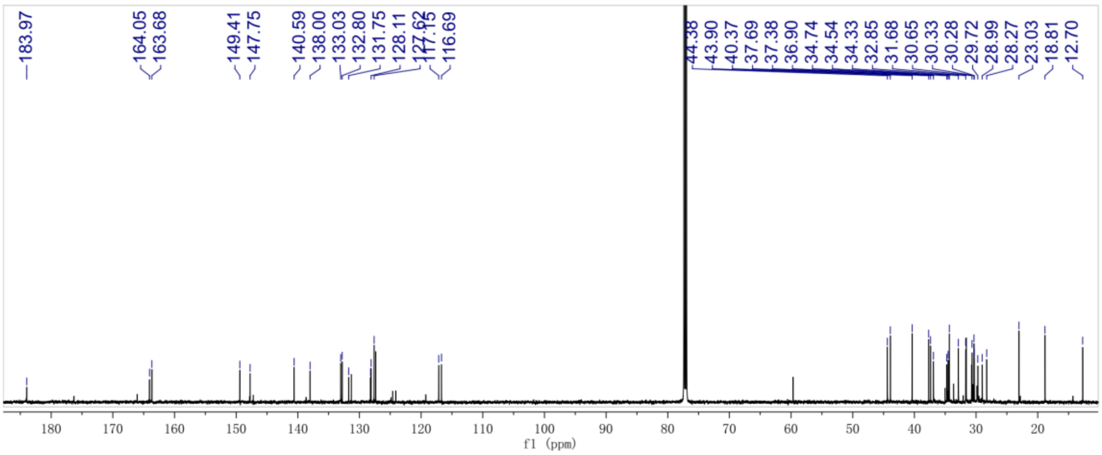

Fig. S63 ^{13}C NMR spectrum of compound **3i** ($CDCl_3$, 150 MHz)

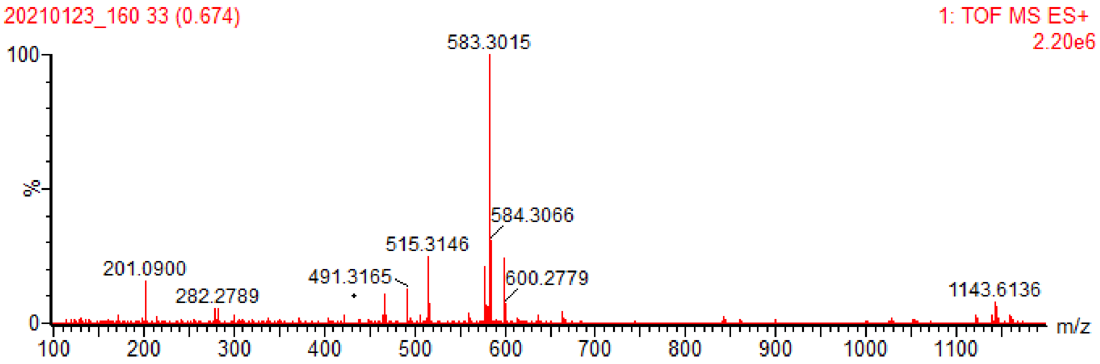

Fig. S64HR-MS spectrum of compound **3i**

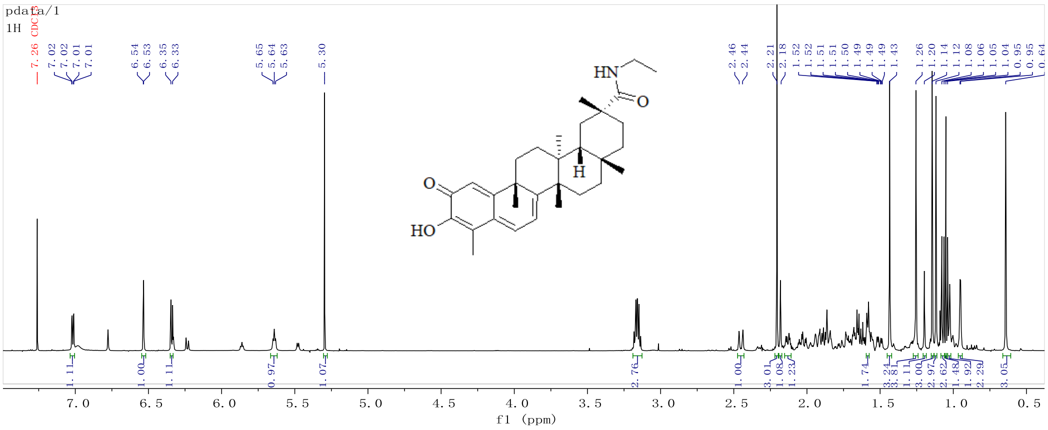

Fig. S65 ^{1}H NMR spectrum of compound **4a** ($CDCl_3$, 600 MHz)

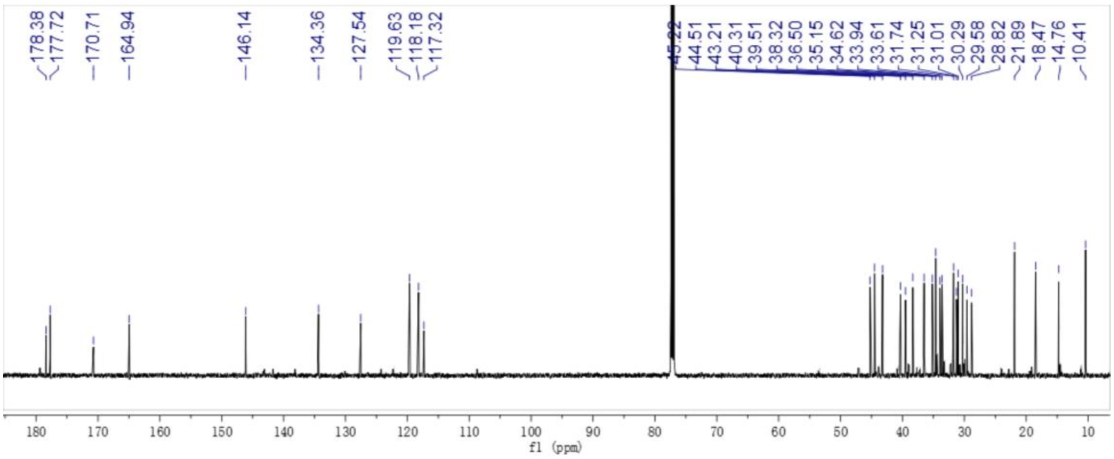

Fig. S66 ^{13}C NMR spectrum of compound **4a** ($CDCl_3$, 150 MHz)

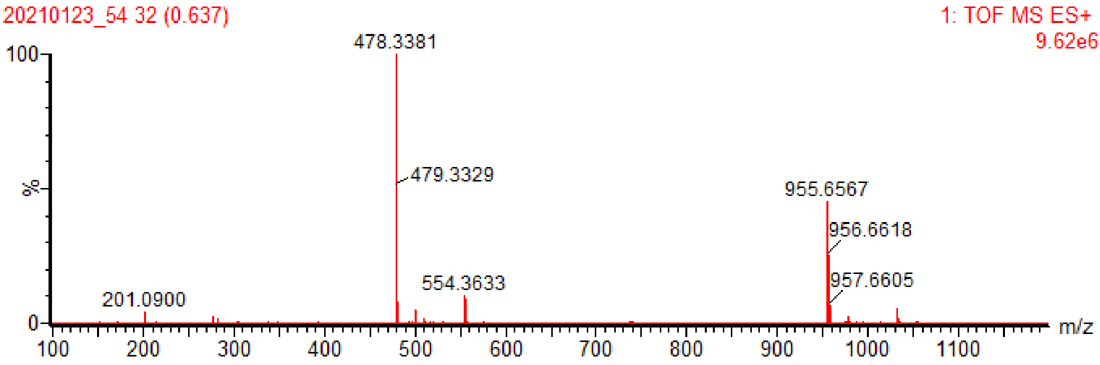

Fig. S67 HR-MS spectrum of compound **4a**

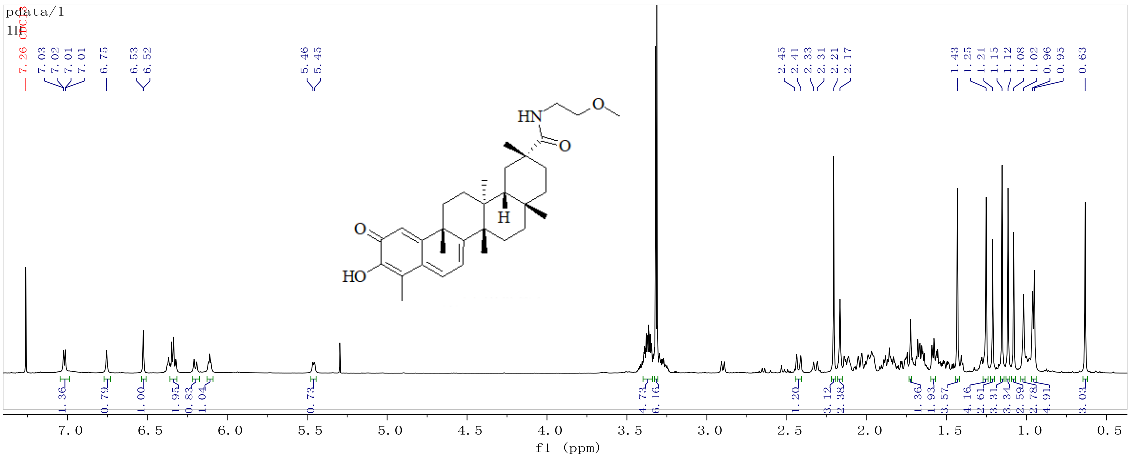

Fig. S68 ^{1}H NMR spectrum of compound **4b** ($CDCl_3$, 600 MHz)

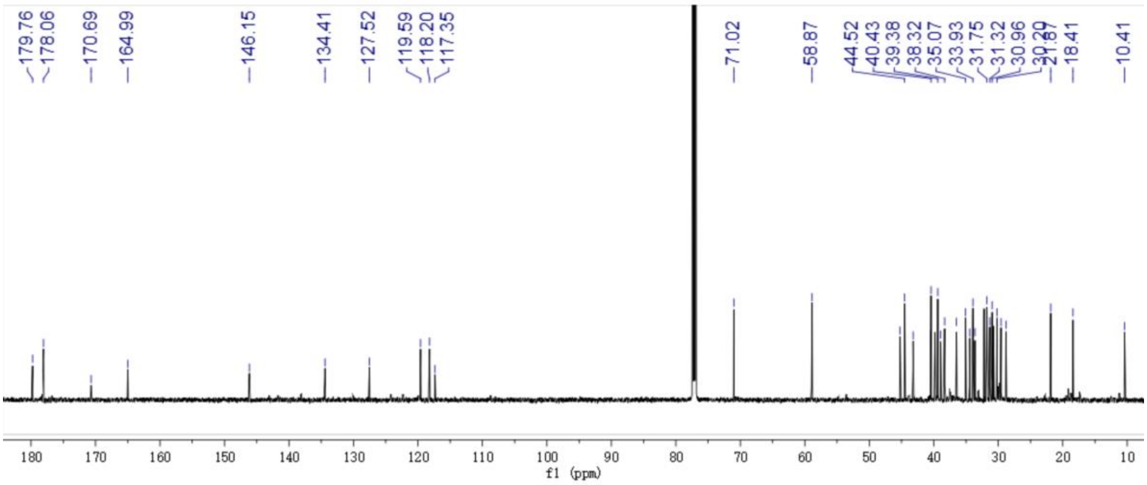

Fig. S69 ^{13}C NMR spectrum of compound **4b** ($CDCl_3$, 150 MHz)

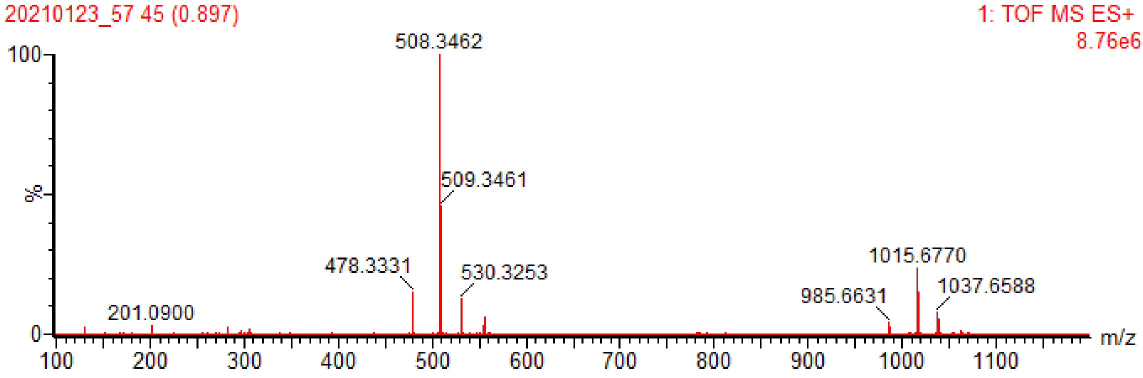

Fig. S70 HR-MS spectrum of compound **4b**

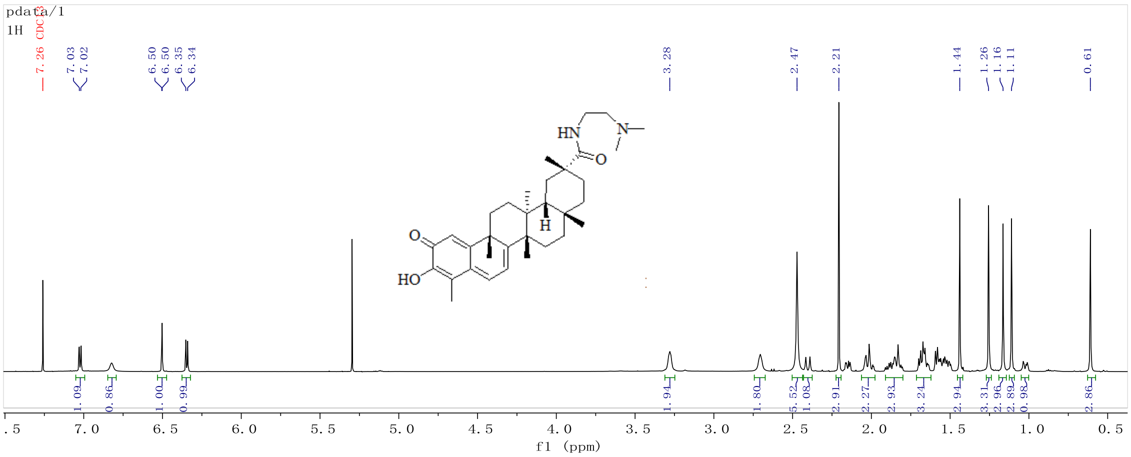

Fig. S71 ^{1}H NMR spectrum of compound **4c** ($CDCl_3$, 600 MHz)

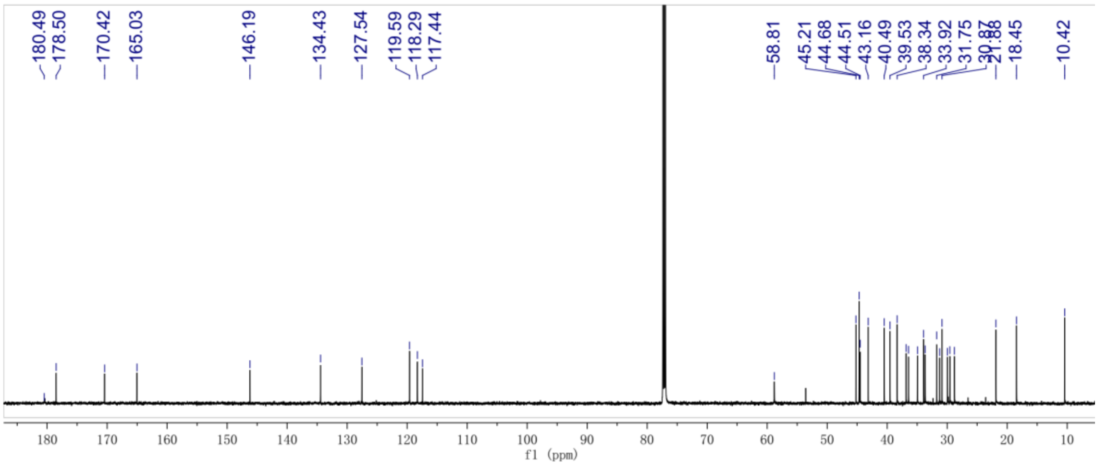

Fig. S72 ^{13}C NMR spectrum of compound **4c** ($CDCl_3$, 150 MHz)

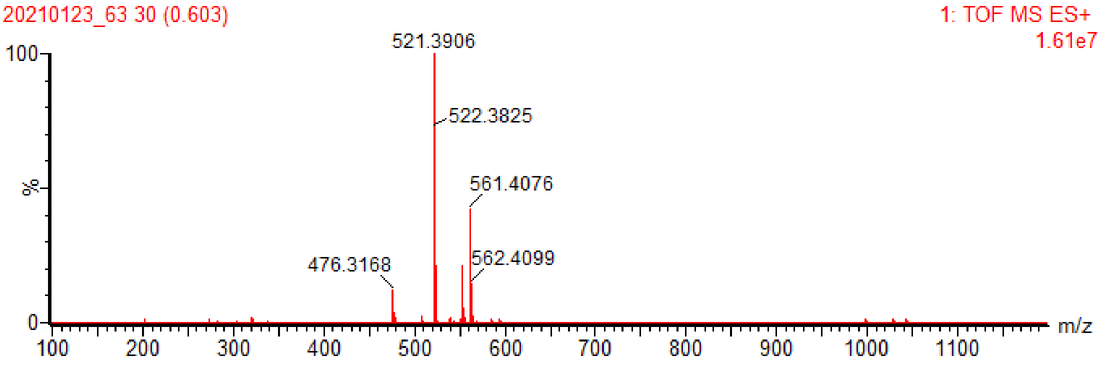

Fig. S73 HR-MS spectrum of compound **4c**

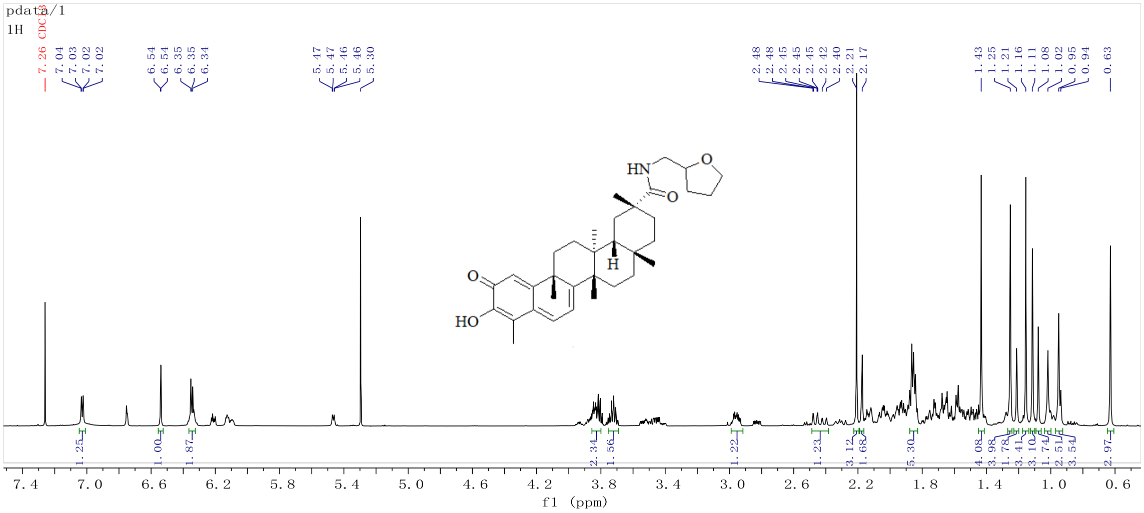

Fig. S74 ^{1}H NMR spectrum of compound **4d** ($CDCl_3$, 600 MHz)

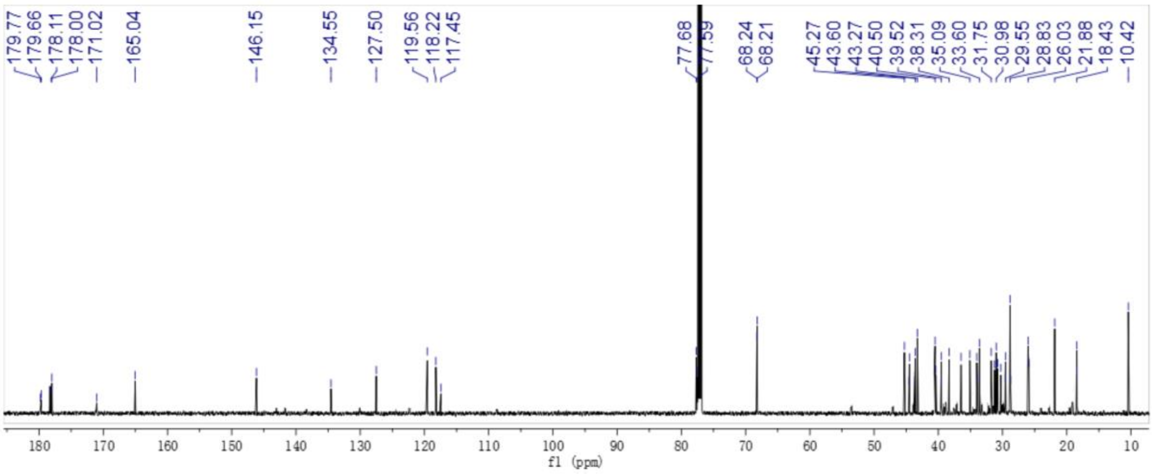

Fig. S75 ^{13}C NMR spectrum of compound **4d** ($CDCl_3$, 150 MHz)

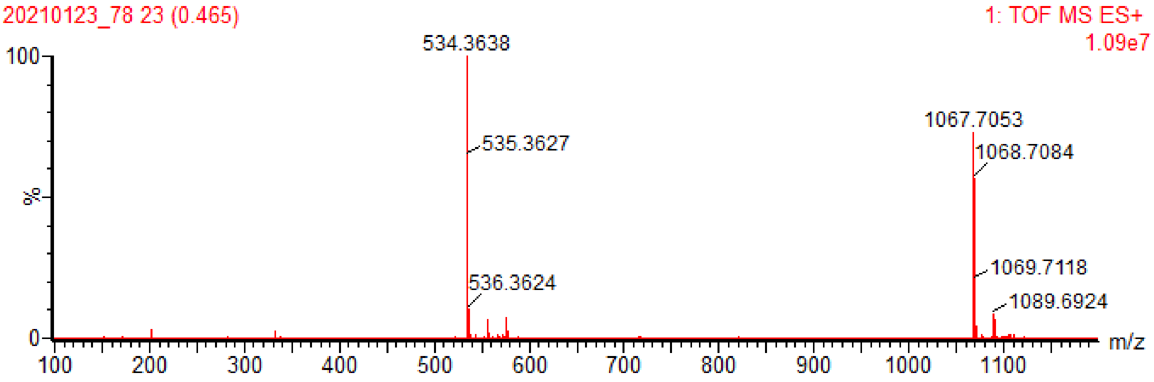

Fig. S76 HR-MS spectrum of compound **4d**

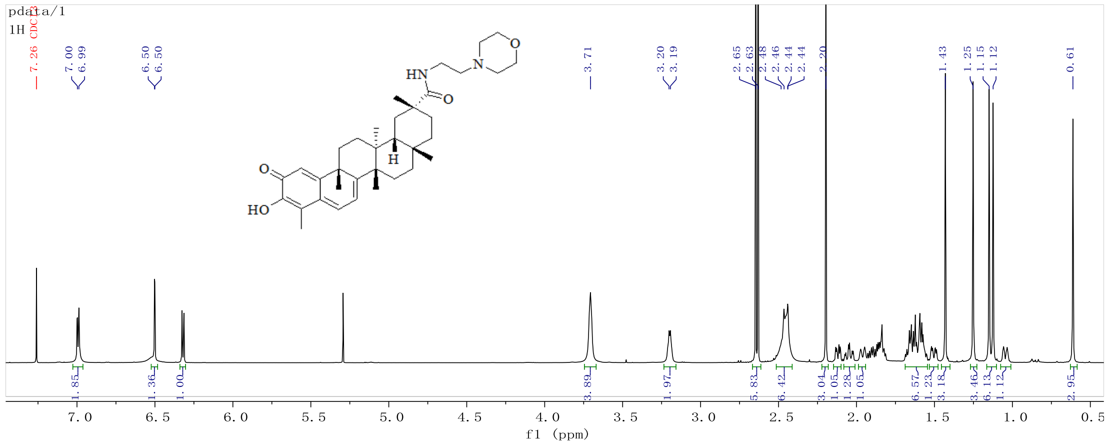

Fig. S77 ^{1}H NMR spectrum of compound **4e** ($CDCl_3$, 600 MHz)

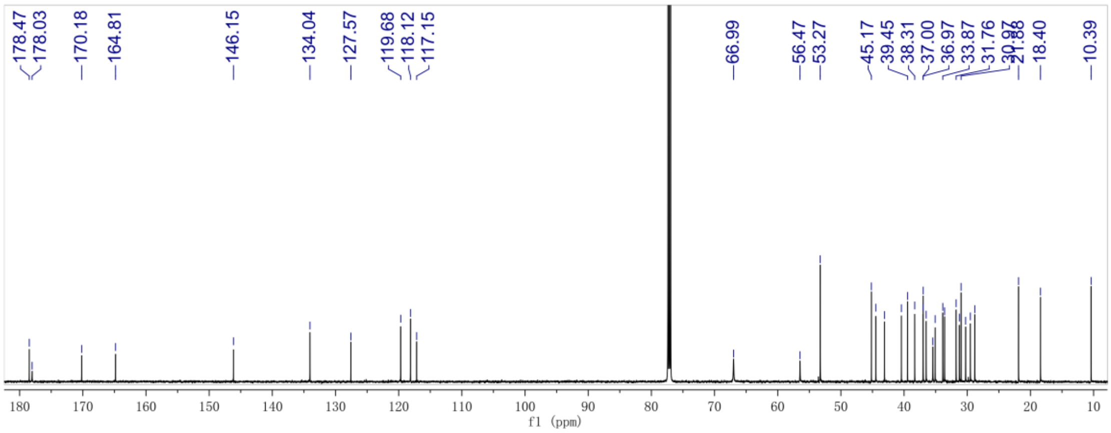

Fig. S78 ^{13}C NMR spectrum of compound **4e** ($CDCl_3$, 150 MHz)

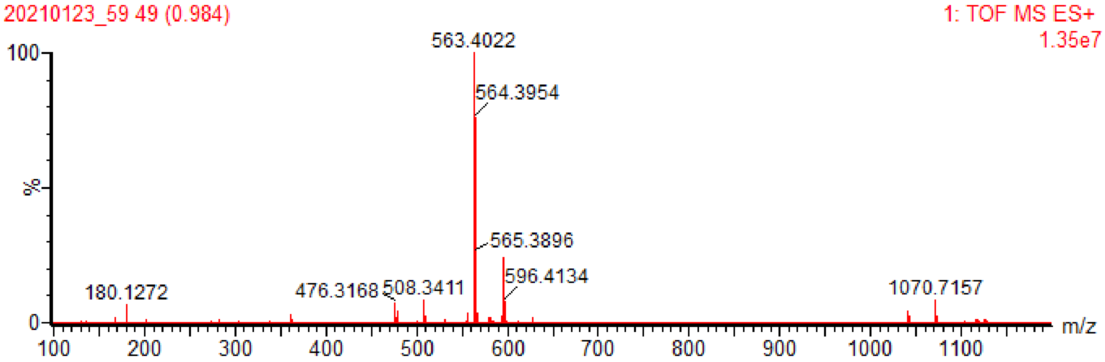

Fig. S79 HR-MS spectrum of compound **4e**

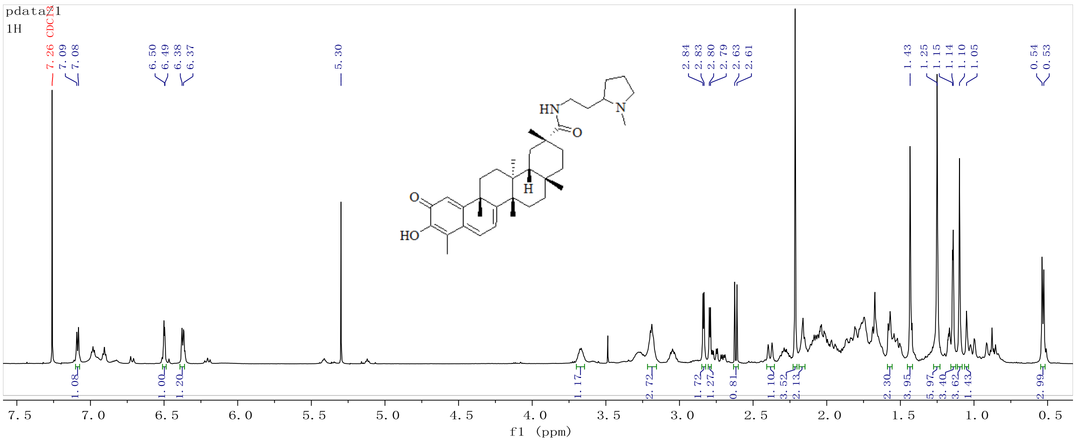

Fig. S80 ^{1}H NMR spectrum of compound **4f** ($CDCl_3$, 600 MHz)

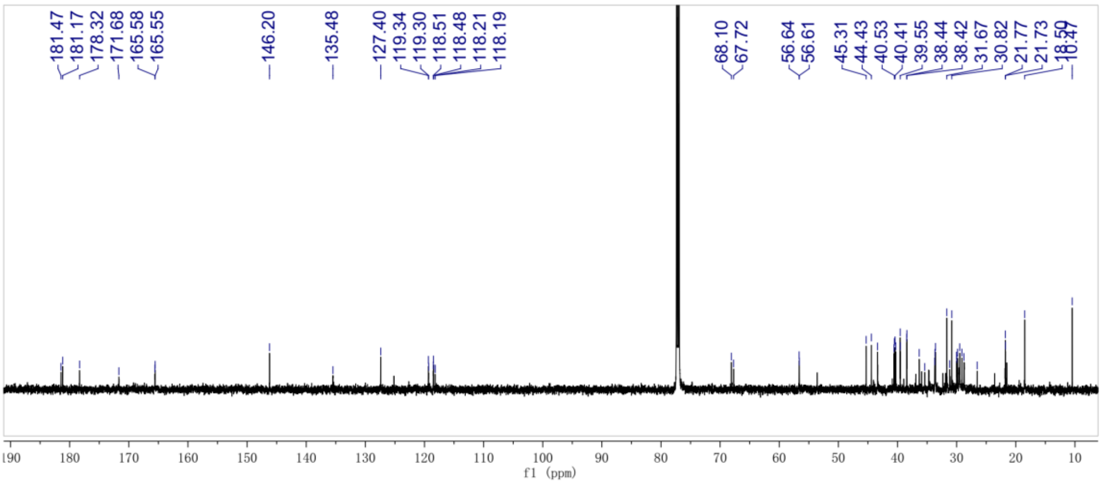

Fig. S81 ^{13}C NMR spectrum of compound **4f** ($CDCl_3$, 150 MHz)

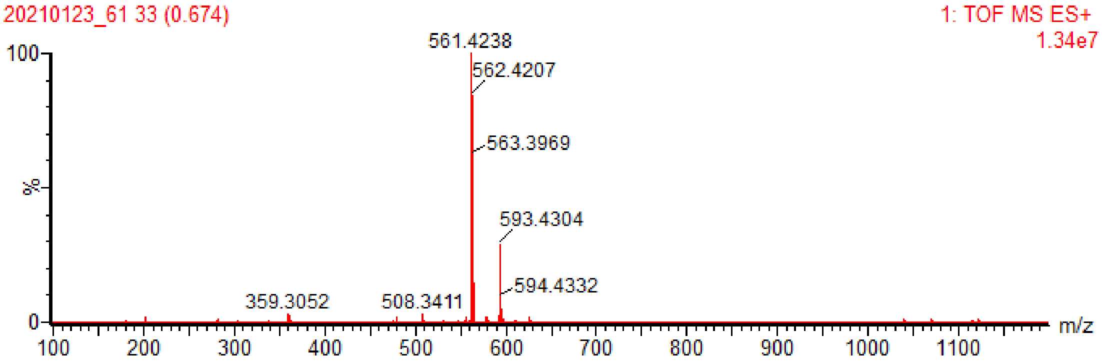

Fig. S82 HR-MS spectrum of compound **4f**

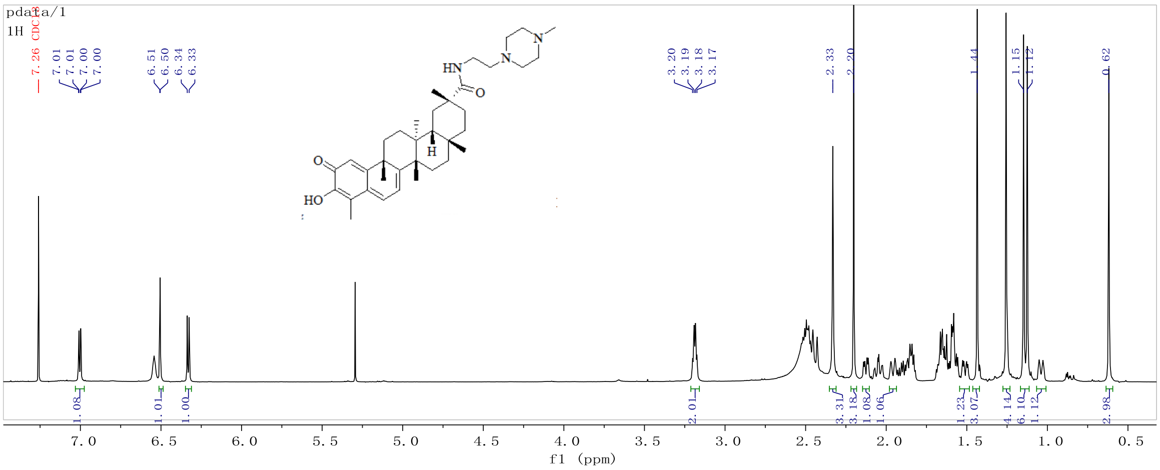

Fig. S83 ^{1}H NMR spectrum of compound **4g** ($CDCl_3$, 600 MHz)

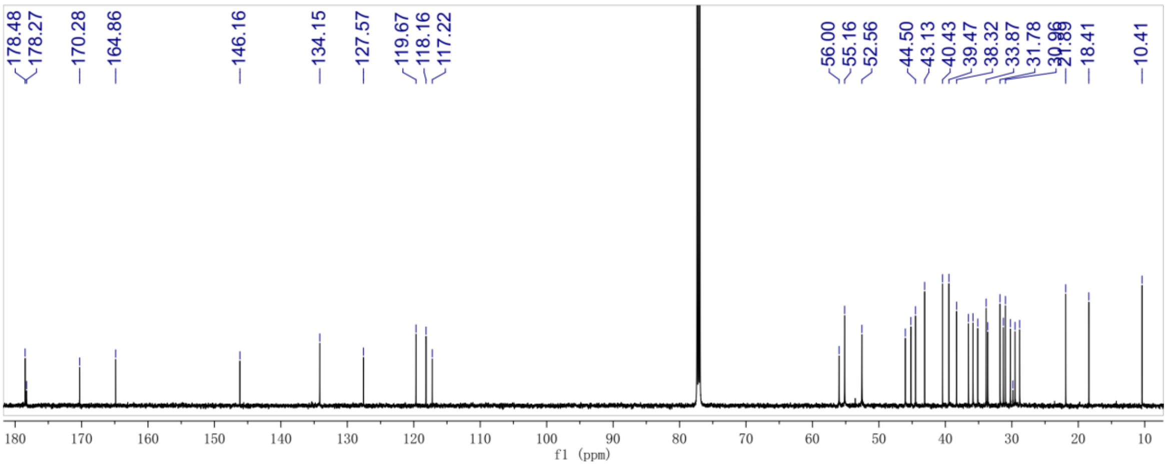

Fig. S84 ^{13}C NMR spectrum of compound **4g** ($CDCl_3$, 150 MHz)

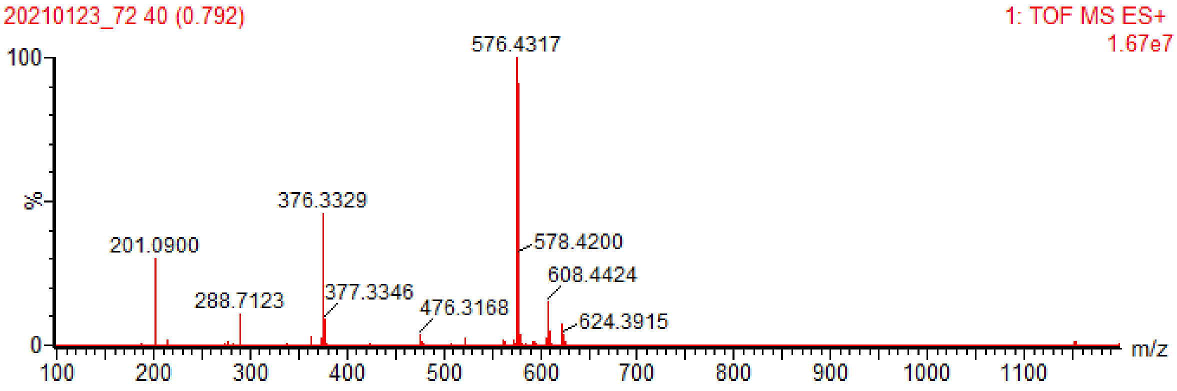

Fig. S85 HR-MS spectrum of compound **4g**

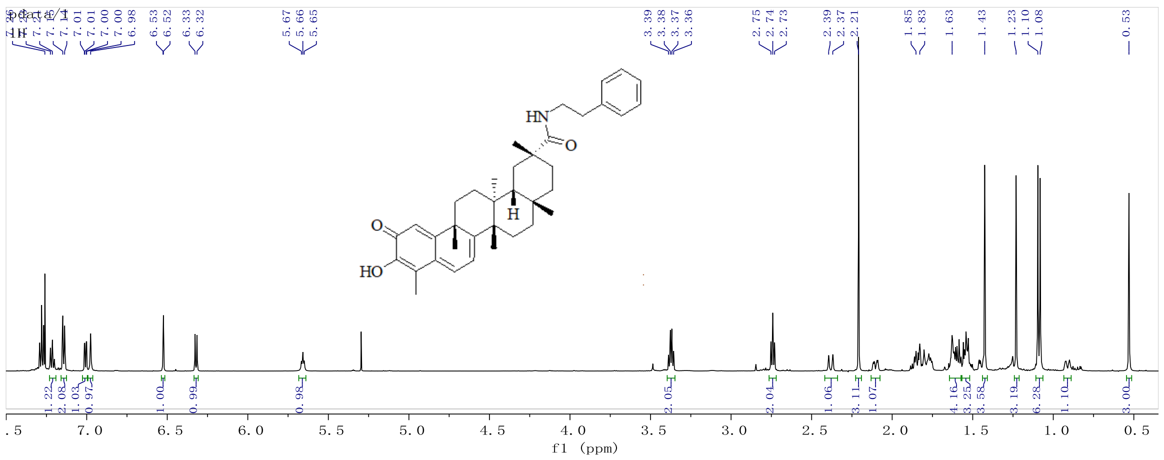

Fig. S86 ^{1}H NMR spectrum of compound **4h** ($CDCl_3$, 600 MHz)

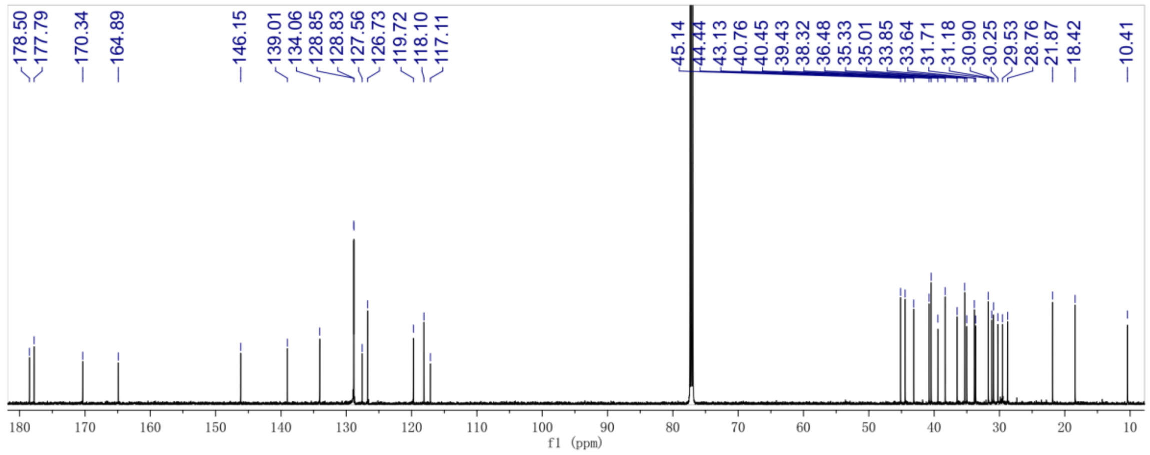

Fig. S87 ^{13}C NMR spectrum of compound **4g** ($CDCl_3$, 150 MHz)

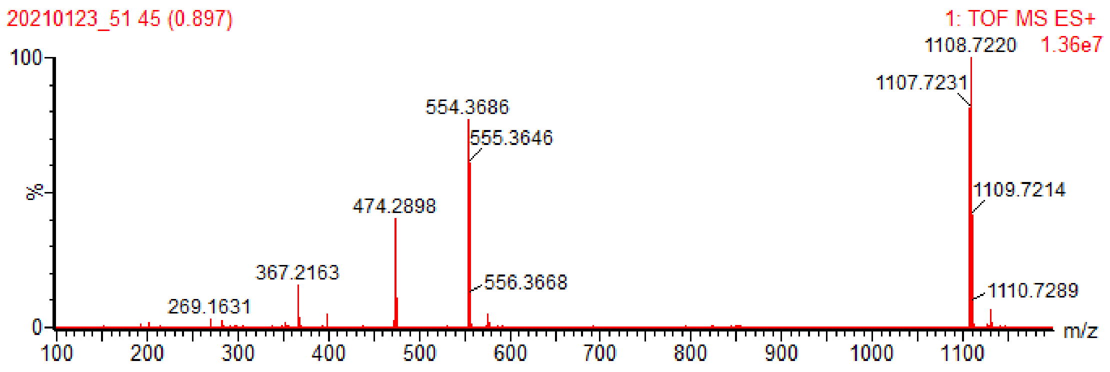

Fig. S88 HR-MS spectrum of compound **4h**

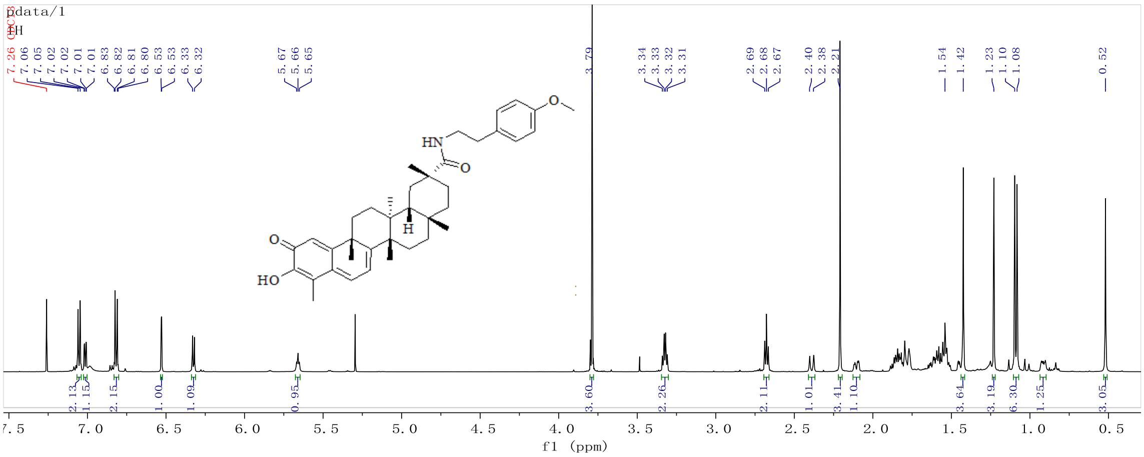

Fig. S89 ^{1}H NMR spectrum of compound **4i** ($CDCl_3$, 600 MHz)

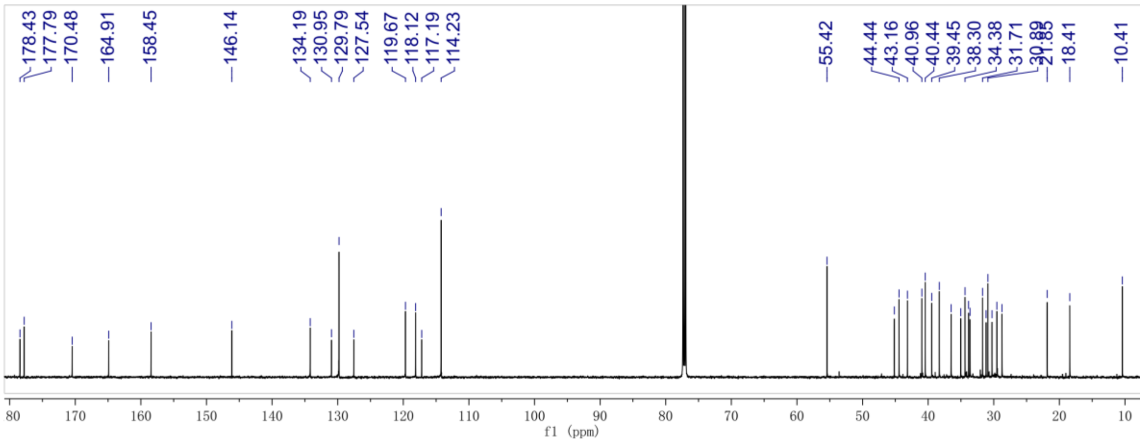

Fig. S90 ^{13}C NMR spectrum of compound **4i** ($CDCl_3$, 150 MHz)

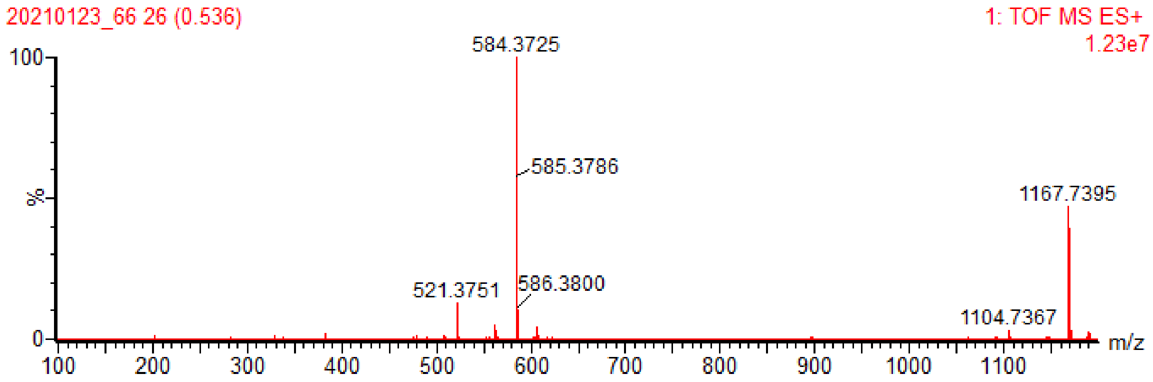

Fig. S91 HR-MS spectrum of compound **4i**

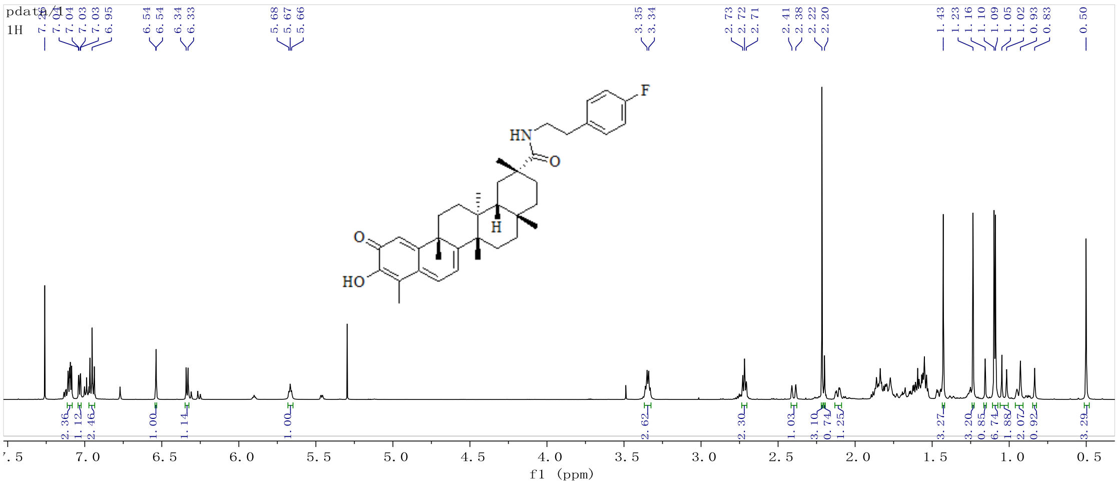

Fig. S92 ^{1}H NMR spectrum of compound **4j** ($CDCl_3$, 600 MHz)

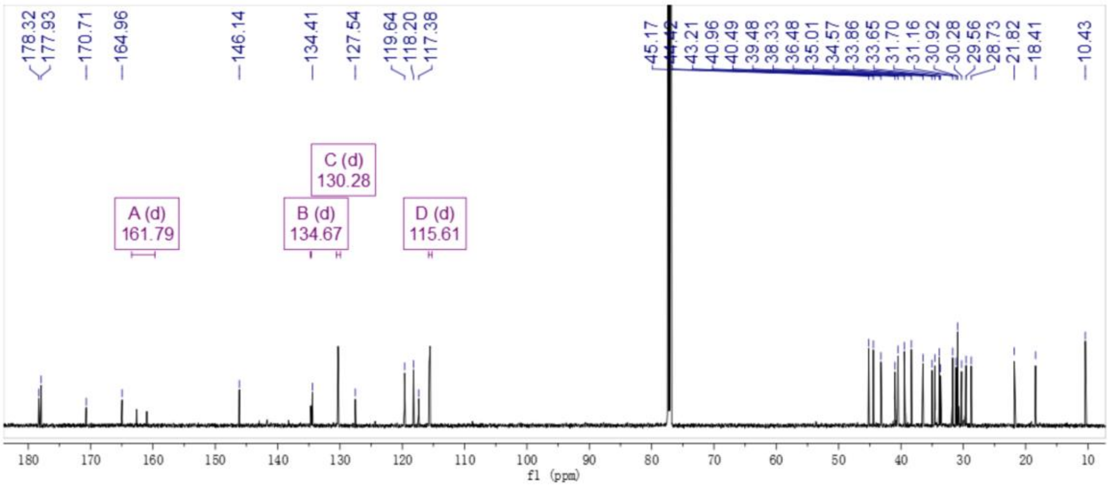

Fig. S93 ^{13}C NMR spectrum of compound **4j** ($CDCl_3$, 150 MHz)

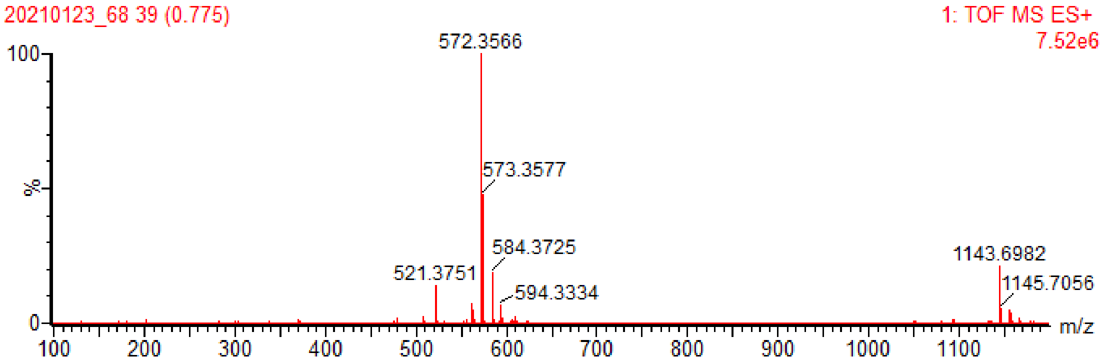

Fig. S94 HR-MS spectrum of compound **4j**

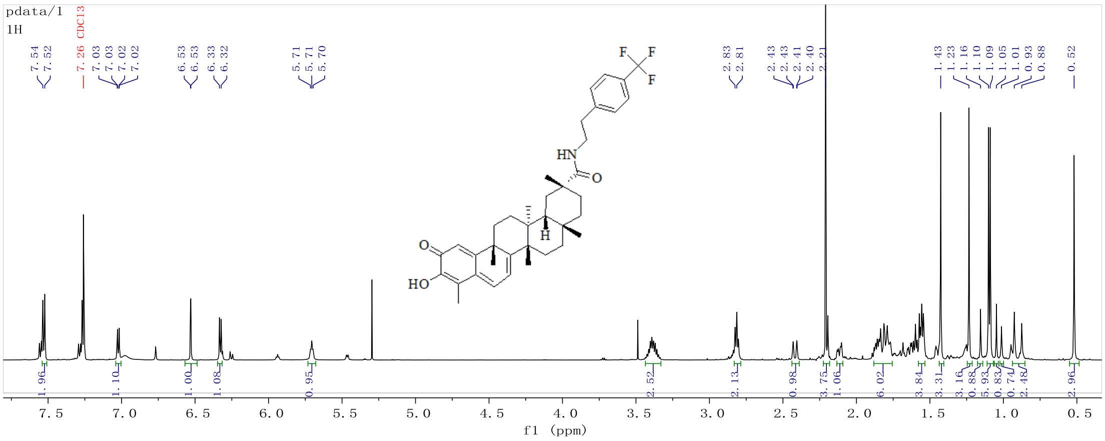

Fig. S95 ^{1}H NMR spectrum of compound **4k** ($CDCl_3$, 600 MHz)

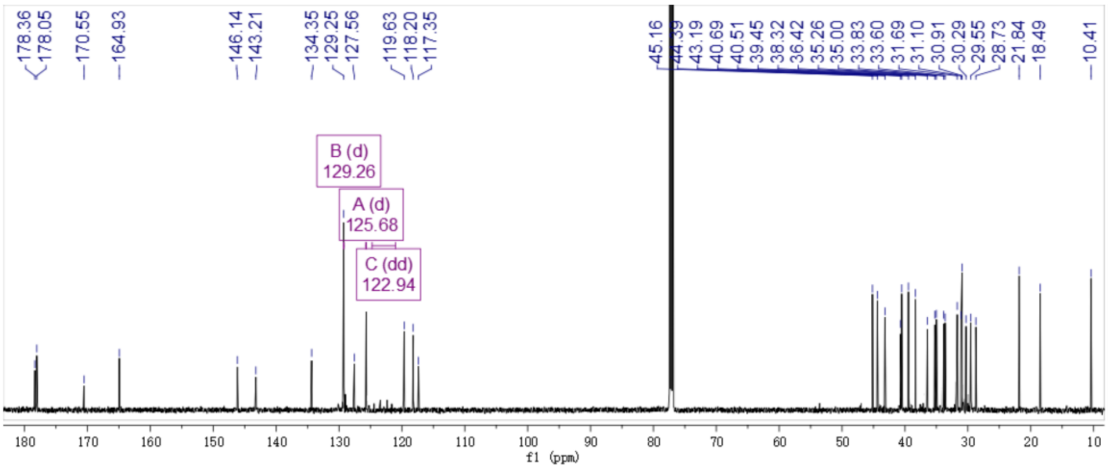

Fig. S96 ^{13}C NMR spectrum of compound **4k** ($CDCl_3$, 150 MHz)

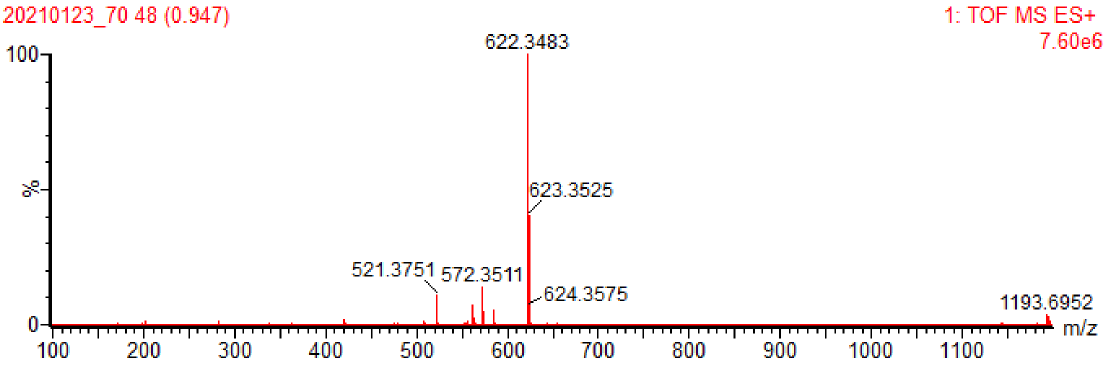

Fig. S97 HR-MS spectrum of compound **4k**

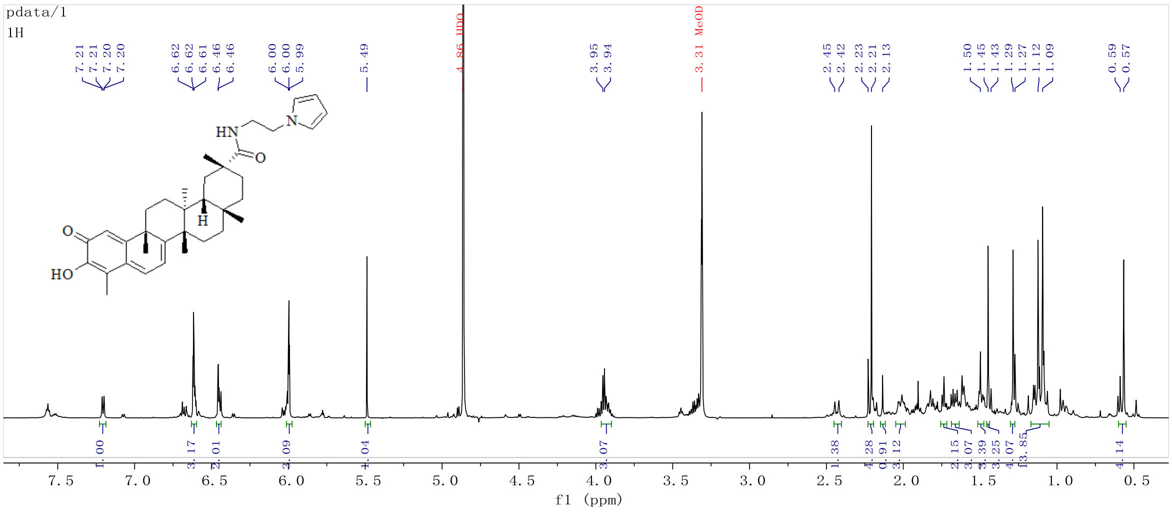

Fig. S98 ^{1}H NMR spectrum of compound **4l** (MeOD, 600 MHz)

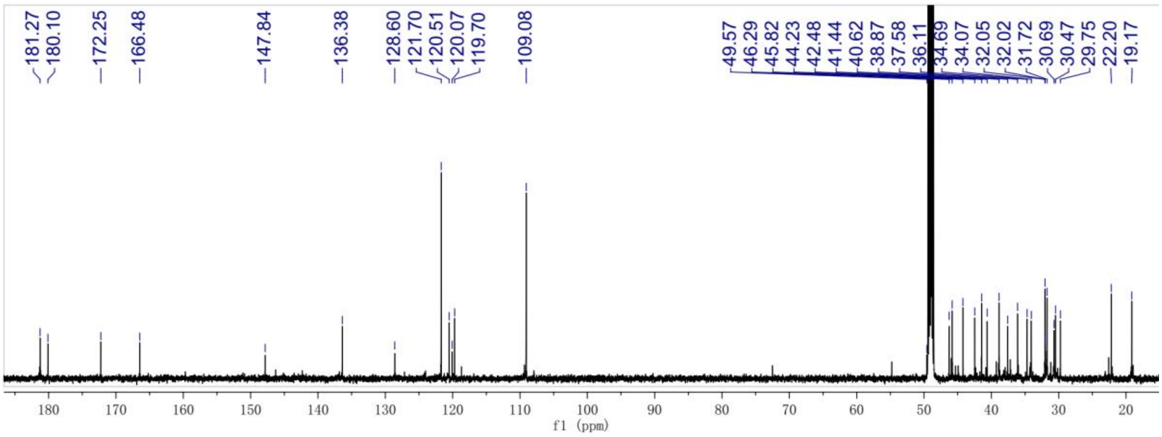

Fig. S99 ^{13}C NMR spectrum of compound **4l** (MeOD, 150 MHz)

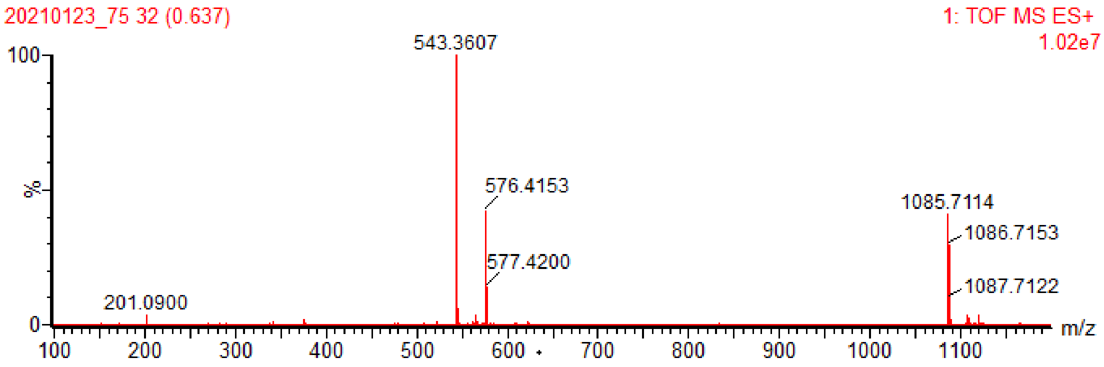

Fig. S100 HR-MS spectrum of compound **4l**

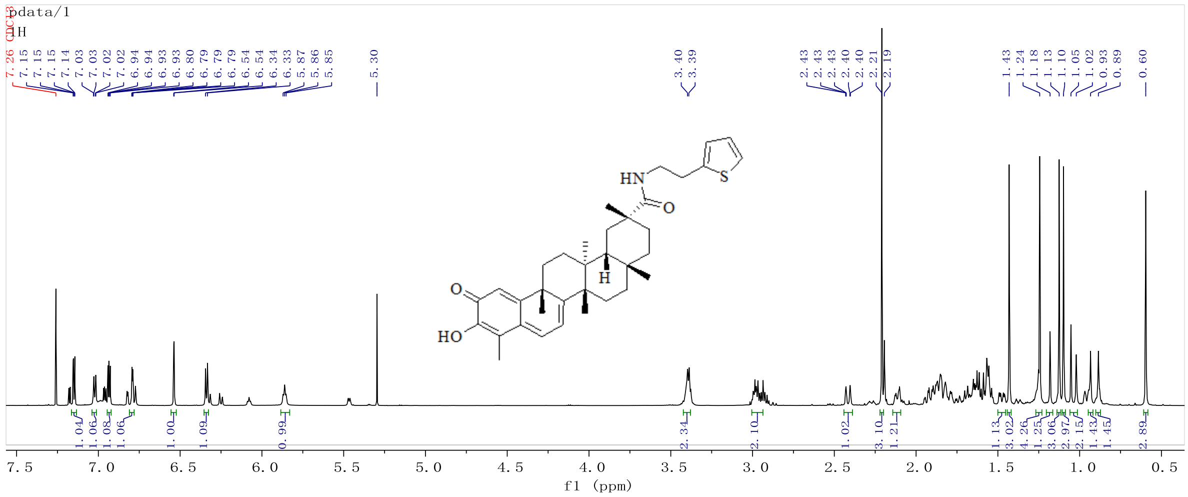

Fig. S101 ^{1}H NMR spectrum of compound **4m** ($CDCl_3$, 600 MHz)

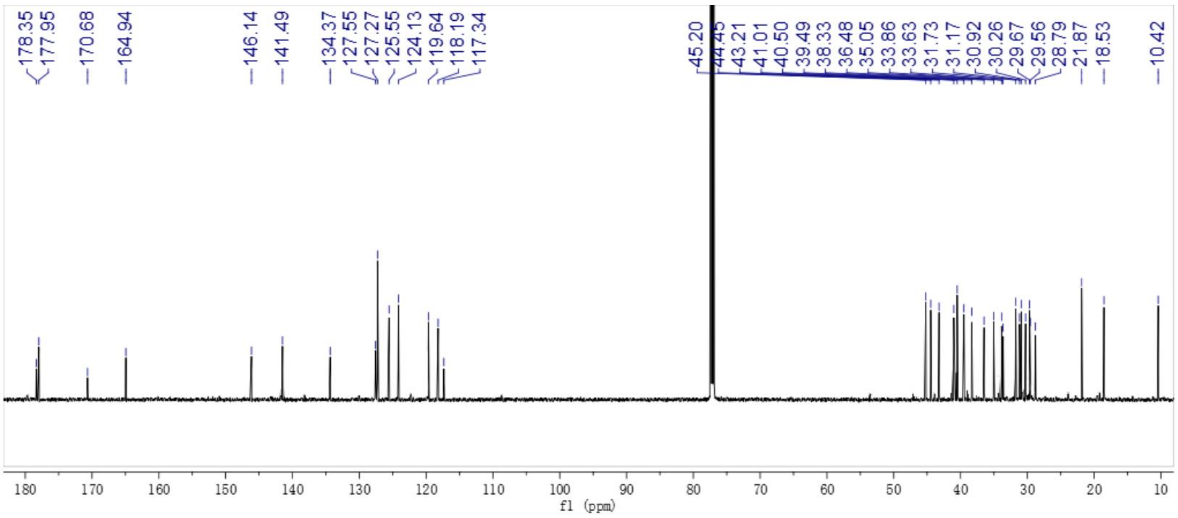

Fig. S102 ^{13}C NMR spectrum of compound **4m** ($CDCl_3$, 150 MHz)

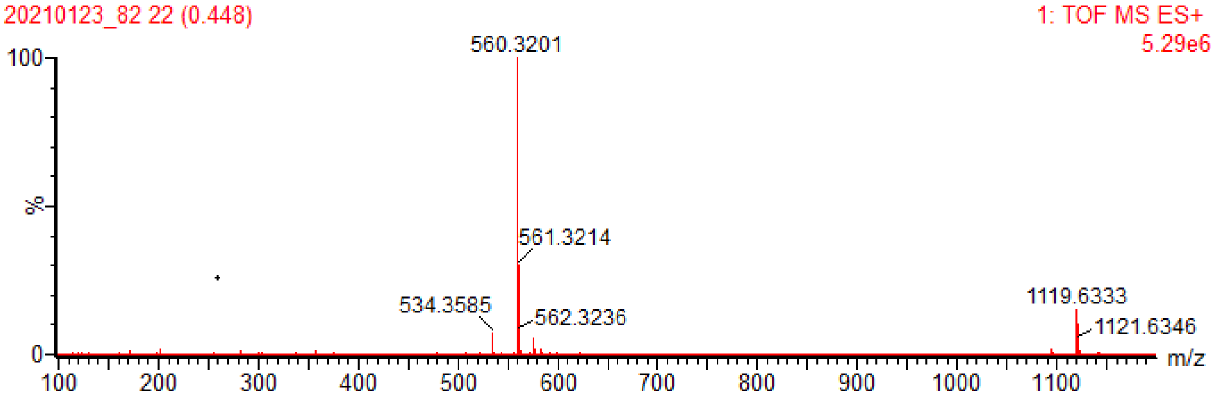

Fig. S103 HR-MS spectrum of compound **4m**

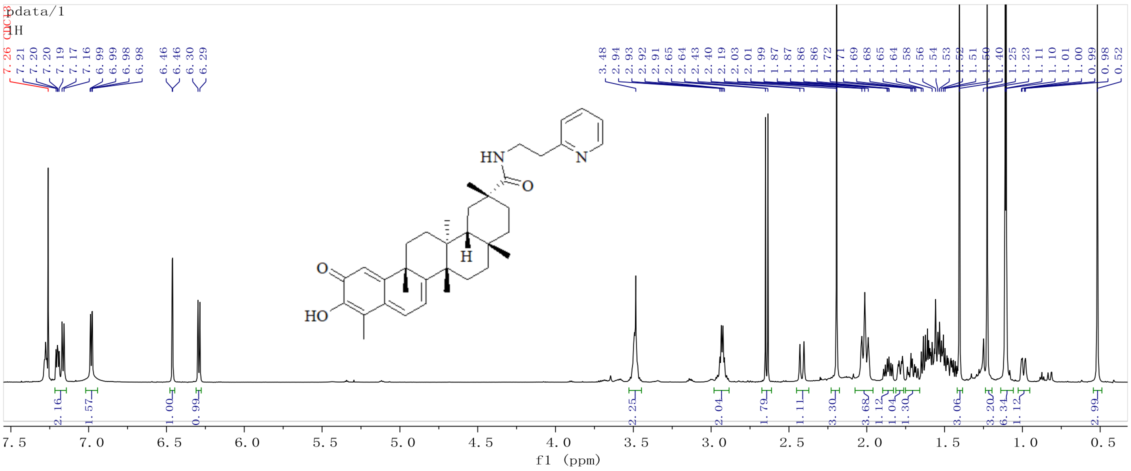

Fig. S104 ^{1}H NMR spectrum of compound **4n** ($CDCl_3$, 600 MHz)

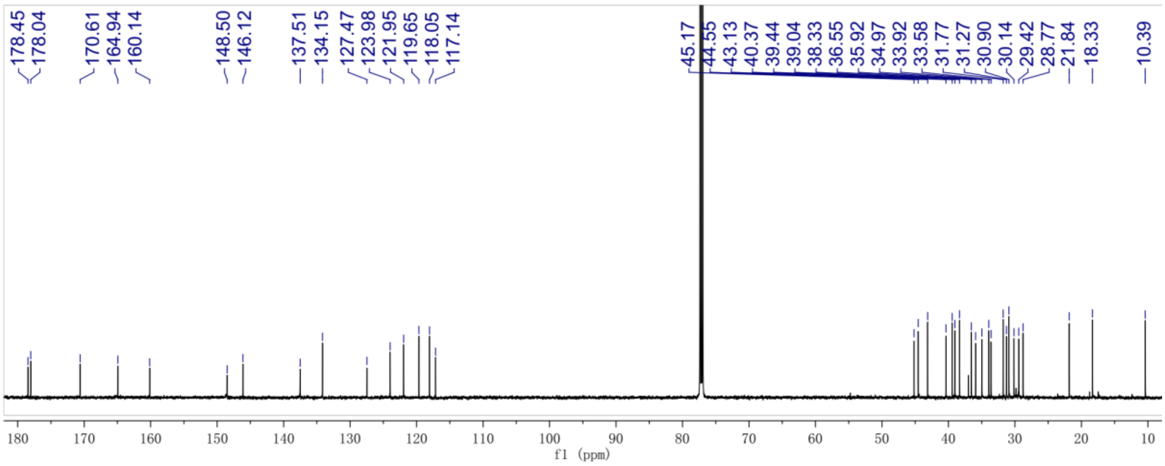

Fig. S105 ^{13}C NMR spectrum of compound **4n** ($CDCl_3$, 150 MHz)

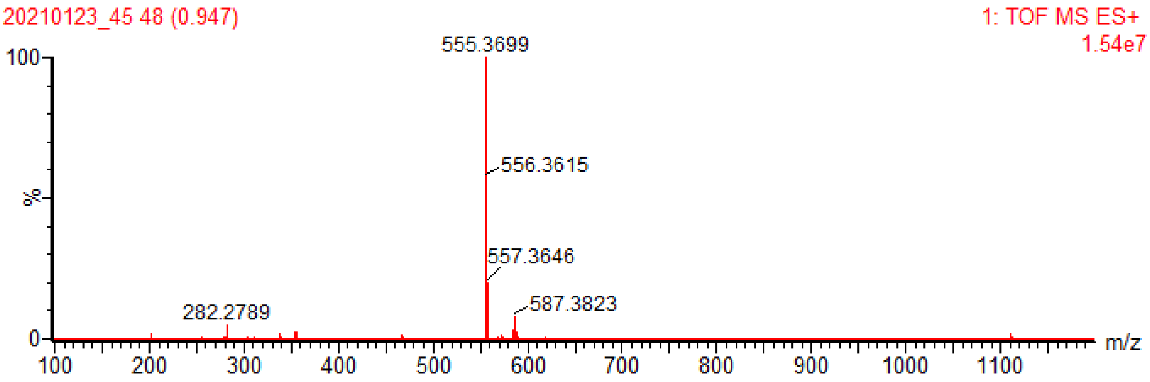

Fig. S106 HR-MS spectrum of compound **4n**
